# The shape of memory in temporal networks

**DOI:** 10.1038/s41467-022-28123-z

**Published:** 2022-01-25

**Authors:** Oliver E. Williams, Lucas Lacasa, Ana P. Millán, Vito Latora

**Affiliations:** 1 School of Mathematical Sciences, Queen Mary University of London, London, E1 4NS UK; 2 Institute for Cross-Disciplinary Physics and Complex Systems IFISC (UIB-CSIC), E-07122 Palma de Mallorca, Spain; 3 Amsterdam UMC, Vrije Universiteit Amsterdam, Department of Clinical Neurophysiology and MEG Center, Amsterdam Neuroscience, De Boelelaan 1117, Amsterdam, The Netherlands; 4 Institute Carlos I for Theoretical and Computational Physics, University of Granada, Granada, Spain; 5 Dipartimento di Fisica ed Astronomia, Università di Catania and INFN, I-95123 Catania, Italy; 6 Complexity Science Hub Vienna (CSHV), Vienna, Austria

**Keywords:** Applied mathematics, Complex networks

## Abstract

How to best define, detect and characterize network memory, i.e. the dependence of a network’s structure on its past, is currently a matter of debate. Here we show that the memory of a temporal network is inherently multidimensional, and we introduce a mathematical framework for defining and efficiently estimating the microscopic shape of memory, which characterises how the activity of each link intertwines with the activities of all other links. We validate our methodology on a range of synthetic models, and we then study the memory shape of real-world temporal networks spanning social, technological and biological systems, finding that these networks display heterogeneous memory shapes. In particular, online and offline social networks are markedly different, with the latter showing richer memory and memory scales. Our theory also elucidates the phenomenon of emergent virtual loops and provides a novel methodology for exploring the dynamically rich structure of complex systems.

## Abstract (web summary)

The evolution of networks with structure changing in time is dependent on their past states and relevant to diffusion and spreading processes. The authors show that temporal network’s memory is described by multidimensional patterns at a microscopic scale, and cannot be reduced to a scalar quantity.

## Introduction

Temporal networks^1–4^ are widely used models for describing the architecture of complex systems^5–14^. A temporal network is a network whose structure changes over time. From a mathematical viewpoint, a temporal network ${{{{{{{\mathcal{G}}}}}}}}$ with *N* nodes can be formalised as a set of *L* discrete-time stochastic processes ${{{{{{{\mathcal{G}}}}}}}}={\{{{{{{{{{\mathcal{E}}}}}}}}}^{\alpha }\}}^{\alpha = 1,2,\ldots ,L}$, where *L* ≤ *N*(*N* − 1)/2 is the number of different pairs of nodes that can be connected by links over time. Each ${{{{{{{{\mathcal{E}}}}}}}}}^{\alpha }={\{{E}_{t}^{\alpha }\}}_{t = 1,2,\ldots }$ is the stochastic process governing the dynamics of link *α*, with the random variable ${E}_{t}^{\alpha }$ taking the value ${e}_{t}^{\alpha }=1$ if link *α* is present at time *t*, and 0 otherwise. One salient and well-studied property of a system that evolves in time is that of memory, i.e. the extent to which the evolution of the system is dependent on its history. An approach based on information theory to define and estimate memory in the case of time series can be found in Section I of the SI. In the context of temporal networks, note that, in general, the stochastic processes ${{{{{{{{\mathcal{E}}}}}}}}}^{\alpha }$ might not only be dependent on their individual histories, but they can also depend on each other. Indeed, the properties of a temporal network not only depend on the patterns of activities of each of its links, but also on the ways in which these activities influence each other across the network. This observation gives rise to the notion of network memory, that is, the dependence of a temporal network’s structure on its past. Recent works have shown that network memory—whatever this is—play a prominent role in diffusion^15–18^, epidemics^19–24^ and other processes^25,26^ occurring over the temporal network, and even alters the network’s community structure^27,28^.

While recent works have proposed estimating the length of memory in a temporal network by using higher order Markov models^18,29,30^, the problem of how to define network memory is highly nontrivial and still a matter of intense debate. Note for instance that, since the set of every possible graph with *N* nodes is finite, it is in principle possible to enumerate all the configurations of a temporal network, build an alphabet accordingly, and transform the temporal network ${{{{{{{\mathcal{G}}}}}}}}$ into a time series of symbols from this alphabet. Making use of concepts from information theory, and by direct analogy to the definition of memory ${{\Omega }}({{{{{{{\mathcal{T}}}}}}}})$ for a scalar time series ${{{{{{{\mathcal{T}}}}}}}}$ (see SI Section IB), it is then possible to straightforwardly define the scalar memory ${{\Omega }}({{{{{{{\mathcal{G}}}}}}}})$ of ${{{{{{{\mathcal{G}}}}}}}}$ as the order *p* of the lowest order Markov chain that is able to reproduce the statistics of the sequence of symbols generated by ${{{{{{{\mathcal{G}}}}}}}}$ (see SI Section IIA for details). Intuitively, this approach incorporates the interdependence among the links of the network all at once. Theoretically, this “symbolisation” process could also be truncated by only including pairs of links, or triplets of links, etc in our alphabet (for instance, truncating to pairs of links would mean building an alphabet of four symbols that describe all possible four combinations of two links being present or absent at a given time). In this way, we see that ${{\Omega }}({{{{{{{\mathcal{G}}}}}}}})$ would provide the asymptotic limit of a spectrum of memories associated to finite truncations of order *m*, where for any finite truncation ${{{\Omega }}}_{m}({{{{{{{\mathcal{G}}}}}}}})$ would be the resulting memory associated to the time series of groups of *m* links (see SI Section IID for an in-depth analysis of the case *m* = 2). All in all this approach, without further constraints and limitations on the set of possible graphs, can only work for very small numbers of nodes *N* and small *m*, as the size of the alphabet grows extremely rapidly ($\sim {2}^{\frac{1}{2}({N}^{2}-N)}$) and very long time series would be required for an accurate estimate.

There is, however, a more fundamental problem with this approach. Not only is the scalar memory ${{\Omega }}({{{{{{{\mathcal{G}}}}}}}})$ (or any ${{{\Omega }}}_{m}({{{{{{{\mathcal{G}}}}}}}})$) hard to estimate, but it also fails to capture fundamental microscopic memory differences between temporal networks. Indeed, as we will show below, each temporal network is characterised by a precise pattern of memories at a microscopic scale, that we name the shape of the memory. This shape is lost when using a scalar projection like ${{\Omega }}({{{{{{{\mathcal{G}}}}}}}})$, and different memory shapes with the same ${{\Omega }}({{{{{{{\mathcal{G}}}}}}}})$ can yield different impacts on a dynamical process running on top of the network. Moreover, links can heterogeneously influence the activity of other links, and the entangled temporal dependencies among these can even bring about some virtual memory resonances in the activity of each link which, as we will show, cannot be detected by measures like ${{\Omega }}({{{{{{{\mathcal{G}}}}}}}})$ (or any ${{{\Omega }}}_{m}({{{{{{{\mathcal{G}}}}}}}})$), yet have real and measurable physical effects on network processes such as diffusion or spreading. Overall, memory has indeed a heterogeneous, multidimensional fingerprint which is not reducible to a scalar quantity.

Recently proposed alternatives to ${{\Omega }}({{{{{{{\mathcal{G}}}}}}}})$ have already focused on the microscopic effects of memory, modelling the pathways of varying length present in a temporal network by means of higher order graphs^14,18,29–31^. However, the definitions of memory proposed therein are associated with events in the network, rather than with the physical timescales over which the network evolves. In addition to this, such definitions only consider pairs of temporal links having one node in common, and are therefore more suited to capture correlations in the dynamics of links due to flows over the temporal network.

## Results

### The co-memory matrix

In order to fully characterise the shape of the memory of a temporal network, we propose to define the memory co-order ${{\Omega }}({{{{{{{{\mathcal{E}}}}}}}}}^{\alpha }| | {{{{{{{{\mathcal{E}}}}}}}}}^{\beta })$ of a pair of links *α* and *β* as the earliest time in the history of the stochastic process governing the value of ${e}_{t}^{\beta }$ which has influence on the current evolution of the process governing ${e}_{t}^{\alpha }$:

1 $${{\Omega }}({{{{{{{{\mathcal{E}}}}}}}}}^{\alpha }| | {{{{{{{{\mathcal{E}}}}}}}}}^{\beta })	= \, \mathop{\min }\limits_{p}\left[p:\right. {\mathbb{P}}({e}_{t}^{\alpha }| {\{{e}_{\tau }^{\beta }\}}_{\tau = t-1,...,t-p})\\ 	= \, \left.{\mathbb{P}}({e}_{t}^{\alpha }| {\{{e}_{\tau }^{\beta }\}}_{\tau = t-1,...})\right],$$

(see SI section IIB for details). Notice that for *α* = *β* we have ${{\Omega }}({{{{{{{{\mathcal{E}}}}}}}}}^{\alpha }| | {{{{{{{{\mathcal{E}}}}}}}}}^{\beta })={{\Omega }}({{{{{{{{\mathcal{E}}}}}}}}}^{\alpha }| | {{{{{{{{\mathcal{E}}}}}}}}}^{\alpha })={{\Omega }}({{{{{{{{\mathcal{E}}}}}}}}}^{\alpha })$. The evaluation of the whole *L* × *L* co-memory matrix ${\mathbb{M}}$, whose elements ${m}_{\alpha \beta }={{\Omega }}({{{{{{{{\mathcal{E}}}}}}}}}^{\alpha }| | {{{{{{{{\mathcal{E}}}}}}}}}^{\beta })$ are equal to the memory co-order of the pair of links *α* and *β*, allows us then to describe, at a microscopic level, the type of memory of a temporal network. The memory co-orders identify the lowest order Markov chains with the same information content as the edge time series being modelled, and indeed we show that for any temporal network the memory shape maintains a key feature of the scalar memory ${{\Omega }}({{{{{{{\mathcal{G}}}}}}}})$: it has the same information theoretic grounding (see SI sections I and II). Moreover, it represents an improvement over the state-of-the-art approaches based on pathways, which consider only pairs of links having one node in common and can only model the propagation of a message or the flow of some quantity over the temporal network^18,29^. Our framework aims to capture how the activity of a link *α* is influenced by the activity of another link *β*, no matter if the two links have a node in common or not. This can be very important in real systems, as two links *α* and *β* of a network can be active at the same time or their temporal activity correlated, even if they do not have a node in common (for instance, the activity of a given link could be causally related to the activity of another, apparently distant link, and such dependence could be mediated by a latent, non-observable link). Thus our formalism is more general than previous ones and include them as subproducts. For a detailed account on the specific implementation of this approach as well as its scaling behaviour with network size, see SI sections II and IV.

**Fig. 1:**
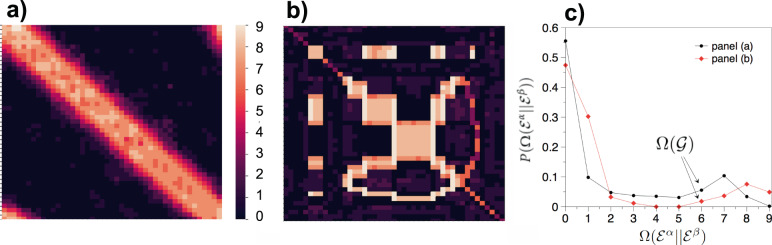
Shape of memory and emergence of virtual loops in temporal networks with correlated link dynamics. A temporal fully connected network ${{{{{{{\mathcal{G}}}}}}}}$ with *N* = 10 nodes and *L* = 45 links whose dynamics are both autocorrelated and heterogeneously cross-correlated, generated from an eCDARN(*p*) model with parameters *q* = 0.9, *y* = 0.5, *c* = 0.7 and a set of memory lengths *p* randomly sampled with uniform (panel (**a**)) or a bimodal (panel (**b**)) probability from {0, 1, …, 6} (see SI section IVA for details). **a** The 45 × 45 entries of the co-memory matrix ${\mathbb{M}}$ (shown with a colour code) display the shape of the network memory at the microscopic scale of pairs of links. In this specific case the eCDARN(*p*) model is chosen such that the causal structure of link dependencies is restricted in a Bayesian ring topology of *L* = 45 nodes, so that when link *α* samples its activity from the past history of other links, it randomly samples from *α* ± 1. The scalar memory of the network is ${{\Omega }}({{{{{{{\mathcal{G}}}}}}}})=6$. Pairs of neighbouring or close links in the Bayesian ring exhibit high memory co-order, often above ${{\Omega }}({{{{{{{\mathcal{G}}}}}}}})$, due to the onset of virtual loops (see the text), whereas distant links are seldom cross-correlated and thus display low co-order memory. **b** Similar to (**a**), but where the link’s causal structure is given by a different Bayesian graph (see SI section IVA for full details). A notably different memory shape emerges, however the scalar memory of the network is still ${{\Omega }}({{{{{{{\mathcal{G}}}}}}}})=6$. **c** Distribution of memory co-orders in both examples, showing different heterogeneous profiles which in both cases are not well characterised by ${{\Omega }}({{{{{{{\mathcal{G}}}}}}}})$.

For illustration purposes, Fig. 1a, b displays the co-memory matrices ${\mathbb{M}}$, estimated in the case of a synthetic (fully connected) temporal network with *N* = 10 nodes and *L* = 45 links with two different types of correlated dynamics. To estimate the values of *m*_*αβ*_ here (and throughout this work) we have used a modified version of the Efficient Determination Criterion (EDC)^32,33^ as this performs well as an estimator, is strongly consistent, and allows for optimised implementations (other approaches are of course possible, for details on our choice of estimators, see SI sections IC and IIC).

By construction, both models have the same scalar memory ${{\Omega }}({{{{{{{\mathcal{G}}}}}}}})=6$. However, they show very peculiar and distinct memory shapes (panels (a) and (b)), induced by both the specific inter-link dependencies that we have used and the pre-specified set of memory length parameters. This is further highlighted in the heterogeneous distribution of memory co-orders reported in panel (c) of the same figure, where it is clear that memory cannot indeed be characterised by the value of the scalar memory ${{\Omega }}({{{{{{{\mathcal{G}}}}}}}})$ alone.

### Emergence of virtual loops

Observe in Fig. 1 that we unexpectedly find some very long-memory contributions (indeed, above order 6). These are memory orders which have not been specifically pre-defined in the generative models we have used, and are a manifestation of what we name virtual loops (VLs). As we will show (full details can be found in SI Sections III and IV), VLs emerge e.g. when link *α* depends on the past of link *β*, and link *β* in turn depends on the past of link *α*, inducing a long-memory loop in the activities of each link, when they are considered separately. While being virtual in the sense that they are not pre-specified by the model nor captured by ${{\Omega }}({{{{{{{\mathcal{G}}}}}}}})$ or any ${{{\Omega }}}_{m}({{{{{{{\mathcal{G}}}}}}}})$, they do indeed play an important and measurable role in the dynamics of a temporal network and affect processes occurring on it. Mathematically, these virtual loops emerge when the Bayesian network describing the causal dependencies of link activities—the network which explicitly states which links influence which other links—is a cyclic graph, and are indeed reminiscent of other forms of causal loops appearing in other physical systems (see SI section IIIB and C for a discussion).

**Fig. 2:**
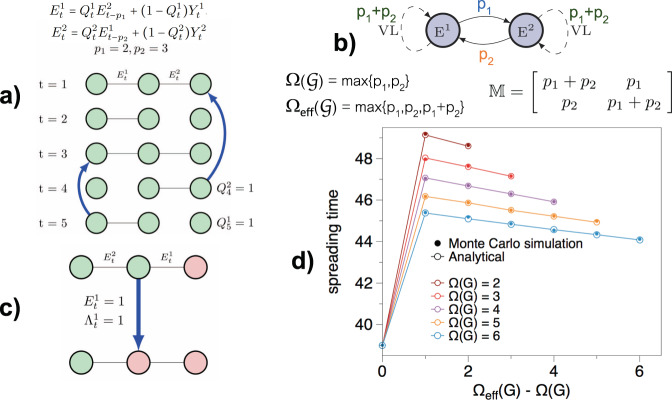
Virtual loops and their effects on spreading processes in temporal networks. **a** A sketch of five steps of the temporal evolution of a network with three nodes and two links evolving according to the displayed equation (for details see SI section IIIA). If ${Q}_{t}^{\ell }=0$ then link *ℓ* at time *t* is generated randomly. Conversely, if ${Q}_{t}^{\ell }=1$ link *ℓ* copies a state from the past, namely link 1 copies the value of link 2 at time *t* − 2, while link 2 copies the value of link 1 at time *t* − 3. **b** Bayesian graph of the causal dependencies between the two links. Link 1 copies from the past of link 2 (*p*_1_), whereas link 2 copies from the past of link 1 (*p*_2_), thereby inducing first-order virtual loops in the memory of links 1 and 2, which virtually copy from their own past, *p*_1_ + *p*_2_ steps back. The co-memory matrix ${\mathbb{M}}$ whose entries are the co-memory order of each pair of links is also shown. The scalar memory of the process is ${{\Omega }}({{{{{{{\mathcal{G}}}}}}}})=\max \{{p}_{1},{p}_{2}\}$ (for a rigorous proof, see SI section IIIA), whereas the effective memory ${{{\Omega }}}_{{{{{\mathrm{eff}}}}}}({{{{{{{\mathcal{G}}}}}}}})$, obtained as the largest entry of the co-memory matrix, is *p*_1_ + *p*_2_, which differs from ${{\Omega }}({{{{{{{\mathcal{G}}}}}}}})$ due to the existence of the virtual loops. **c** A SI (Susceptible-Infectious) epidemic spreading is defined over the temporal network. Each node can either be in the infected (red) or susceptible (green) state. If at time *t* there is a link ${E}_{t}^{\ell }=1$ between an infected node and a susceptible one, then the infection will be passed with some probability (if random variable Λ_*t*_ = 1) and the susceptible node will become infected (see SI section IIIE for details). **d** Analytical and numerical results for the average time taken for every susceptible to become infected, as a function of the difference between the scalar memory ${{\Omega }}({{{{{{{\mathcal{G}}}}}}}})$ and the effective memory ${{{\Omega }}}_{{{{{\mathrm{eff}}}}}}({{{{{{{\mathcal{G}}}}}}}})$ (this latter being extracted from ${\mathbb{M}}$). For each curve, the value of ${{\Omega }}({{{{{{{\mathcal{G}}}}}}}})$ is fixed to a constant value *p*_1_, while the value of ${{{\Omega }}}_{{{{{\mathrm{eff}}}}}}({{{{{{{\mathcal{G}}}}}}}})={p}_{1}+{p}_{2}$ is varied by changing *p*_2_ ≤ *p*_1_. We find that the spreading times depend on the value of the effective memory ${{{\Omega }}}_{{{{{\mathrm{eff}}}}}}({{{{{{{\mathcal{G}}}}}}}})$, which is then a better descriptor of the effects of memory than ${{\Omega }}({{{{{{{\mathcal{G}}}}}}}})$, this latter quantity being unable to detect any of these effects. Numerical results are obtained as averages over 10^7^ realisations of the network, and are in perfect agreement with the analytical prediction (see SI section IIIF for the full analysis).

To better illustrate the onset and role of VLs, Fig. 2(a) and (b) show an example of a toy model of a temporal network with only three nodes and two links. The model allows us to tune the shape of the memory, while the scalar memory ${{\Omega }}({{{{{{{\mathcal{G}}}}}}}})$ of the network is kept fixed. The adopted causal dependencies between the two links (each link can copy from the past of the other link) induce virtual loops. These govern the two diagonal terms of the co-order matrix ${\mathbb{M}}$ and have measurable and important effects on dynamical processes—e.g., the spread of an epidemic—taking place over the network. Figure 2c, d shows that the time taken for an infection to spread over the entire network can indeed be very different in networks with the same value of ${{\Omega }}({{{{{{{\mathcal{G}}}}}}}})$, but with different memory shapes. Additional analysis of toy models with emergent VLs are described in SI section IIIA.

Furthermore, it is easy to prove (see Theorem 1 in SI Section II-A and B) that ${{\Omega }}({{{{{{{\mathcal{G}}}}}}}})\le \mathop{\max }\nolimits_{\alpha ,\beta }\{{m}_{\alpha \beta }\}:= {{{\Omega }}}_{{{{{\mathrm{eff}}}}}}({{{{{{{\mathcal{G}}}}}}}})$, that is, the scalar memory is bounded from above by the maximum co-order over all link pairs, which we term the effective memory of the network. Figure 2(d) shows that ${{{\Omega }}}_{{{{{\mathrm{eff}}}}}}({{{{{{{\mathcal{G}}}}}}}})$ accounts for the virtual loops in the toy network model and thus captures the measurable differences in the spreading times. Of course, ${{{\Omega }}}_{{{{{\mathrm{eff}}}}}}({{{{{{{\mathcal{G}}}}}}}})$ is still not able to account for the rich memory heterogeneity of a temporal network (see panel (c) of Fig. 1), but is (i) better conceptually defined than ${{\Omega }}({{{{{{{\mathcal{G}}}}}}}})$ as it captures the effect of virtual loops, and (ii) can be computed efficiently from ${\mathbb{M}}$. These results also illustrate that the microscopic memory structure—the memory shape—has an important impact on the outcome of epidemic processes running on top of the temporal network. We expect a similar effect in other epidemic models and in other models of diffusion and also in nonlinear models such as synchronisation.

### High-order VLs and loop decoherence

The causal relationship between the links activities can be described by a so-called Bayesian graph, where the nodes represent the links of the temporal network, and two nodes are connected by a directed edge of there’s a causal relation between the two links in the temporal network. When such Bayesian graph is cyclic, then virtual loops are expected to emerge; however, this is a sufficient but not necessary condition. Indeed, when links dynamics are also autocorrelated (besides link dependencies, links have also an internal dynamics with memory) then VLs can emerge even for acyclic Bayesian graphs. This is due to the fact that the interplay between the auto- and cross-correlated dynamics can also induce (virtual) links in the Bayesian graph, which make a priori acyclic Bayesian graphs effectively cyclic (see SI section IIIB for details on the existence conditions of VLs).

At the same time, VLs can be categorised in different orders. It is easy to see that virtual loops emerging in the toy model discussed in Fig. 2 are of “order 1”, since the Bayesian graph in panel (b) only consists of two vertices, i.e. there is only a pair of links causally connected in the actual temporal network. By construction, these VLs cannot be detected by ${{\Omega }}({{{{{{{\mathcal{G}}}}}}}})$ or any ${{{\Omega }}}_{m}({{{{{{{\mathcal{G}}}}}}}})$.

Now, it is then possible to construct “higher order” virtual loops simply by adding longer causal cycles in the Bayesian graph associated to a given temporal network model (see SI Fig S2 for examples). In theory, higher order loops induce contributions to the co-memory matrix of increasingly longer memory, yielding a potentially unbounded effective memory (a memory blow up). In practice, virtual loops with high order memory are difficult to observe due to extremely long series being required to capture such effects. This is not only an issue of estimation, but has practical consequences as well: any dynamical process (e.g., an epidemic process) running on top of such a network might in practice not sense very high order VLs as the timescale in which the dynamical process takes place can be much smaller than the timescale over which high order VLs manifest. On the other hand, these VLs are stable when the underpinning temporal dependencies are fine tuned, and quickly dissipate otherwise. Altogether, these arguments suggest that only short virtual loops will typically be observed in practice, and only in certain conditions where the link dependencies are stable over long periods of time. In real-world networks such conditions might not be always satisfied and the effect of VLs might be reduced, a phenomenon we label virtual loop decoherence. In SI section III we introduce another toy model, similar to the one presented in Fig. 2, but where virtual loops are shown to dissipate (see SI section IIID for the in-depth analysis of virtual loop decoherence in the toy model).

As a summary, the memory of a temporal network, including VL effects, can be fully described by the co-memory matrix ${\mathbb{M}}$. If one wants to extract a single scalar, the correct quantity to estimate is ${{{\Omega }}}_{{{{{\mathrm{eff}}}}}}({{{{{{{\mathcal{G}}}}}}}})$ (which derives from ${\mathbb{M}}$ and takes into account VLs) and not ${{\Omega }}({{{{{{{\mathcal{G}}}}}}}})$, except for those cases where virtual loops are absent or they are decoherent. In such cases ${{\Omega }}({{{{{{{\mathcal{G}}}}}}}})$ indeed approaches ${{{\Omega }}}_{{{{{\mathrm{eff}}}}}}({{{{{{{\mathcal{G}}}}}}}})$ (see SI section IV for a thorough exposition of virtual loop decoherence).

### Validation in synthetic networks

We have tested the accuracy of our memory shape estimator in four generative temporal network models, each of varying complexity and with differing memory shapes. While we lack analytical expressions for the memory shape, in all these models we were able to obtain ${{\Omega }}({{{{{{{\mathcal{G}}}}}}}})$ analytically (i.e. a ‘ground truth’), so our computational estimation can also examine the measurable effect of virtual loops, as evidenced when ${{{\Omega }}}_{{{{{\mathrm{eff}}}}}}({{{{{{{\mathcal{G}}}}}}}}) \, > \, {{\Omega }}({{{{{{{\mathcal{G}}}}}}}})$. (i) First, we consider the so-called DARN(*p*) and eDARN(*p*_*j*_) models^23^, where all links have independent yet autocorrelated dynamics. By design these temporal network models are free from virtual loops, thus we expect ${{{\Omega }}}_{{{{{\mathrm{eff}}}}}}({{{{{{{\mathcal{G}}}}}}}})\approx {{\Omega }}({{{{{{{\mathcal{G}}}}}}}})$. (ii) In a second step we introduce the CDARN(*p*) and eCDARN(*p*) models^34^, where link dynamics are not only autocorrelated but also cross-correlated, since in these models links can sample their next state from either their own history or from the history of other chosen links. Virtual loops are expected to emerge in these cases, inducing ${{{\Omega }}}_{{{{{\mathrm{eff}}}}}}({{{{{{{\mathcal{G}}}}}}}}) \, > \, {{\Omega }}({{{{{{{\mathcal{G}}}}}}}})$ (see Fig. 1 for an illustration of the eCDARN(*p*) model and SI Section IV-A for full details of all four models, precise theorem statements and analytical derivations of the scalar memory).

**Fig. 3:**
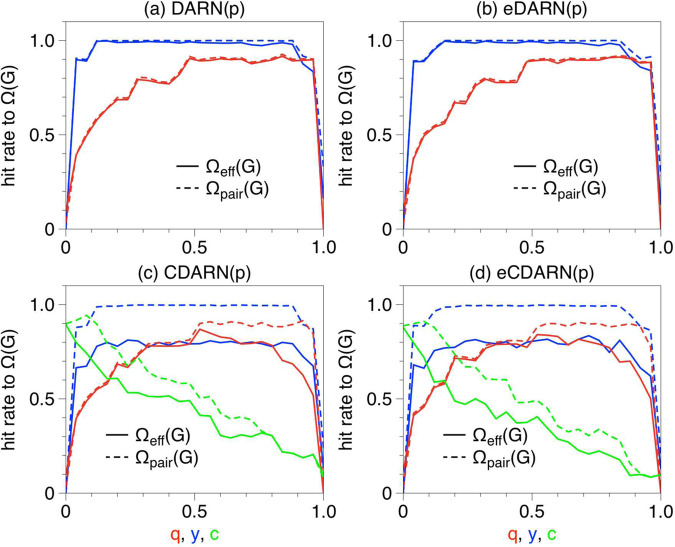
Hit rates to scalar memory ${{\Omega }}({{{{{{{\mathcal{G}}}}}}}})$ for synthetic models. In each case we compute the percentage of the times within an ensemble of 10^3^ realisations that the estimated effective memory ${{{\Omega }}}_{{{{{\mathrm{eff}}}}}}({{{{{{{\mathcal{G}}}}}}}})$ and estimated pair memory ${{{\Omega }}}_{{{{{\mathrm{pair}}}}}}({{{{{{{\mathcal{G}}}}}}}})$ exactly match the scalar memory ${{\Omega }}({{{{{{{\mathcal{G}}}}}}}})$ (the memory parameter *p* is randomly sampled from UNIFORM{1,...,10} for each realisation). Models depend on parameters *q*, *y* and (where applicable) *c* (see SI section IV for details), so each curve scans hit rates for the whole range of a given parameter and fix the values of the other parameters to *q* = 0.9, *y* = 0.1, *c* = 0.1 (in every case, time series size is *T* = 10^6^). In DARN(*p*) and eDARN(*p*) models (panels **a** and **b**) where virtual loops are by construction absent, ${{{\Omega }}}_{{{{{\mathrm{eff}}}}}}({{{{{{{\mathcal{G}}}}}}}})={{{\Omega }}}_{{{{{\mathrm{pair}}}}}}({{{{{{{\mathcal{G}}}}}}}})$ and their estimation typically coincide with ${{\Omega }}({{{{{{{\mathcal{G}}}}}}}})$ for a large range of model parameters, as expected. In CDARN(*p*) and eCDARN(*p*) models (panels **c** and **d**), (probabilistic) virtual loops are expected to kick in, inducing a mismatch between ${{{\Omega }}}_{{{{{\mathrm{eff}}}}}}({{{{{{{\mathcal{G}}}}}}}})$ and ${{\Omega }}({{{{{{{\mathcal{G}}}}}}}})$ (the mismatch is notably smaller for ${{{\Omega }}}_{{{{{\mathrm{pair}}}}}}({{{{{{{\mathcal{G}}}}}}}})$ as this quantity disregards diagonal entries of the co-memory matrix and thus cannot account for first-order virtual loops).

In every case we generate 10^3^ realisations of each temporal network model, where each networks topology consist of a fully connected backbone of *N* = 10 nodes and *L* = 45 links, with randomly chosen ground truth scalar memory and a range of different parameter configurations). We then count the hit rate (percentage of correct predictions) between the estimated ${{{\Omega }}}_{{{{{\mathrm{eff}}}}}}({{{{{{{\mathcal{G}}}}}}}})$ and the analytical value of ${{\Omega }}({{{{{{{\mathcal{G}}}}}}}})$. For completeness, we also computed the memory associated to the time series of pairs of links, i.e. ${{{\Omega }}}_{m}({{{{{{{\mathcal{G}}}}}}}})$ with *m* = 2, which we rename ${{{\Omega }}}_{{{{{\mathrm{pair}}}}}}({{{{{{{\mathcal{G}}}}}}}})$ (SI Section IID for theoretical details on ${{{\Omega }}}_{{{{{\mathrm{pair}}}}}}({{{{{{{\mathcal{G}}}}}}}})$). Results are reported in Fig. 3 (see also SI Section IVA and IVB for full details). For long enough temporal series the hit rate is consistently 100% in models which are free from virtual loops, suggesting that not only is our estimator accurate, but that in these cases ${{{\Omega }}}_{{{{{\mathrm{eff}}}}}}({{{{{{{\mathcal{G}}}}}}}})={{\Omega }}({{{{{{{\mathcal{G}}}}}}}})$. In those models where virtual loops emerge, the hit rate decays as expected. Interestingly, in a variety of cases a high hit rate is still maintained despite the presence of virtual loops of high order: we are witnessing virtual loop decoherence at play.

Moreover, another interesting phenomenon happens in some of these models when the size of the networks grows: all virtual loops are eventually dissipated. This result is rigorous for the specific case of the CDARN(p) model (see Theorem 6 in SI Section IVD). In other words, making the temporal networks large has the effect of destroying the virtual loops. We therefore conjecture that in real-world temporal networks, which are typically large, we should expect that the effective and scalar memory should be similar, and that the former, which is computed from the co-memory matrix, is a good estimate of the overall memory, while finer internal structure will be provided by the shape of ${\mathbb{M}}$.

### Analysis of real-world temporal networks

**Fig. 4:**
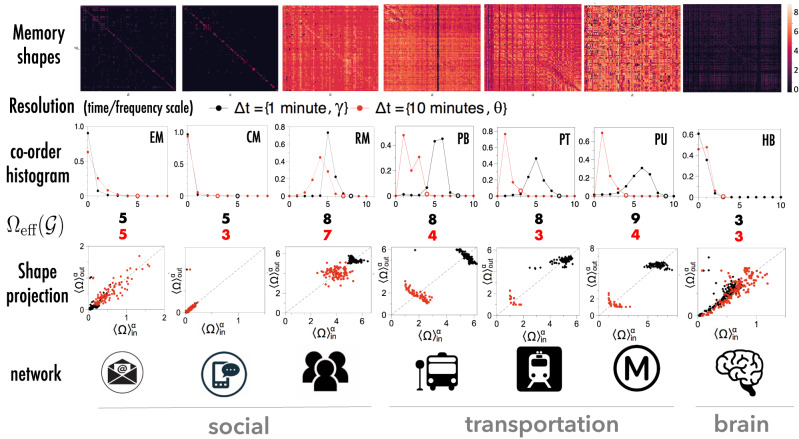
The shape of memory of real-world temporal networks. For each temporal network, we estimate the shape of the memory ${\mathbb{M}}$ restricted to the 100 most frequently active links, and plot the respective heat maps (lighter colour means higher memory). From these we extract the distribution of memory co-orders (co-order histograms for the full set of networks studied, and the characterisation of their heterogeneities can be found in SI V-B,C). The effective network memory ${{{\Omega }}}_{{{{{\mathrm{eff}}}}}}({{{{{{{\mathcal{G}}}}}}}})$ is also highlighted by hollow circles, and the actual values are reported below the plots. Networks have been sampled at two different temporal resolutions Δ*t*, namely every 1 and 10 min, or two different frequencies (gamma and theta bands) in the case of HB (heat maps only show the Δ*t* = 1 min resolution and gamma band). In the two online social networks (EM and CM) the distributions of memory co-orders concentrate around zero and decay rapidly, indicating very short memory overall, except for a few pairs of links. In the offline university social network (RM) we find instead two clear peaks corresponding to the presence of memory at two timescales of about 5 and 40-50 min (corresponding to interactions during lecture room changes and during the lectures) respectively. Two peaks are also observed in the three engineered networks. However, both peaks are compatible with a timescale of 5–7 min in PT and PU, suggesting that such systems exhibit only one effective timescale, due to enforced planning and scheduling. The bus network (PB) in addition to the 5–7 min also shows a memory timescale of about 30 min, possibly due to external phenomena such as collective delays induced by traffic jams. In the human brain (HB), a peak emerges at memory order 1, and for the theta band only. The distribution of points in the (${ \langle {{\Omega }} \rangle }_{{{{{\mathrm{in}}}}}},{ \langle {{\Omega }} \rangle }_{{{{{\mathrm{out}}}}}}$) plane (shape projection, see the text for details) allows us to distinguish networks and classify links as influencers (above the diagonal) and followers (below diagonal) and to spot outliers (see SI V-D for statistics relating to the distribution of these points in the plane).

We have finally studied the shape of memory in a large set of real-world social, technological and biological temporal networks, including (i) online email (EM) between the employees at a construction company^35^ and SMS (text messages) communication (CM) between college students^36^), (ii) (offline) social contacts at a US university from the Reality Mining experiment (RM)^37^, (iii) transportation systems (bus, train, and underground in different European cities^38^), (iv) proximity networks extracted from football matches (https://github.com/metrica-sports/sample-data), and (v) human brain functional cortical networks (HB) extracted from EEG recordings in subjects performing a motor task^25^. See SI Section VA for details on datasets. A summary of the results for a subset of networks is shown in Fig. 4 (see SI Sections VB, VC, VD, VE for an in-depth analysis). Note that only the 10^2^ most active links for each network, i.e. those with largest value of ${\sum }_{t}{E}_{t}^{\alpha }$, have been considered when constructing the co-memory matrix ${\mathbb{M}}$. The coloured heat maps in the top row of Fig. 4 indicate that memory shapes vary across networks. Overall, we found that memory is notably longer in offline networks than in online ones. This can be explained by the fact that offline social interactions are more mediated by tight schedules, which facilitate the emergence of various orders of memory. Shown also are the memory co-order histograms, i.e. the distributions of the entries in ${\mathbb{M}}$ (estimation of the effective memory is robust with respect to both finite-size and non-stationarity effects of the temporal networks, and fine-grained fluctuations in the microscopic memory structure due to non-stationarities in the data can be captured by the memory shape, see SI section VE for analysis and discussion).

In order to detect memory at different timescales, we have also sampled social and transportation networks at two temporal resolutions, namely Δ*t* = 1 and Δ*t* = 10 min, and considered human brain EEG recordings at two frequencies (again, see SI Section VA for details). The results should be interpreted accordingly: notice for instance, that order 2 at the Δ*t* = 1 resolution is equivalent to a memory length of 2 min, whereas order 2 at the Δ*t* = 10 resolution is equivalent to a memory length of 20 min. We have found that, while in the transportation networks with tight scheduling only one memory scale flags up, in the case of the bus network, whose scheduling could be more affected by external factors such as traffic jams, and in the case of the offline social network, at least two different memory scales show up. The situation is particularly clear in the case of human contacts at university, which show memory lengths of 5 and 40–50 min corresponding to *different* types of mechanisms of recurrent social interactions during lectures and in between lecture room changes. We also see evidence for different scales in the human brain: while the gamma band links are predominantly memoryless, for the theta band we observe a peak at memory order 1, corresponding to 1 μs.

The distribution of the entries in ${\mathbb{M}}$ tells us about the timescales of all the interactions that are present in a temporal network. In Section SI VC we explore the shape of these distributions more closely by measuring their entropy and kurtosis, providing interpretable results. We find that transportation networks tend to have large co-memory entropy, meaning that these systems display a highly heterogeneous memory kernel: many different microscopic memories are found. Likewise, the fact that online social networks tend to have a strong kurtosis speak to the fact that even if most of the links have weak memory, there are a selected few whose co-order is very large. This suggests that while online social networks have a large (scalar) effective memory ${{{\Omega }}}_{{{{{\mathrm{eff}}}}}}({{{{{{{\mathcal{G}}}}}}}})$, this only comes from the contribution of a handful of links, while microscopically the system has overall weak memory.

Information that is present in the co-order matrix can be extracted by looking at various other projections, which distill the matrix into something easily interpretable. For instance, we have considered the projections obtained when computing the average incoming and outgoing co-order ${ \langle {{\Omega }} \rangle }_{\,{{{{\mathrm{in}}}}}\,}^{\alpha }={\left[{L}^{-1}{\mathbb{M}}\underline{1}\right]}^{\alpha }$ and ${ \langle {{\Omega }} \rangle }_{\,{{{{\mathrm{out}}}}}\,}^{\alpha }={\left[{L}^{-1}{{\mathbb{M}}}^{T}\underline{1}\right]}^{\alpha }$ for each link *α* (see SI Section VD for details and the full analysis). These respectively characterise the typical length of the memory that link *α* retains of the past activity of the whole network (in), and the average length of the past history of *α* that influences the evolution of the network (out). The scatter plots in Fig. 4 (shape projection) allow us to classify and partition links (see also SI Section VD for additional plots and analysis). For instance, we can divide them into two main categories: influencers (whose past activity has more influence on the rest of the network than the network has on them), and followers (which are the opposite). By construction the influencers and followers must balance each other out since ${\mathbb{E}}\left[{ \langle {{\Omega }} \rangle }_{\,{{{{\mathrm{out}}}}}\,}^{\alpha }\right]={\mathbb{E}}[{ \langle {{\Omega }} \rangle }_{\,{{{{\mathrm{in}}}}}\,}^{\alpha }]$, however we find that in almost all of the studied networks there are more followers than influencers, implying that the memory of the network is disproportionately driven by a small number of links (again, see SI VD for details). We also notice that the scatter plots are distinct on the level of classes; offline social networks present similar scatter plots to each other, as do transport networks. This similarity is also maintained at different timescales.

## Discussion

The dynamical properties of a temporal network—of which memory is a salient example—are known to have substantial effects on the processes running over the network. In this paper we have shown that memory is better characterised by a multidimensional object—the shape, than a scalar one, and we have proposed a mathematical and computational framework to estimate the memory structure of temporal networks. By doing so, we have then identified the emergence of certain memory resonances—long-memory effects—in the microscopic memory kernel of even simple temporal network models, a phenomenon we have named virtual loops. Although virtual in the sense of not being pre-specified in the models, virtual loops have a physical and observable effect on processes running on top of the network, such as spreading processes. With hindsight, these virtual loops are indeed a particular case of ‘causal loops’, and therefore share similarities with similar concepts arising in physics and computer science: from inference problems at the heart of statistical physics to the study of Feynman diagrams in quantum mechanics.

Our approach, based on the evaluation of the co-memory matrix ${\mathbb{M}}$, not only provides a sound and efficient approximation of the memory of a temporal network, but also offers a comprehensible description of its microscopic shape. Our analysis of epidemic processes running on temporal networks with a fixed scalar memory but varying memory shapes illustrate that such microscopic memory structure has an important effect on the outcome of the epidemic process. We hope that the community will further use our framework to investigate the effect of the memory shape in the outcome of other dynamical processes running on top of the temporal network. On the other hand, our analysis of real-world temporal networks has revealed a number of interesting patterns, including the presence of different timescales of interaction in empirical data, asymmetries in the contribution of links to the evolution of the network in terms of followers/influencers, and the observation that offline networks tend to have an overall richer memory structure than online ones. These results unveil that memory shapes can be very heterogeneous in real-world systems, and indicate that fully considering the rich microscopic structure of the temporal dependencies among the links is key when it comes to understanding the function of time-varying systems. We have provided an efficient implementation of the algorithms presented in this paper in several programming languages, which are available in a user-friendly setup (see Code Availability Section). We hope our work contributes to the problem of understanding the internal structure of temporal networks and will prompt further studies and applications in areas ranging from mobility in urban systems, containment of infectious diseases or information processing in the human brain^39^, to cite some.

Finally, our work poses a number of challenging questions which deserve further investigation, including a deeper understanding of how both virtual loops and in general the internal memory structure of a temporal network can influence the spread of a disease, or the diffusion of information over the network. Such understanding could then be leveraged to e.g. control the spread of an epidemic by finely controlling specific regions of the network’s memory. Additionally, observe that by construction the co-memory matrix ${\mathbb{M}}$ accounts for pairwise link interactions. It is theoretically possible that large groups of links display collective memory, e.g. the activity of a certain set of links may depend on the orchestrated activity of other sets of links. This would in principle be captured by the spectrum of memories ${{{\Omega }}}_{m}({{{{{{{\mathcal{G}}}}}}}})$ as discussed in the Introduction, but further research is needed to understand how such spectrum can be approximated, and what new information this sort of “simplicial memory”^40^ could provide on the behaviour and evolution of complex systems.

## Supplementary information

Supplementary Information (PDF)

## Data Availability

Data associated with this study can be found via the following links: e-mail communication: www.ii.pwr.edu.pl/m̃ichalski/, text messages between college students: snap.stanford.edu/data/CollegeMsg.html, reality mining experiment: http://realitycommons.media.mit.edu/realitymining.html, public transport data: www.nature.com/articles/sdata201889, football data: https://github.com/metrica-sports/sample-data.

## Code availability

Complete implementations of our general method and all examples are available in C++, Python 2.7, Python 3.6, Java and Rust at github.com/oewilliams/temp-net-memory, DOI 10.5281/zenodo.5775001 (see also SI section VI).
