# Supplementary material for: The shape of memory in temporal networks
Source: Nat Commun. 2022 Jan 25;13:499. doi: 10.1038/s41467-022-28123-z (PMC8789900; doi:10.1038/s41467-022-28123-z)
Supplement: Supplementary file 1 — Supplementary Information (PDF) [file 41467_2022_28123_MOESM1_ESM.pdf]

Supplementary information for "The shape of memory in temporal networks"

Oliver E. Williams,[1] Lucas Lacasa,[1,2] Ana P. Millán,[3,4] and Vito Latora[1,5,6]

[1]School of Mathematical Sciences, Queen Mary University of London, London, E1 4NS, United Kingdom
[2]Institute for Cross-Disciplinary Physics and Complex Systems IFISC (UIB-CSIC), Palma de Mallorca, Spain
[3]Amsterdam UMC, Vrije Universiteit Amsterdam, Department of Clinical Neurophysiology and MEG Center, Amsterdam Neuroscience, De Boelelaan 1117, Amsterdam, The Netherlands
[4]Institute Carlos I for Theoretical and Computational Physics, University of Granada, Spain
[5]Dipartimento di Fisica ed Astronomia, Università di Catania and INFN, I-95123 Catania, Italy
[6]Complexity Science Hub Vienna (CSHV), Vienna, Austria

CONTENTS

I. The memory of a time series 2
A. Random variables and entropies 2
B. Entropy and memory of a time series 2
C. Estimating the memory 3

II. Defining and quantifying the memory of a temporal network 4
A. The scalar memory $\Omega(\mathcal{G})$ 4
B. The shape of the memory: the co-memory matrix $\mathbb{M}$ and the effective memory $\Omega_{\text{eff}}(\mathcal{G})$ 5
C. Estimating the co-memory matrix $\mathbb{M}$ 7
D. Pair Memory $\Omega_{\text{pair}}(\mathcal{G})$ 8

III. Why the co-memory matrix $\mathbb{M}$ and the effective memory $\Omega_{\text{eff}}(\mathcal{G})$ are better defined and more useful concepts than the scalar memory $\Omega(\mathcal{G})$ 9
A. Two toy models and their virtual loops 9
B. Virtual loops of arbitrary order and conditions for VLs to emerge 12
C. Virtual loops in other areas of physics and beyond 12
D. The phenomenon of virtual loop decoherence 13
E. Epidemic Spreading defined on top of models 1 and 2 14
F. Analytical solutions of the SI dynamics on network models 1 and 2 15
G. Inter-event time statistics 17

IV. Validating the framework on synthetic temporal networks 18
A. Model definitions and ground truth proofs for $\Omega(\mathcal{G})$ 18
B. Estimator accuracy on synthetic networks 21
C. Virtual loops in the synthetic networks 23
D. A theorem on full virtual loop decoherence effect for large network size 23

V. Applications to real-world temporal networks 24
A. Processing empirical network data 24
B. Co-order histograms and memory shapes at different time scales 26
C. Heterogeneity of co-memory histograms: entropy and kurtosis 29
D. Constructing memory communities and comparing networks in the $(\langle\Omega\rangle_{\text{in}}^{\alpha}, \langle\Omega\rangle_{\text{out}}^{\alpha})$ plane 30
1. Transformed coordinates 34
E. Assessing the impact of finite-size effects and non-stationarities in the memory estimation 35

VI. Method implementations 38

Supplementary References 39

I. THE MEMORY OF A TIME SERIES

A. Random variables and entropies

Let us consider a discrete random variable X with sample space $\mathcal{S}$ and probability mass function $\mathbb{P}(x) = \text{Prob}\{X = x\}$ with $x \in \mathcal{S}$. The entropy $H(X)$ of the random variable X is defined in terms of the probabilities $\mathbb{P}(x)$ of observing $x \in \mathcal{S}$ as:

$$H(X) = -\sum_x \mathbb{P}(x) \log \mathbb{P}(x) . \tag{1}$$

The definition of entropy can be extended to a pair or more discrete random variables. Let Y be a second discrete random variable with sample space $\mathcal{S}'$. The joint entropy of the pair X, Y is given by:

$$H(X,Y) = -\sum_{x,y} \mathbb{P}(x,y) \log \mathbb{P}(x,y) , \tag{2}$$

where $\mathbb{P}(x,y) = \text{Prob}\,(\mathrm{X = x, Y = y})$ is the joint distribution of the two random variables. We can also define the entropy of the random variable X when it is conditioned on the second discrete random variable Y as:

$$H(X|Y) = \sum_x \mathbb{P}(x) H(X|Y=y) = -\sum_x \mathbb{P}(x) \sum_y \mathbb{P}(y|x) \log p(y|x) = -\sum_{x,y} \mathbb{P}(x,y) \log \mathbb{P}(x|y) . \tag{3}$$

B. Entropy and memory of a time series

A time series $\mathcal{T} = \{X_t\}_{t=0,1,...}$ or simply $\{X_t\}$ is a time-discrete stochastic process in which, at each time step t, with $t = 0, 1, 2, \ldots$, the random variable X_t takes values in state space $\mathcal{S}$. We will indicate as x_t the realization of random variable X_t, i.e. the value taken at time t by time series.

The entropy rate H of the time series $\mathcal{T}$ can be defined as:

$$H(\mathcal{T}) = \lim_{n\to\infty} \frac{1}{n} H(X_0, ..., X_n) , \tag{4}$$

and the conditional entropy of $\mathcal{T}$ as:

$$H'(\mathcal{T}) = \lim_{n\to\infty} H(X_n | X_0, ..., X_{n-1}) . \tag{5}$$

If the process $\mathcal{T}$ is strongly stationary, i.e. if its joint probability distribution does not change when shifted in time [1], then it can be proven that $H(\mathcal{T}) = H'(\mathcal{T})$. For our purposes we will assume that this is always the case, allowing us to study only conditional entropies, and hence provide a concrete definition of memory. Here and in the following, for the sake of simplicity, we introduce the following notation. We denote the sequence of random variables $X_0, X_1, ..., X_n$ as $X_{0,n}$, and similarly for the realisations $x_0, x_1, ..., x_n$ we write $x_{0,n}$. Since $x_i \in \mathcal{S}\ \forall i$, then we have $x_{0,n} \in \mathcal{S}^{n+1}$.

We then define the n_{th} order block entropy H_n of the process $\mathcal{T}$ as the entropy associated with the first $n+1$ random variables $X_0, \ldots, X_n$:

$$H_n(\mathcal{T}) = -\sum_{x_{0,n}} \mathbb{P}(x_{0,n}) \log \mathbb{P}(x_{0,n}) . \tag{6}$$

Note that in particular H_0 coincides with the entropy of the marginal distribution of the first random variable X_0. Since $\mathcal{T}$ is stationary, H_0 is then the entropy associated with the marginal distribution of any of the random variables, in other words $H_0 = H(X_i), \forall i$. Similarly, for $n > 0$, H_n is the entropy of blocks of $n+1$ consecutive random variables (i.e. we are not required to consider the *first* $n+1$ random variables differently to any other set of $n+1$ consecutive random variables). Of course, the entropy rate H of the process $\mathcal{T}$ is then:

$$H(\mathcal{T}) = \lim_{n\to\infty} \frac{1}{n} H_n(\mathcal{T}) \tag{7}$$

Analogously, we can define the n_{th} order conditional entropy h_n as:

$$\begin{aligned} h_n(\mathcal{T}) =& H_n(\mathcal{T}) - H_{n-1}(\mathcal{T}) \\ &= -\sum_{x_{0,n}} \mathbb{P}(x_{0,n}) \log \mathbb{P}(x_n | x_{0,n-1}) \\ &= -\sum_{x_{0,n}} \mathbb{P}(x_n | x_{0,n-1}) \mathbb{P}(x_{0,n-1}) \log \mathbb{P}(x_n | x_{0,n-1}) . \end{aligned} \tag{8}$$

so that:

$$H'(\mathcal{T}) = \lim_{n\to\infty} h_n(\mathcal{T}) \tag{9}$$

We are now ready to define the memory of the time series $\mathcal{T}$. Informally, the memory of $\mathcal{T}$ can be thought of as the number of times steps into the past which have an influence on the next observed value. More formally, we can define the *memory length*, *memory order*, or simply *memory* $\Omega(\mathcal{T})$ of stochastic process $\mathcal{T} = \{X_t\}$ as the order p of the lowest-order Markov chain that is able to reproduce the statistics of the process, i.e. the lowest value of p such that the conditional probability mass functions satisfies:

$$\mathbb{P}(x_t|x_0, x_1, \dots, x_{t-1}) = \mathbb{P}(x_t|x_{t-p}, \dots, x_{t-1}) \tag{10}$$

for each $x_0, x_1, \dots, x_t \in \mathcal{S}^{t+1}$, or in compact form $\mathbb{P}(x_t|x_{0,t-1}) = \mathbb{P}(x_t|x_{t-p,t-1})$ for each $x_{0,t} \in \mathcal{S}^{t+1}$. This is equivalent to saying that $\mathcal{T}$ can be identified as a p-th order Markov chain, and we write: $\Omega(\mathcal{T}) = p$:

$$\Omega(\mathcal{T}) = \Omega(\{X_t\}) := \min_p \left[p : \mathbb{P}(x_t|x_{0,t-1}) = \mathbb{P}(x_t|x_{t-p,t-1})\right] \tag{11}$$

It is now possible to relate $\Omega(\mathcal{T})$ to the entropies we have introduced above. Observe first that h_n is a monotonically non decreasing function of n, i.e. $h_n \geq h_{n-1} \forall n$. Second, h_n increases with n until the point when n is precisely equal to p, and remains constant thereafter, i.e. $h_{p+d}(\mathcal{T}) = h_p(\mathcal{T})$ for any positive integer d. This can be easily demostrated, since for a p_{th} order Markov chain $\mathcal{T}$, and for $n = p + d$ with d some positive integer, we can write:

$$\begin{aligned}
h_{p+d}(\mathcal{T}) &= -\sum_{x_{0,p+d}} \mathbb{P}(x_0|x_{1,p+d})\,\mathbb{P}(x_{1,p+d}) \log \mathbb{P}(x_0|x_{1,p+d}) \\
&= -\sum_{x_{0,p+d}} \mathbb{P}(x_0|x_{1,p})\,\mathbb{P}(x_{1,p+d}) \log \mathbb{P}(x_0|x_{1,p}) \\
&= -\sum_{x_{0,p}} \mathbb{P}(x_0|x_{1,p}) \log \mathbb{P}(x_0|x_{1,p}) \sum_{x_{p+1,p+d}} \mathbb{P}(x_{1,p+d}) \\
&= -\sum_{x_{0,p}} \mathbb{P}(x_0|x_{1,p})\,\mathbb{P}(x_{1,p}) \log \mathbb{P}(x_0|x_{1,p}) \\
&= h_p(\mathcal{T})
\end{aligned} \tag{12}$$

These two conditions together mean that the n_{th} order conditional entropy will be at a maximum when we take $n = \Omega(\mathcal{T})$, i.e. $\Omega(\mathcal{T})$ coincides with the minimum value of n which maximises $h_n(\mathcal{T})$.
How should we compute the conditional entropy, and hence the memory, of a time series in practice? Ideally we would need an infinitely long realisation of $\mathcal{T}$ to be able to accurately estimate these values. For finite size time series, simply maximising h_n will not give a true estimate of the memory. Various methods have been developed to overcome such a limitation, and thus present consistent estimators for the order of a process when observing a finite time series, which we will briefly discuss in further sections.

C. Estimating the memory

We will now detail three examples of how to estimate the memory of a stochastic process from the information stored in finite time series. These estimators all make use of the information theoretic framework that we have here detailed. The aim here is to overcome the problems associated with only having a finite amount of data from which to estimate the memory via the introduction of a "penalty term". Given an observed time series (i.e. a realisation of $\mathcal{T}$) with values $(x_0, x_1, ..., x_T)$, where $x_k \in \mathcal{S}$ and the set has $|\mathcal{S}| = m$ symbols, we start by to counting how many blocks of consecutive symbols of a given size, say s, are found in the time series. We label $n_{i_1,\dots i_s}$ as the total number of times a block of s consecutive symbols with a specific arrangement for each of the s entries appears in the time series, where $i_j \in \mathcal{S},\ \forall j = 1, \dots, s$. Specifically we have

$$n_{i_1,\dots i_s} = \sum_{k=0}^{T-s+1} I(x_k = i_1, ..., x_{k+s-1} = i_s), \tag{13}$$

where I is the indicator function, so that $I(x_k = i_1, ..., x_{k+s-1} = i_s) = 1$ when $x_k = i_1, ..., x_{k+s-1} = i_s$ is true, and zero otherwise. We then define the following log likelihood function

$$\log L_t = \sum_{i_1,\dots,i_{t+1}} n_{i_1,\dots,i_{t+1}} \log \frac{n_{i_1,\dots,i_{t+1}}}{n_{i_1,\dots,i_t}}. \tag{14}$$

With this we can define the Akaike information criterion (AIC) [2], Bayesian information criterion (BIC) [3], and the optimal form of the efficient determination criterion (EDC) [4, 5], as follows:

$$\mathrm{AIC}(k) = -2\log L_k + 2m^k(m-1), \tag{15}$$

$$\mathrm{BIC}(k) = -2\log L_k + m^k(m-1)\log T, \tag{16}$$

$$\mathrm{EDC}(k) = -2\log L_k + 2m^{k+1}\log\log T. \tag{17}$$

Given some upper bound K on the order of the time series we define the corresponding estimators as

$$p_{\mathrm{AIC}} = \underset{0\le k\le K}{\arg\min}\,\mathrm{AIC}(k), \tag{18}$$

$$p_{\mathrm{BIC}} = \underset{0\le k\le K}{\arg\min}\,\mathrm{BIC}(k), \tag{19}$$

$$p_{\mathrm{EDC}} = \underset{0\le k\le K}{\arg\min}\,\mathrm{EDC}(k). \tag{20}$$

This gives us three alternatives for the estimation of the true order of the observed process. While the AIC and, to a lesser extent the BIC, are commonly used to estimate the order of a Markov chain given observed data, the EDC has been shown to be, in some sense, optimal. By optimal we mean that it is the strongly consistent estimator with the fastest convergence to the true order. It should be noted that the AIC, while the most popular, is not a consistent estimator [3], but is included here for completeness. In light of this, when estimating the order of a given stochastic process from an observed time series realisation, in this work we will always use the EDC. We remind the reader that there are also other possible alternatives, as the memory can, in practice, be estimated via different approaches, and indeed the concept of memory can be operationalised in different ways, each with their own advantages and drawbacks [2, 3, 5–8]. In this work however, we will stick with the EDC for the above reasons.

II. DEFINING AND QUANTIFYING THE MEMORY OF A TEMPORAL NETWORK

A. The scalar memory $\Omega(\mathcal{G})$

A temporal network $\mathcal{G}$ may be formally defined by the stochastic processes that generate the time evolution of its nodes and links. For simplicity, we assume here that the set of nodes is fixed, so that only the links of the network can change over time. We indicate the number of nodes with N, and we label with the index $\alpha \in \{1, 2, \ldots L\}$ each of the L node pairs that can be connected over time. In the most general case $L = N(N-1)/2$, however, smaller values of L are enough for an adequate description of the system if further topological restrictions are imposed on the backbone of the temporal network. A temporal network $\mathcal{G}$ over the N nodes can then be written as a set of L discrete-time stochastic processes $\mathcal{G} = \{\mathcal{E}^\alpha\}^{\alpha=1,2,\ldots,L}$. For each link α, with $\alpha = 1, 2, \ldots, L$, $\mathcal{E}^\alpha = \{E_t^\alpha\}_{t=1,2,\ldots}$ is the stochastic process which describes its dynamics. From now on, we will therefore indicate the network as either $\mathcal{G} = \{\mathcal{E}^\alpha\}^{\alpha=1,2,\ldots,L}$, $\mathcal{G} = \{E_t^\alpha\}_{t=1,2,\ldots}^{\alpha=1,2,\ldots,L}$ or simply as $\mathcal{G} = \{E_t^\alpha\}$.

We indicate as e_t^α the value taken by the stochastic variable E_t^α. For each α and each t, e_t^α can only assume the value 1, if link α is present at time t, or 0 otherwise. With $\mathbf{e}_t = e_t^1, e_t^2, \ldots, e_t^L$ we indicate the set of values taken by the L stochastic variables at time t. In this way $\mathbf{e}_t$ completely characterizes the state of the graph at time t.
We can introduce a first definition of the memory of a temporal network $\mathcal{G} = \{E_t^\alpha\}$ by direct analogy to the case of a scalar time series discussed in Section I. The *scalar memory order*, or *scalar memory length*, or simply *scalar memory* $\Omega(\mathcal{G})$ of the temporal network $\mathcal{G}$ can be defined as the order p of the lowest-order Markov chain able to reproduce the process, i.e. the minimum value of p such that:

$$\mathbb{P}(\mathbf{e}_t|\mathbf{e}_0, \mathbf{e}_1, \ldots, \mathbf{e}_{t-1}) = \mathbb{P}(\mathbf{e}_t|\mathbf{e}_{t-p}, \ldots, \mathbf{e}_{t-1}) \tag{21}$$

for each $\mathbf{e}_0, \mathbf{e}_1, \ldots, \mathbf{e}_t$. In compact form, this can be written as $\mathbb{P}(\mathbf{e}_t|\mathbf{e}_{0,t-1}) = \mathbb{P}(\mathbf{e}_t|\mathbf{e}_{t-p,t-1})$ for each $\mathbf{e}_{0,t}$, where by $\mathbf{e}_{0,t}$ we indicate $\mathbf{e}_0, \mathbf{e}_1, \ldots, \mathbf{e}_t$. We can finally write:

$$\Omega(\mathcal{G}) = \Omega(\{E_t^\alpha\}) := \min_p\left[p : \mathbb{P}(\mathbf{e}_t|\mathbf{e}_{0,t-1}) = \mathbb{P}(\mathbf{e}_t|\mathbf{e}_{t-p,t-1})\right] \tag{22}$$

Since the space of every possible graph with N nodes is finite, it is in principle possible to enumerate all these graphs, build an alphabet accordingly, and transform a realisation $\mathcal{G}$ into a time series of symbols from this alphabet, from which the same methods to extract the memory order of a scalar time series can be used.

B. The shape of the memory: the co-memory matrix $\mathbb{M}$ and the effective memory $\Omega_{\text{eff}}(\mathcal{G})$

Since a temporal network is here defined as the set of stochastic processes describing the dynamics of its L links, we may want to characterise, alongside the memory order $\Omega(\mathcal{G})$ of the network as a whole, the memory of each link separately, or even the memory in the influence between two links. Such a profile of the memory at the microscopic levels of the links of the network is what we will name the *shape of the memory* of the temporal network. It is therefore convenient to introduce a novel concept to capture the length of the memory of the mutual influence of two different links. In order to do this, let us consider two stationary time series $\mathcal{X} = \{X_t\}$ and $\mathcal{Y} = \{Y_t\}$. We can define the *memory co-order*, or simply *co-memory* $\Omega(\mathcal{X}||\mathcal{Y}) = \Omega(\{X_t\}||\{Y_t\})$ of the random process $\{X_t\}$ with respect to $\{Y_t\}$, as the furthest point in the past history of $\{Y_t\}$ which has influence on the value taken by X_t at time t, i.e. as the lowest value of p such that $\mathbb{P}(x_t|y_{t-1,t-p}) = \mathbb{P}(x_t|y_{t-1,-\infty})$ where $y_{0,t}$ is shorthand for the sequence $y_0, y_1, ..., y_t$. We can thus write:

$$\Omega(\mathcal{X}||\mathcal{Y}) = \Omega(\{X_t\}||\{Y_t\}) := \min_p \left[p : \mathbb{P}(x_t|y_{0,t-1}) = \mathbb{P}(x_t|y_{t-p,t-1})\right], \tag{23}$$

for each $y_{0,t-1}$ and each x_t. Since we consider processes to be stationary, this value is invariant of t. Note that if the two processes $\mathcal{X}$ and $\mathcal{Y}$ are the same, then $\Omega(\mathcal{X}||\mathcal{Y}) = \Omega(\mathcal{X}||\mathcal{X}) = \Omega(\mathcal{X})$.

We can now define the *memory co-order* or simply *co-memory* $\Omega(\mathcal{E}^\alpha||\mathcal{E}^\beta)$ of the random process $\mathcal{E}^\alpha = \{E_t^\alpha\}_{t=1,2,...}$, representing edge α, with respect to a second random process $\mathcal{E}^\beta = \{E_t^\beta\}_{t=1,2,...}$, representing edge β, as the furthest point in the past of E_t^β which has influence on the future evolution of E_t^α:

$$\Omega(\mathcal{E}^\alpha||\mathcal{E}^\beta) = \Omega(\{E_t^\alpha\}_{t=1,2,...}||\{E_t^\beta\}_{t=1,2,...}) := \min_p \Big[p : \mathbb{P}(e_t^\alpha|e^\beta_{t-1,t-p}) = \mathbb{P}(e_t^\alpha|e^\beta_{t-1,-\infty})\Big], \tag{24}$$

where the subscript $e^\beta_{t-1,t-n}$ is shorthand for the sequence $e^\beta_{t-1}, e^\beta_{t-2}, ..., e^\beta_{t-n}$.
The memory co-order can be evaluated for each couple of links α and β, so that the whole memory shape of the temporal network can be fully characterised by the *co-memory matrix* $\mathbb{M}$, a $L \times L$ matrix with entries $\mathbb{M}_{\alpha\beta} = \Omega(\mathcal{E}^\alpha||\mathcal{E}^\beta)$, which accounts for the set of all possible co-orders. Notice that, by construction, the memory co-order of a link α with itself is precisely the memory order of the link, that is $\Omega(\mathcal{E}^\alpha||\mathcal{E}^\alpha) = \Omega(\{E_t^\alpha\}_t)$, so that matrix $\mathbb{M}$ contains in the diagonal entries information on the memory present in the evolution of each link considered as an independent dynamical process from the rest of the network.

Based on matrix $\mathbb{M}$ it is now possible to introduce another scalar projection of the network's memory, which we label *effective memory* $\Omega_{\text{eff}}(\mathcal{G})$, as the maximum value of the co-orders over the network link pairs:

$$\Omega_{\text{eff}}(\mathcal{G}) := \max_{\alpha,\beta} \left[\Omega(\mathcal{E}^\alpha||\mathcal{E}^\beta)\right] \tag{25}$$

It is easy to prove the following theorems, which relate the effective memory to the memory of the network as a whole.

Theorem 1. *Given a temporal network $\mathcal{G}$ with L edges and edge processes $\{\mathcal{E}^\alpha\}$ for $\alpha = 0, ..., L$, we have:*

$$\Omega(\mathcal{G}) \leq \max_{i,j}(\Omega(\mathcal{E}^\alpha||\mathcal{E}^\beta)) =: \Omega_{\textit{eff}}(\mathcal{G}), \tag{26}$$

where $\Omega(\mathcal{G})$ is the memory of the temporal network, $\Omega(\mathcal{E}^\alpha||\mathcal{E}^\beta)$ are the memory co-orders and $\Omega_{\textit{eff}}(\mathcal{G})$ is the effective memory.

Proof. It is useful to introduce the following compact notation: $E^{1,L}_{0,n} \equiv E^1_{0,n}, ..., E^L_{0,n}$ and $e^{1,L}_{0,n} = e^1_{0,n}, ..., e^L_{0,n}$. The n_{th} order conditional entropy $h_n(\mathcal{G}) = h_n(\{E_t^\alpha\})$ can be written as:

$$h_n(\mathcal{G}) = -\sum_{e^{1,L}_{0,n}} \mathbb{P}(e^{1,L}_{0,n}) \log \frac{\mathbb{P}(e^{1,L}_{0,n})}{\mathbb{P}(e^{1,L}_{1,n})}. \tag{27}$$

Expanding the joint probabilities in terms of conditional probabilities over the different links:

$$\mathbb{P}(e^{1,L}_{0,n}) = \prod_{\alpha=1}^{L} \mathbb{P}(e^\alpha_{0,n}|e^{1,\alpha-1}_{0,n}) \tag{28}$$

we can now write an expression for $h_n(\mathcal{G})$ in terms of the contributions from the different links:

$$\begin{aligned} h_n(\mathcal{G}) &= -\sum_{e^{1,L}_{0,n}} \prod_{\gamma=1}^{L} \mathbb{P}(e^{\gamma}_{0,n}|e^{1,\gamma-1}_{0,n}) \sum_{\alpha=1}^{L} \log \frac{\mathbb{P}(e^{\alpha}_{0,n}|e^{1,\alpha-1}_{0,n})}{\mathbb{P}(e^{\alpha}_{1,n}|e^{1,\alpha-1}_{1,n})} \\ &= -\sum_{\alpha=1}^{L} \sum_{e^{1,L}_{0,n}} \mathbb{P}(e^{\alpha}_{0,n}|e^{1,\alpha-1}_{0,n}) \log \frac{\mathbb{P}(e^{\alpha}_{0,n}|e^{1,\alpha-1}_{0,n})}{\mathbb{P}(e^{\alpha}_{1,n}|e^{1,\alpha-1}_{1,n})} \prod_{\gamma\neq\alpha} \mathbb{P}(e^{\gamma}_{0,n}|e^{1,\gamma-1}_{0,n}). \end{aligned} \tag{29}$$

We now note that the memory p must be consistent with equations 10 and 21 and hence must be the minimum value of n which maximises $h_n(\mathcal{G})$. Because of this we must have that any upper bound we find for the memory must also maximise $h_n(\mathcal{G})$. Hence, let us define our prospective effective memory p, which we will then show to be an upper bound, as:

$$p = \max_{\alpha,\beta} \left[\Omega(\mathcal{E}^{\alpha}\|\mathcal{E}^{\beta})\right]. \tag{30}$$

To test that this value of p is indeed a point for which we obtain the maximum value for our conditional entropy we take a value of $n \geq p$ so that $n = p + d$. The entropy is then given by

$$\begin{aligned} h_n(\mathcal{G}) = &-\sum_{\alpha=1}^{L} \sum_{e^{1,L}_{0,n}} \mathbb{P}(e^{\alpha}_{0}|e^{\alpha}_{1,p}, e^{1,\alpha-1}_{0,p}) \mathbb{P}(e^{\alpha}_{1,n}) \log \frac{\mathbb{P}(e^{1,\alpha}_{0}|e^{1,\alpha}_{1,p})}{\mathbb{P}(e^{1,\alpha-1}_{0}|e^{1,\alpha-1}_{1,p})} \prod_{\gamma\neq\alpha} \mathbb{P}(e^{\gamma}_{0}|e^{\gamma}_{1,p}, e^{1,\gamma-1}_{0,p}) \mathbb{P}(e^{\gamma}_{1,n}), \\ =& h_p(\mathcal{G}) \sum_{e^{1,L}_{p+1,p+d}} \mathbb{P}(e^{\alpha}_{p+1,p+d}) \prod_{\gamma\neq\alpha} \mathbb{P}(e^{\gamma}_{p+1,p+d}), \\ =& h_p(\mathcal{G}) \prod_{\alpha} \sum_{e^{\alpha}_{p+1,p+d}} \mathbb{P}(e^{\alpha}_{p+1,p+d}), \\ =& h_p(\mathcal{G}). \end{aligned} \tag{31}$$

This shows us that $h_n(\mathcal{G}) = h_p(\mathcal{G})$, and so $\Omega(\mathcal{G}) \leq p$. Since we defined p to be the maximum value of the possible co-orders of the network, and hence the effective memory, we have now proved that $\Omega(\mathcal{G}) \leq \max_{\alpha,\beta}\left[\Omega(\mathcal{E}^{\alpha}\|\mathcal{E}^{\beta})\right] =: \Omega_{\text{eff}}(\mathcal{G})$. That is to say, the memory of the temporal network is bounded above by the furthest time into the past of any link that has influence on the evolution of any other link. □

Corollary 1. *If for every pair of links α and β, with $\alpha \neq \beta$, the two stochastic processes $\mathcal{E}^{\alpha}$ and $\mathcal{E}^{\beta}$ are independent, i.e. $\forall\tau\, \mathbb{P}(e^{\alpha}_{1,\tau}, e^{\beta}_{1,\tau}) = \mathbb{P}(e^{\alpha}_{1,\tau})\mathbb{P}(e^{\beta}_{1,\tau})$, then*

$$\Omega(\mathcal{G}) = \Omega_{\mathit{eff}}(\mathcal{G}). \tag{32}$$

Proof. The proof can be obtained by looking at Eq. 29 directly. When links are independent, the conditional probabilities become $\mathbb{P}(e^{\alpha}_{0,n}|e^{1,\alpha-1}_{0,n}) = \mathbb{P}(e^{\alpha}_{0,n})$. This allows us to write:

$$h_n(\mathcal{G}) = \sum_{\alpha=1}^{L} h_n(\mathcal{E}^{\alpha}). \tag{33}$$

Clearly from this, combined with Eq. 12, we must have $\Omega(\mathcal{G}) = \max_{\alpha}\left[\Omega(\mathcal{E}^{\alpha})\right]$. But since links are independent, $\Omega(\mathcal{E}^{\alpha}\|\mathcal{E}^{\beta}) = \Omega(\mathcal{E}^{\alpha}\|\mathcal{E}^{\beta})\delta_{\alpha,\beta}$, and so:

$$\Omega(\mathcal{G}) = \max_{\alpha}\left[\mathcal{E}^{\alpha}\right] = \max_{\alpha,\beta}\left[\Omega(\mathcal{E}^{\alpha}\|\mathcal{E}^{\beta})\right] =: \Omega_{\text{eff}}(\mathcal{G}), \tag{34}$$

exactly as we aimed to prove. □

Corollary 2. *In the most general case of a temporal network $\mathcal{G}$, the memories of its links as given by the values $\Omega(\mathcal{E}^{\alpha})$, with $\alpha = 1, \ldots, L$, do not provide an upper or lower bound for $\Omega(\mathcal{G})$. That is to say, in general:*

$$\Omega(\mathcal{G}) \nleq \max_{\alpha}\left[\Omega(\mathcal{E}^{\alpha})\right], \tag{35}$$

$$\Omega(\mathcal{G}) \ngeq \max_{\alpha}\left[\Omega(\mathcal{E}^{\alpha})\right], \tag{36}$$

except in special cases, such as the one considered in the previous corollary.

Proof. It is sufficient to provide two examples: one in which the maximum memory of the links is less than the memory of the network, and one in which it is greater.
First we look at a case in which the maximum link memory is less than the network memory. Consider $\mathcal{G}$ formed by two links ruled by the stochastic processes $\{E_t^1\}$ and $\{E_t^2\}$. Now assume $E_t^2 \sim Bernoulli(y)$ and E_t^1 is drawn from a modified DAR(p) process so that $E_t^1 = Q_t E_{t-Z_t}^2 + (1 - Q_t)Y_t$, where $Q_t \sim Bernoulli(q)$, $Y_t \sim Bernoulli(y)$ (with the same value of y as E_t^2) and $Z_t \sim Uniform(1,p)$, for some values of q and p. Then $\Omega(\{E_t^1\}) = 0$ and $\Omega(\{E_t^2\}) = 0$, even though $\Omega(\mathcal{G}) = p$.
Second, we consider a case in which the maximum link memory is greater than the network memory. Consider again $\mathcal{G}$ with two links ruled by $\{E_t^1\}$ and $\{E_t^2\}$. Assume now $E_t^1 = Q_t E_{t-Z_t}^2 + (1-Q_t)Y_t$ and $E_t^2 = Q_t E_{t-Z_t}^1 + (1-Q_t)Y_t$, where $Q_t \sim Bernoulli(q)$, $Y_t \sim Bernoulli(y)$. As in the previous case the memory of the network is p. however, if we substitute our expression for E_t^2 into our formula for E_t^1, then we obtain $E_t^1 = Q_t^1(Q_t^2 E_{t-(Z_t^1+Z_t^2)}^1 + (1-Q_t^2)Y_t^2) + (1-Q_t^1)Y_t^1$. Clearly then $\Omega(\{E_t^1\}) = 2p$, which is greater than $\Omega(\mathcal{G})$. We will further explore the reasoning for this in sections III and IV. □

Conceptually speaking, observe that the effective memory is similar to Granger causality in that it considers the influence of past states of a process on the present evolution of another process [9]. However, they are not the same. Indeed, in the toy models that we introduce later on to demonstrate the cases where the scalar memory $\Omega(\mathcal{G})$ does not capture the influence of memory on spreading processes, it can also be seen that Granger causality suffers from the same issue, as here the extent of any causality between the two links is precisely the scalar memory.

C. Estimating the co-memory matrix $\mathbb{M}$

We have shown that the memory of a temporal network can be understood in terms of the co-orders of its links, which represent the memory that one link has of another. As in the main text, we define the co-order $\Omega(\mathcal{X}||\mathcal{Y})$ of a link process $\mathcal{X}$ composed of random variables X_t with realisations x_t, with respect to link process $\mathcal{Y}$ composed of random variables Y_t with realisations y_t, as

$$\Omega(\mathcal{X}||\mathcal{Y}) = \min_p \left[p : \mathbb{P}(x_t|y_{t-1,t-p}) = \mathbb{P}(x_t|y_{t-1,-\infty})\right]. \tag{37}$$

What remains to be found however is a way of estimating this value. We will here adapt the form of the efficient determination criterion (EDC) given in Eq. 17 and 20. Firstly, it is clear that both the number of states m and the number of observations T are consistent with their applications to sequences in general. Specifically m will be 2 (since links are either present or not) and T is defined by the data being used. This means that we now need only focus our attention on the log-likelihood function $\log L_k$. Again, given a pair of sequences $\{X_t\}$ and $\{Y_t\}$ with realisations x_t and y_t respectively, and where $t = 0, ..., T$, the likelihood of observing the full sequence X_t given the last k values of Y_t, denoted by $L(X_t|Y_{t-1,t-k})$ is given by

$$\begin{aligned} L(X_t|Y_{t-1,t-k}) &= \prod_{i=0}^{T} \mathbb{P}(x_i|y_{i-1,i-k}), \\ &= \prod_{x_i} \prod_{y_{i-1,i-k}} \mathbb{P}(x_i|y_{i-1,i-k})^{n(x_i,y_{i-1,i-k})}. \end{aligned} \tag{38}$$

Where, similarly to before, $y_{i-1,i-k}$ is the joint $y_{i-1}, y_{i-2}, ...y_{i-k}$, and the counting function $n(x_i, y_{i-1,i-k})$ is defined by

$$n(x_i, y_{i-1,i-k}) = \sum_{i=k+1}^{T} I(X_i = x_i, Y_{i-1} = y_{i-1}, ..., Y_{i-k} = y_{i-k}). \tag{39}$$

Taking the empirical estimate for the conditional

$$\mathbb{P}(x_i|y_{i-1,i-k}) \approx \frac{n(x_i, y_{i-1,i-k})}{n(y_{i-1,i-k})}, \tag{40}$$

we can then write the log-likelihood as

$$\log L_k = \sum_{x_i} \sum_{y_{i-1,i-k}} n(x_i, y_{i-1,i-k}) \log \frac{n(x_i, y_{i-1,i-k})}{n(y_{i-1,i-k})}. \tag{41}$$

Now, precisely as before, we obtain the estimator

$$\mathrm{EDC}(k) = -2\log L_k + 2m^{k+1}\log\log T, \tag{42}$$

giving co-order estimate

$$p_{\mathrm{EDC}} = \underset{0\le k\le K}{\arg\min}\,\mathrm{EDC}(k). \tag{43}$$

An example where we show explicitly the value taken by the estimator $\mathrm{EDC}(k)$ for a concrete co-order $\Omega(\mathcal{E}^1\|\mathcal{E}^1)$ is depicted in Fig.S4. In that figure we also plot the autocorrelation function of the signal $\{E^1_t\}$, defined in the usual way $\mathrm{ACF}(\tau)\propto\langle E^1_t\cdot E^1_{t+\tau}\rangle_t$.

D. Pair Memory $\Omega_{\mathbf{pair}}(\mathcal{G})$

Here we explore an alternative approach to estimating the scalar memory of a temporal network. We do this by considering pairs of links as an indivisible object, and thus proceed to analyse the memory of the pair as if it were its own temporal network. By this we mean that, if we had two links $\mathcal{E}^\alpha$ and $\mathcal{E}^\beta$ ($\alpha\ne\beta$) then rather than looking at the set $\{\Omega(\mathcal{E}^\alpha),\Omega(\mathcal{E}^\beta),\Omega\left(\mathcal{E}^\alpha\|\mathcal{E}^\beta\right),\Omega\left(\mathcal{E}^\beta\|\mathcal{E}^\alpha\right)\}$, we look at the order of the random vector directly: $\Omega\left((\mathcal{E}^\alpha,\mathcal{E}^\beta)\right)$.

An advantage to this alternative approach is that, while it is possible to measure the co-orders of pairs of links directly, this does not immediately allow us to make use of the great body of work that has been done on the estimation of the memory of a general symbolic sequence. We also show that in general this is a better estimate of the scalar memory than the effective memory.

From 26 we know that the memory of a temporal network $\mathcal{G}$ with generating edge processes $\mathcal{E}^\alpha$ is bounded above by

$$\Omega(\mathcal{G})\le\max_{\alpha,\beta}\left(\Omega(\mathcal{E}^\alpha\|\mathcal{E}^\beta)\right)=\max_{\alpha,\beta}\left[\mathbb{M}_{\alpha\beta}\right]=:\Omega_{\mathrm{eff}}(\mathcal{G}). \tag{44}$$

Now, consider two links from this network: $\mathcal{E}^1$ and $\mathcal{E}^2$. In isolation they form their own temporal network $\mathcal{G}^{1,2}$ whose scalar memory is bounded above by $\max\{\Omega(\mathcal{E}^1),\Omega(\mathcal{E}^2),\Omega\left(\mathcal{E}^1\|\mathcal{E}^2\right),\Omega\left(\mathcal{E}^2\|\mathcal{E}^1\right)\}$. Since this new temporal network contains only two links, it can only exist in 4 possible states $(e^1_t,e^2_t)\in\{(0,0),(0,1),(1,0),(1,1)\}$. This is the alphabet from which we sample the dynamics. This is not an unreasonably large state space, and so we can estimate the memory directly. All that remains to do is index this state space. To do this let us first detail a more general concept: given two time series $\{X_t\},\{Y_t\}\in\{0,1\}$ with realisations x_t and y_t respectively, define the product time series $\{Z_t\}\in\{0,3\}$ with realisations z_t, as $z_t=f(x_t,y_t)$ where $f:\{0,1\}\times\{0,1\}\to[0,3]$ is any bijection (for example $z_t=x_t+2y_t$). Let us denote $Z_t=f(X_t,Y_t)\ \forall t$. Let us now take $Z_t=f(E^1_t,E^2_t)$. We know that, since f is a bijection, the possible states of Z_t are simply labels for the possible states of (e^1_t,e^2_t). As such $\Omega(Z_t)=\Omega(\mathcal{G}^{1,2})$, and hence we can get the memory of the two link sub-network directly. What remains to be seen is how this translates to the memory of the temporal network as a whole.

If we now consider each possible pair of links E^i_t,E^j_t, and their product sequence $Z^{ij}_t=f(E^i_t,E^j_t)$, then we can obtain the following result:

Theorem 2. *For a temporal network $\mathcal{G}$ with link processes $\mathcal{E}^\alpha$ and where the product of pairs $Z^{\alpha\beta}_t=f(E^\alpha_t,E^\beta_t)$ are given by some bijection $f:\{0,1\}\times\{0,1\}\to[0,3]$, where the effective memory is given by $\Omega_{\mathit{eff}}(\mathcal{G})=\max_{\alpha,\beta}\left[\Omega(\mathcal{E}^\alpha\|\mathcal{E}^\beta)\right]$, and the pair memory is given by $\Omega_{\mathit{pair}}(\mathcal{G})=\max_{\alpha,\beta}\left[\Omega(\{Z^{\alpha\beta}_t\})\right]$, the following inequality holds:*

$$\Omega(\mathcal{G})\le\Omega_{pair}(\mathcal{G})\le\Omega_{\mathit{eff}}(\mathcal{G}). \tag{45}$$

Proof. Let us prove the second of these inequalities first: $\Omega_{\mathrm{pair}}(\mathcal{G})\le\Omega_{\mathrm{eff}}(\mathcal{G})$. We know that for any pair of links (α,β), $\Omega(\{Z^{\alpha\beta}_t\})\le\max\{\Omega(\mathcal{E}^\alpha),\Omega(\mathcal{E}^\beta),\Omega(\mathcal{E}^\alpha\|\mathcal{E}^\beta),\Omega(\mathcal{E}^\beta\|\mathcal{E}^\alpha)\}$. Let us assume that $\Omega(\{Z^{\alpha\beta}_t\})$ is maximal for the links $(\alpha,\beta)=(\alpha',\beta')$, then

$$\begin{aligned}\max_{\alpha,\beta}\left(\Omega(\{Z^{\alpha\beta}_t\})\right)&=\Omega(\{Z^{\alpha'\beta'}_t\})\\&\le\max\{\Omega(\mathcal{E}^{\alpha'}),\Omega(\mathcal{E}^{\beta'}),\Omega\left(\mathcal{E}^{\alpha'}\|\mathcal{E}^{\beta'}\right),\Omega\left(\mathcal{E}^{\beta'}\|\mathcal{E}^{\alpha'}\right)\}\\&\le\max_{\alpha,\beta}\left[\Omega(\mathcal{E}^\alpha\|\mathcal{E}^\beta)\right].\end{aligned} \tag{46}$$

As required.
Now the first of the inequalities. Assume without loss of generality that $\Omega(\mathcal{G}) = p$. Then there must exist at least one link $\mathcal{E}^m$ with $p = \min_n(n : \mathbb{P}(e_t^m | e_{t-1,t-n}^{1,L}) = \mathbb{P}(e_t^m | e_{t-1,-\infty}^{1,L}))$, i.e. there must be at least one link ℓ which remembers some part of the network p time steps ago. We then see that there is at least one link $\mathcal{E}^\ell$ which is remembered at least p time steps ago, i.e. $\mathbb{P}(e_t^m | e_{t-1,t-p}^{1,L})$ is a function of $\mathcal{E}^\ell$ (and other links and time indices). Taking the pair process $\{Z_t^{m\ell}\}$ we must hence have that $\Omega(\{Z_t^{m\ell}\}) \geq p$, since its conditional must be a function containing terms at least p steps into the past. Hence there exists some (α, β) such that $\Omega(\{Z_t^{\alpha\beta}\}) \geq \Omega(\mathcal{G})$, and hence we must have that $\Omega(\mathcal{G}) \leq \Omega_{pair}(\mathcal{G})$. This concludes our proof. □

Notably, we must also have that $\Omega(\{Z_t^{\alpha\beta}\}) = \Omega(\{Z_t^{\beta\alpha}\})$, and so for a network with L links only $L(L-1)/2$ pairs of values (α, β) must be checked to find the maximum, and hence the estimated pair memory of the network, meaning that this approach may be faster to implement than finding the co-orders directly in some cases (though, possibly because of the choice of estimator used in this work, this is not the case here).
Finally, observe that, according to the notation used in the main manuscript to allude to the spectrum of memories $\Omega_m(\mathcal{G})$, we have

$$\Omega_{\text{pair}}(\mathcal{G}) = \Omega_2(\mathcal{G}).$$

In the following sections we provide detailed explanations on why neither $\Omega(\mathcal{G})$ nor $\Omega_{\text{pair}}(\mathcal{G})$ are actually well-defined to accurately describe the scalar memory of a temporal network, and if a scalar memory was needed, then $\Omega_{\text{eff}}(\mathcal{G})$ is the quantity to choose.

III. WHY THE CO-MEMORY MATRIX $\mathbb{M}$ AND THE EFFECTIVE MEMORY $\Omega_{\text{eff}}(\mathcal{G})$ ARE BETTER DEFINED AND MORE USEFUL CONCEPTS THAN THE SCALAR MEMORY $\Omega(\mathcal{G})$

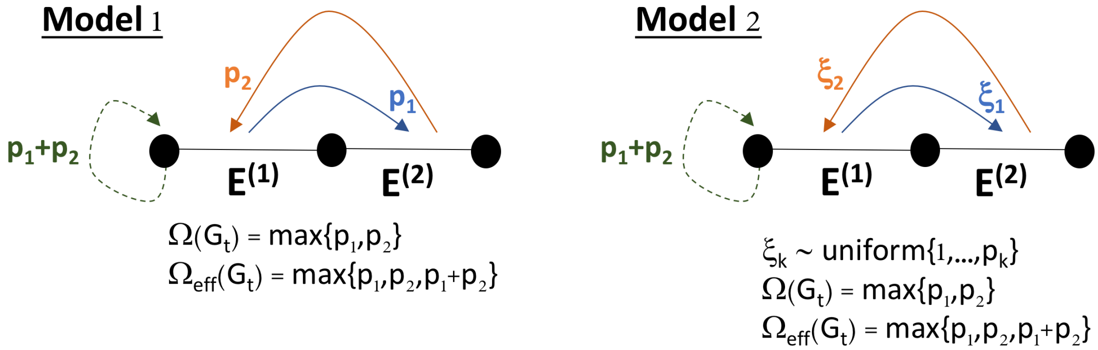

FIG. S1. Models of temporal networks with two links and tunable memories. In both models the temporal activity of each link depends on the past of the other link. However the memory kernel is different for the two models. In *model 1*, link 1 can copy the $t - p_1$ state of link 2, while link 2 can copy the $t - p_2$ state of link 1, thereby inducing a virtual loop of memory $p_1 + p_2$ in the dynamics of each link. In *model 2*, the first link can copy a state of the second link, choosing uniformly at random from the previous $t-1, ..., t-p_1$ available states. Analogouly the second link can copy one of the $t-1, ..., t-p_2$ states of the first link. This model also induces virtual loops of memory $p_1 + p_2$, although, in practice the estimation method may not necessarily be able to identify these virtual loops due to virtual loop decoherence (see text for details).

A. Two toy models and their virtual loops

The main reason why it is important to properly define and extract the memory of a temporal network is to unveil its influence on the dynamics of processes occurring over the network. We will show here that the co-orders $\mathbb{M}_{\alpha\beta} = \Omega(\mathcal{E}^\alpha \| \mathcal{E}^\beta)$ and the associated effective memory $\Omega_{\text{eff}}(\mathcal{G})$ of a temporal network $\mathcal{G}$ are as quantities, better suited to characterising spreading processes occurring over $\mathcal{G}$ than traditional definitions such as $\Omega(\mathcal{G})$, which is not only computationally difficult to estimate, but also suffers from fundamental drawbacks. We will illustrate this by means

of the following two temporal network models.

Model 1 – The first temporal network we consider is a chain graph with three nodes and two links with binary states 0, 1. The temporal activity of the two links is ruled by the two coupled stochastic processes $\mathcal{E}^1 = \{E_t^1\}_{t=1,2,...}$ and $\mathcal{E}^2 = \{E_t^2\}_{t=1,2,...}$ defined as:

$$E_t^1 = Q_t^1 E_{t-p_1}^2 + (1 - Q_t^1) Y_t^1, \tag{47}$$

$$E_t^2 = Q_t^2 E_{t-p_2}^1 + (1 - Q_t^2) Y_t^2, \tag{48}$$

with $Q_t^1, Q_t^2 \sim Bernoulli(q)$, $Y_t^1, Y_t^2 \sim Bernoulli(y)$ and p_1 and p_2 two positive integers. This means that, at each time step t, each link will either be sampled from a Bernoulli trial, or it will copy from the past states of the other link. In the latter case, link 1 will copy the state of link 2 at time $t - p_1$, i.e. exactly p_1 steps back in the past, while link 2 will copy the state of link 1 at time $t - p_2$. The model is illustrated in the left panel of Figure S1.

We can now state and prove the following theorem on the memory $\Omega(\mathcal{G})$ of model 1:

Theorem 3. *If a temporal network $\mathcal{G}$ is determined by model 1 above, then* $\Omega(\mathcal{G}) = \max\{p_1, p_2\}$

Proof. By construction we have that $\mathbb{P}(e_t^{1,2} | e_{t-1,t-p}^{1,2}) = \mathbb{P}(e_t^1 | e_{t-1,t-p}^{1,2}) \mathbb{P}(e_t^2 | e_{t-1,t-p}^{1,2})$. Note that $e_{t-1,t-p}^{1,2} = e_{t-1,t-p}^1, e_{t-1,t-p}^2$. Then the conditional probability that link 1 is present is given by

$$\mathbb{P}(e_t^1 = 1 | e_{t-1,t-p_1}^{1,2}) = q e_{t-p_1}^2 + (1-q) y, \tag{49}$$

and similarly for E_t^2. It is then clear that for any $\delta > 0$

$$\begin{aligned} \mathbb{P}(e_t^\alpha | e_{t-1,t-p_1}^{1,2}) &= \mathbb{P}(e_t^\alpha | e_{t-1,t-(p_1+\delta)}^{1,2}), \\ \mathbb{P}(e_t^\alpha | e_{t-1,t-p_1}^{1,2}) &\neq \mathbb{P}(e_t^\alpha | e_{t-1,t-(p_1-\delta)}^{1,2}), \end{aligned} \tag{50}$$

for $\alpha = 1, 2$. Hence we must have that $\min_p \left[p : \mathbb{P}(e_t^{1,2} | e_{t-1,t-p}^{1,2}) = \mathbb{P}(e_t^{1,2} | e_{t-1,t-\infty}^{1,2}) \right] = \max(p_1, p_2)$. □

Interestingly, when we look at the different entries of the matrix $\mathbb{M}$, we get some intriguing results. In fact, in this case the matrix is two-dimensional and, together with the terms $\Omega(\mathcal{E}^1 \| \mathcal{E}^2) = p_1$ and $\Omega(\mathcal{E}^2 \| \mathcal{E}^1) = p_2$ we must also evaluate the diagonal terms $\Omega(\mathcal{E}^1 \| \mathcal{E}^1)$ and $\Omega(\mathcal{E}^2 \| \mathcal{E}^2)$.
Let us first consider $\Omega(\mathcal{E}^1 \| \mathcal{E}^1)$. We can treat the coupled system in Eq. 48 by re-writing an expression for E_t^1 containing only terms related to link 1 directly. We obtain:

$$E_t^1 = Q_t^1 (Q_t^2 E_{t-(p_1+p_2)}^1 + (1 - Q_t^2) Y_t^2) + (1 - Q_t^1) Y_t^1. \tag{51}$$

which clearly shows that $\Omega(\mathcal{E}^1 \| \mathcal{E}^1) = p_1 + p_2$. Similarly we can prove that $\Omega(\mathcal{E}^2 \| \mathcal{E}^2) = p_1 + p_2$. These results have been confirmed by numerically simulating the model and measuring the co-orders directly. By construction, this means that the effective memory of this system is $\Omega_{\text{eff}}(\mathcal{G}) = p_1 + p_2$, which is possibly up to twice the value of the scalar memory $\Omega(\mathcal{G})$. If, without any loss of generality, we set $p_1 > p_2$ and then fix p_1 and let p_2 vary, we can thereby construct a variety of temporal networks with exactly the same scalar memory $\Omega(\mathcal{G}) = p_1$, but with different co-memory matrices and a tunable effective memory $\Omega_{\text{eff}}(\mathcal{G}) = p_1 + p_2$. In this case, the difference between $\Omega_{\text{eff}}(\mathcal{G})$ and $\Omega(\mathcal{G})$ is the result of an induced *virtual loop* (VL) in the dynamics of the temporal network: when link 1 draws from the memory of link 2, it may effectively be drawing from its own, more distant, past. Importantly, these contributions to the memory of the network are intrinsically indirect: the source of memory present microscopically at link E_t^1 and E_t^2 is induced by the coupling of the link activities. Of course, these effects are obtained as we restrict the observation state space to single links. These mechanisms are similar to what happens when we project a low-order Markov chain defined on a given state space onto a smaller dimensional state space. While these effects can be seen as an artefact of such a projection, they turn out to have important consequences on dynamical processes taking place on temporal networks, as we will show in the next section. But first let us introduce a more nuanced model of a network with two links, in which the virtual loops have a different structure.

Model 2 – In order to study the effects of virtual loops in a slightly more realistic –but still controlled– setting, we introduce a second toy model in which, given the same values of p_1 and p_2 from our first model, we obtain exactly the same scalar memory $\Omega(\mathcal{G})$, but with a different memory kernel. Rather than the memory sampling from some fixed number of time steps into the past, we now sample uniformly among the past p_1 or p_2 states. The link evolution of

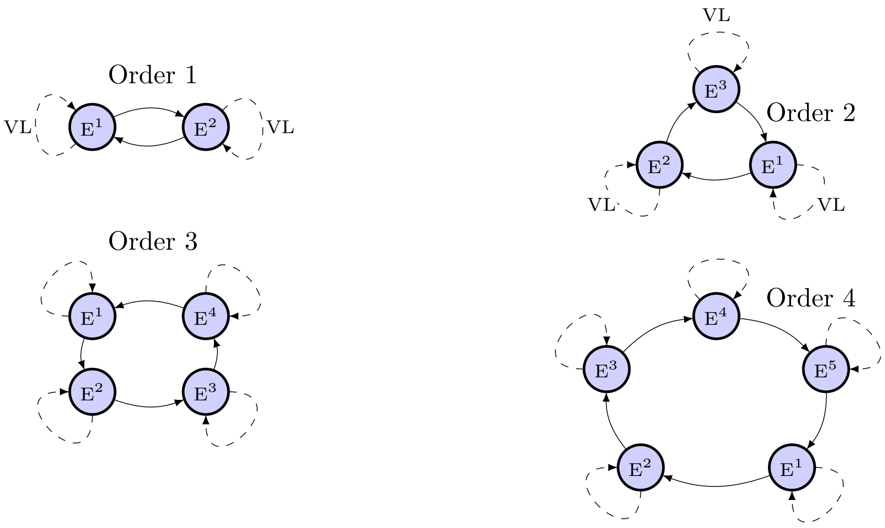

FIG. S2. Examples of cyclic Bayesian networks describing the link dependencies from which virtual loops emerge. Virtual loops produce long memory contributions in the diagonal entries of the co-memory matrix. These loops are induced by appropriately coupling the dynamics of the links. Shown are possible configurations where VLs of order 1, 2, 3 and 4 can emerge. The nodes in each diagram denote the stochastic process $\mathcal{E}^\alpha$ associated to link $\alpha = 1, 2, \ldots, L$ of a temporal network. Solid arrows denote temporal dependencies between links, whereas dashed arrows indicated induced VLs.

this model is illustrated in the right panel of Figure S1 and is specified by a coupled pair of slightly modified DAR(p) processes:

$$E_t^1 = Q_t^1 E^2_{t-Z_t^1} + (1 - Q_t^1) Y_t^1, \tag{52}$$

$$E_t^2 = Q_t^2 E^1_{t-Z_t^2} + (1 - Q_t^2) Y_t^2, \tag{53}$$

where $Q_t^1, Q_t^2 \sim Bernoulli(q)$, $Y_t^1, Y_t^2 \sim Bernoulli(y)$, and $Z_t^1 \sim Uniform(1, p_1)$ and $Z_t^2 \sim Uniform(1, p_2)$. As in model 1, at each time step, each link in model 2 will either be sampled from a Bernoulli trial, or it will copy from the past states of the other link. However, the first link will not exactly copy the state of link 2 at time $t - p_1$, but it will copy one of the previous $t-1, ..., t-p_1$ states of the second link selected at random with uniform probability. The scalar memory of model 2 is clearly $\Omega(\mathcal{G}) = \max\{p_1, p_2\}$, as this is the furthest point into the past of the network that is required to when generating the next step of its evolution. A formal proof of this will be given in theorem 5, as this model is a specific case of the eCDARN(p) model described in section IVA. Again, as in the previous model we find interesting patterns when we look at the different elements of the co-memory matrix $\mathbb{M}$. As in model 1 we have $\Omega(\mathcal{E}^1 \| \mathcal{E}^2) = p_1$ and $\Omega(\mathcal{E}^2 \| \mathcal{E}^1) = p_2$. To evaluate $\Omega(\mathcal{E}^1 \| \mathcal{E}^1)$ we rewrite E_t^1 using only terms related to link 1, obtaining:

$$E_t^1 = Q_t^1 (Q_t^2 E^1_{t-(Z_t^1 + Z_t^2)} + (1 - Q_t^2) Y_t^2) + (1 - Q_t^1) Y_t^1. \tag{54}$$

From this we can then see that the dynamics of the first link can equivalently be described by the following process:

$$E_t^1 = \bar{Q}_t E^1_{t-(Z_t^1 + Z_t^2)} + \left(1 - \bar{Q}_t\right) Y_t, \tag{55}$$

where $\bar{Q}_t \sim Bernoulli(q^2)$ and $Y_t \sim Bernoulli(y)$. Hence the stochastic process $\{E_t^1\}_{t=1,2,\ldots}$ is a form of DAR(p) process of order $p_1 + p_2$ [10]. Implicitly, this tells us that $\Omega(\mathcal{E}^1 \| \mathcal{E}^1) = p_1 + p_2$, and analogously we can see that $\Omega(\mathcal{E}^2 \| \mathcal{E}^2) = p_1 + p_2$. Again these results are confirmed by measuring the co-orders directly through numerical simulations of the model. Summing up, the effective memory of this system is, as expected $\Omega_{\text{eff}}(\mathcal{G}) = p_1 + p_2$. Note that if $p_1 = p_2$ then $\Omega_{\text{eff}}(\mathcal{G}) = 2\Omega(\mathcal{G})$.

In exactly the same way as before, and without loss of generality we can fix $p_1 > p_2$ and let p_2 vary to construct a variety of temporal networks with exactly the same scalar memory $\Omega(\mathcal{G}) = p_1$, but with different co-memory matrices and tunable effective memories $\Omega_{\text{eff}}(\mathcal{G})$.

B. Virtual loops of arbitrary order and conditions for VLs to emerge

The temporal network toy models discussed above consist of a linear chain of three nodes and two links. As illustrated in figure S2, a convenient way of representing these temporal networks is by a graphical model with two nodes, describing the two links (stochastic processes) of the temporal networks, connected by directed arrows that express the temporal dependence structure between different network links. This representation is known in the literature as a Bayesian network (BN), and when links indeed describe causal relationships, it is often termed as a Bayesian or causal network. While BNs are usually directed acyclic graphs (DAGs) by definition, it is easy to see that the BN associated to our toy temporal network models is indeed cyclic (CBN), and this is indeed a sufficient condition for virtual loop effects to emerge in memory.

Models 1 and 2 are among the simplest temporal networks to provide a nontrivial virtual loop (VL) structure. Because VLs in this case are induced via a causal path involving only a pair of links ($E^1 \rightarrow E^2 \rightarrow E^1 \Rightarrow E^1 \circlearrowleft$), we denote this as a VL of order 1. Now, it is easy to construct 'higher order' virtual loops e.g. simply by building temporal networks with an underlying Bayesian ring topology where link 1 dynamically depends on link 2, link 2 dynamically depends on link 3, etc., and link n dynamically depends on link 1. CBNs with VLs of orders 2, 3 and 4 are shown in figure S2 together with one of order 1. In theory, VLs of higher order induce contributions to the co-memory matrix with longer memory, yielding a longer effective memory $\Omega_{\text{eff}}(\mathcal{G})$. In practice, virtual loops with high memory are difficult to observe due to extremely long time series being required to capture the effect. More importantly, these VLs are stable as long as the temporal dependencies between links are fine tuned, and quickly dissipate otherwise, meaning that the practical relevance of VLs in the context of real-world temporal networks is less clear. In the next subsection, by comparing models 1 and 2 in more detail, we will give a closer look to this mechanism of "virtual loop decoherence".

Before that, we should also highlight that the underlying BN needs to not be cyclic for virtual loops to emerge: this is a sufficient, not necessary condition. Indeed, when links are also auto-correlated (meaning that on top of the link temporal dependencies, we prescribe that the link has also an internal dynamics which is auto-correlated), then virtual loops can also emerge even in the case that the underlying BN is a priori acyclic. The reason is because in that case, the interplay between the auto and cross-correlated dynamics induce virtual Bayesian links. This can be better explained with an example. Suppose that link α has an internal auto-correlated activity, and on top of that, also depends on the past of link β. The interplay between the two dynamics is captured in the auto-correlated nature of α, which displays signs of memory which are a mix of the ones obtained from its own internal dynamics and from the memory displayed by β, hence the actual memory of α (as measured by the co-order $\Omega(\mathcal{E}^\alpha||\mathcal{E}^\alpha)$) is in general larger than the memory of the internal dynamics of α. This effect is indistinguishable from a virtual loop, hence we can confidently label it in a similar way.
Accordingly, the following table summarises when virtual loops emerge in the system.

**Autocorrelated (internal dynamics)**	**Cross-correlated (link dependency)**	**Virtual loops**
NO	NO	NO
NO	YES, ACYCLIC BN	NO
NO	YES, CYCLIC BN	YES
YES	NO	NO
YES	YES, ACYCLIC BN	YES
YES	YES, CYCLIC BN	YES

TABLE I. Summary of the situations where virtual loops are expected to emerge.

C. Virtual loops in other areas of physics and beyond

The virtual loops discussed above are indeed a particular case of "causal loops", and therefore share some similarities with important concepts arising in different areas of physics, computer science and engineering. For instance, when finding the marginal distributions of a collection of random variables it is common to use the message passing, or belief propagation, algorithm [11, 12]. Such methods are important in the study of Gaussian graphical models in machine learning [13], signal processing [14], and a plethora of other such inference problems [15–17]. These message passing algorithms also have uses in the context of statistical physics, where they can be related to the Bethe-Peierls approximation (or the replica symmetric cavity method in the context of spin glasses), and in turn the Thouless, Anderson, Palmer equations for local magnetisations [18–21]. However, this approach becomes inexact precisely when

the Bayesian graphs that underly these problems have loops. Because of this the study of how to best overcome this problem has been seen as deeply important [22–24], and indeed has been a subject of recent attention [25]. In the same vein, causal loops have been a subject of interest in the study of Feynman diagrams. "One-loop" diagrams, in which there is a single causal loop, have historically presented challenges to study [26, 27]. However when these challenges have been overcome they have helped to explain phenomena such as the Casimir effect [28, 29], Hawking radiation [30] and the Lamb shift [31].

D. The phenomenon of virtual loop decoherence

The difference between the two toy models lies in the ways in which the state of the system depends on the past states. Even though in theory the scalar memory $\Omega(\mathcal{G})$, the effective memory $\Omega_{\text{eff}}(\mathcal{G})$ and the co-memory matrices are identical in the two models, in practice the estimation of these quantities is different. In our first model, the extent of the memory is localised: each link will, when referencing from the past, always look at the state of the other link a fixed number of time steps into the past (p_1 or p_2). In our second model each link will, when referencing from the past, uniformly pick from among the past p_1 (or p_2) states of the other link. In other words, links in the second model will seldom copy the past state exactly p_1 or p_2 time steps ago. While theoretically the local co-memory of each link is still $p_1 + p_2$ due to the presence of virtual loops, whether we can accurately estimate this quantity is less obvious. In theory we would require a very large observed time series to estimate the theoretical effective memory $p_1 + p_2$ consistently. While this is a finite size effect, it is however important with regards to processes running on top of these networks, and therefore will affect e.g. the behaviour of spreading processes we run on them, as we will show in the next section. It is therefore in this second model that we would expect to observe what we term "virtual loop decoherence": each link will not always utilise the full extent of its potential memory, in that the virtual loops will not always reference a point at time $t-(p_1+p_2)$ in their past history. Because of this we expect the influence of the virtual loops to be limited in comparison to our first toy model, and hence the theoretical co-order $\Omega(\mathcal{E}_t^1||\mathcal{E}_t^1) = p_1 + p_2$ will be difficult to detect. The effect of this is that the estimated values of the diagonal terms in the co-memory matrix will in some cases be smaller than the effective memory of the network, or in more extreme cases, less than $\Omega(\mathcal{G})$. In either of these cases virtual loops will not contribute to $\Omega_{\text{eff}}(\mathcal{G})$ (virtual loop decoherence). As a byproduct, decoherence would cause the estimation of $\Omega_{\text{eff}}(\mathcal{G})$ to approach the scalar memory $\Omega(\mathcal{G})$, meaning that in those practical scenarios where VL decoherence does emerge, then the scalar memory $\Omega(\mathcal{G})$ might after all be a good approximation to the effective memory operating underneath. In other words, conceptually the correct scalar quantity under study is $\Omega_{\text{eff}}(\mathcal{G})$ and not $\Omega(\mathcal{G})$, but when the system is free from VLs or these decohere, then both quantities tend to be close.

To analyse this we generate 10^4 instances of both models with randomly selected values of p_1 and p_2 and a range of values for the memory strength q and the link probability y, along with the number of time steps each network is generated for. For each instance we estimate the effective memory $\Omega_{\text{eff}}(\mathcal{G})$, and record a "hit" if this estimate is precisely $p_1 + p_2$. We plot in Fig. S3 this hit rate, as a function of the parameters q and y. As expected, we see that in all but the few cases where the memory in each model is removed ($q \approx 0$), that the hit rate for our first model is markedly higher than for the second. In particular, the hit rate of model 1 is consistently 100% for a large range of the model parameters. On the other hand, the hit rate for model 2 is typically smaller, hovering around 50% for the same parameter range, suggesting that the virtual loops which are present only cause the estimated co-memory to be $p_1 + p_2$ in at most half of the sampled cases, while for the rest these loops are decoherent. This effectively causes the estimation of $\Omega_{\text{eff}}(\mathcal{G})$ to approach the scalar memory $\Omega(\mathcal{G}) = p_1$.

To further complement this analysis, we have computed, for each temporal network realisation of models 1 and 2, the normalised frequency histogram of the dispersion $d = [\Omega_{\text{eff}}(\mathcal{G}) - (p_1 + p_2)]/p_2$. For a given set of realisations of each temporal network model, d accounts for how well the estimated effective memory approximates the theoretical one ($p_1 + p_2$, induced by the virtual loop), normalized over p_2. Finding $d = 0$ means that the estimation matches the theory and virtual loops govern the effective memory. For $d \neq 0$, virtual loop decoherence sets in. Typically, we expect that in this (and indeed most) scenarios we will observe $d < 0$, meaning that the estimated memory contribution of any virtual loops is smaller than $p_1 + p_2$. When this contribution gets smaller, d approaches its minimum value $d = -1$, the case associated with virtual loops being completely decoherent and the effective memory coinciding with the scalar memory $\Omega(\mathcal{G}) = p_1$. In the right panel of Figure S3 we depict the histograms of d for an ensemble of 10^4 realisations of models 1 and 2, with parameters $q = 0.9$, $y = 0.25$ and with p_1 and p_2 samples uniformly randomly from the range $1, .., 5$ (inclusive). We systematically find $d = 0$ for model 1, as expected given that the hit rate is 100% for this model. In the case of model 2, we find that the histogram is more scattered, favouring situations with $d < 0$. This means that the memory contribution to the effective memory of the virtual loops is decreased, and accordingly $\Omega_{\text{eff}}(\mathcal{G})$ gets closer to $\Omega(\mathcal{G})$, although this trend is never reached as VLs never completely decohere (see however theorem 6 for a rigorous

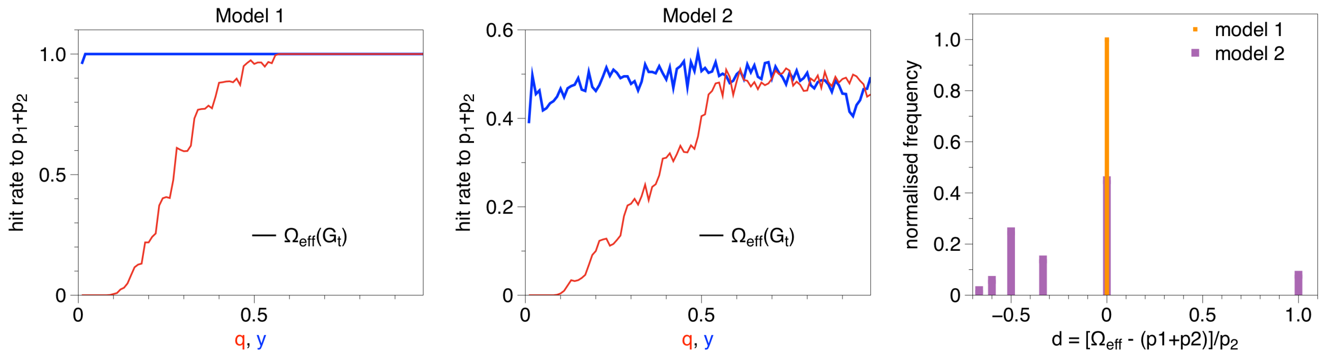

FIG. S3. **Hit rates and hisrograms for both toy models.** (Left and middle panels) Hit rate measuring the fraction of sampled toy model networks for which the estimation of $\Omega_{\text{eff}}(\mathcal{G})$ coincides with the local co-memory induced by the virtual loops, as given by $\Omega(E^1_t \| E^1_t) = p_1 + p_2$, for model 1 (left panel) and model 2 (middle panel). For each realisation of the temporal network, we sample p_1, p_2 uniformly from a range $1, 2, \dots, 10$. Both models depend on parameters q (memory length) and y (link probability, see the text for an explanation of these parameters). The red curve displays the dependence of the hit rate on q for a fixed $y = 0.25$, whereas blue curve displays the dependence on y for a fixed $q = 0.9$. In every case we let the network evolve for $T = 10^5$ time steps, and every point is the average of 10^4 realisations (each realisation having a different p_1, p_2). In model 1 the virtual loops govern $\Omega_{\text{eff}}(\mathcal{G})$, whereas in model 2 virtual loop decoherence sets in and the estimated $\Omega_{\text{eff}}(\mathcal{G})$ is no longer systematically governed by the virtual loops. (Right panel) Dispersion histogram where we count the frequency of each estimated $\Omega_{\text{eff}}(\mathcal{G})$ (averaged over 10^4 realisations of the temporal network). We compare this quantity to $p_1 + p_2$ by measuring the normalised dispersion $d = [\Omega_{\text{eff}}(\mathcal{G}) - (p_1 + p_2)]/2$ for models 1 and 2, with fixed parameters $q = 0.9$ and $y = 0.25$. In model 1 the dispersion is systematically zero (a result of a 100% hit rate), i.e. virtual loops systematically govern the estimation of the effective memory. In model 2 virtual loops are decoherent and the estimated value of the effective memory tends to be smaller than $p_1 + p_2$ accordingly. We do not however reach the asymptotic case $d = -1$ that that we associate with the effective memory matching the scalar memory, and hence virtual loops not contributing.

proof that full virtual loop decoherence can take place in some systems when the size of the temporal network is large enough). Interestingly, we also find that in a small percentage of the ensemble we find $d = 1$. This apparent paradox can be explained by exploring the EDC curves for these cases (see Fig.S4). In every case where we find $d = 1$, the assignments happen to be $p_1 = 2, p_2 = 1$, and while the estimator selects $\Omega(E^1_t \| E^1_t) = 4$ instead of 3, notice that the EDC curve is essentially flat at that neighbourhood.

E. Epidemic Spreading defined on top of models 1 and 2

Models 1 and 2 above provide examples where the scalar memory $\Omega(\mathcal{G})$ of a temporal network is different from the effective memory $\Omega_{\text{eff}}(\mathcal{G})$, due to the emergence of virtual loops that affect the local memory structure of the network. Now the question is, to what extent do the virtual loops have a truly measurable effect and are therefore relevant in practice? Here, we consider a spreading process over a temporal network. We show that the dynamics of this spreading process are indeed highly sensitive to the shape of memory, and that in the event a representative scalar quantity was required, we demonstrate accordingly that $\Omega_{\text{eff}}(\mathcal{G})$ is better suited to quantifying the real effects of memory than $\Omega(\mathcal{G})$, this last quantity being blind to any virtual loop contribution.

We have implemented a Susceptible-Infected (SI) model for spreading dynamics on our two network toy models. In the SI model a node can be in one of two states: Infected (I) and Susceptible (S). At each time step an infected node has a probability λ of passing an infection to any other node that it is connected to via a link. Once a node is infected, it cannot become susceptible again, the change is permanent. In our set-up we start the infection at node 1. The infection will then be transmitted over link 1 at time t with a probability λ if $E^1_t = 1$, while it will not be transmitted if $E^1_t = 0$. Hence node 1 can infect node 2, then from node 2 the infection can cross link 2 and finally infect the third node. To quantify the speed of the spreading process we will measure the expected time taken to infect node 3, starting from node 1, and call this the *spreading time*. This quantity can be evaluated either via Monte Carlo simulations (averaging over several realisations of the process), and also analytically. As we will discuss below, both numerical simulations and analytical results reveal that the expected spreading time does indeed depend on the virtual loops, i.e. on the precise structure of the co-memory matrix. Furthermore, we will show that these effects are well accounted for by the effective memory $\Omega_{\text{eff}}(\mathcal{G})$, while conversely they are not captured by the network memory

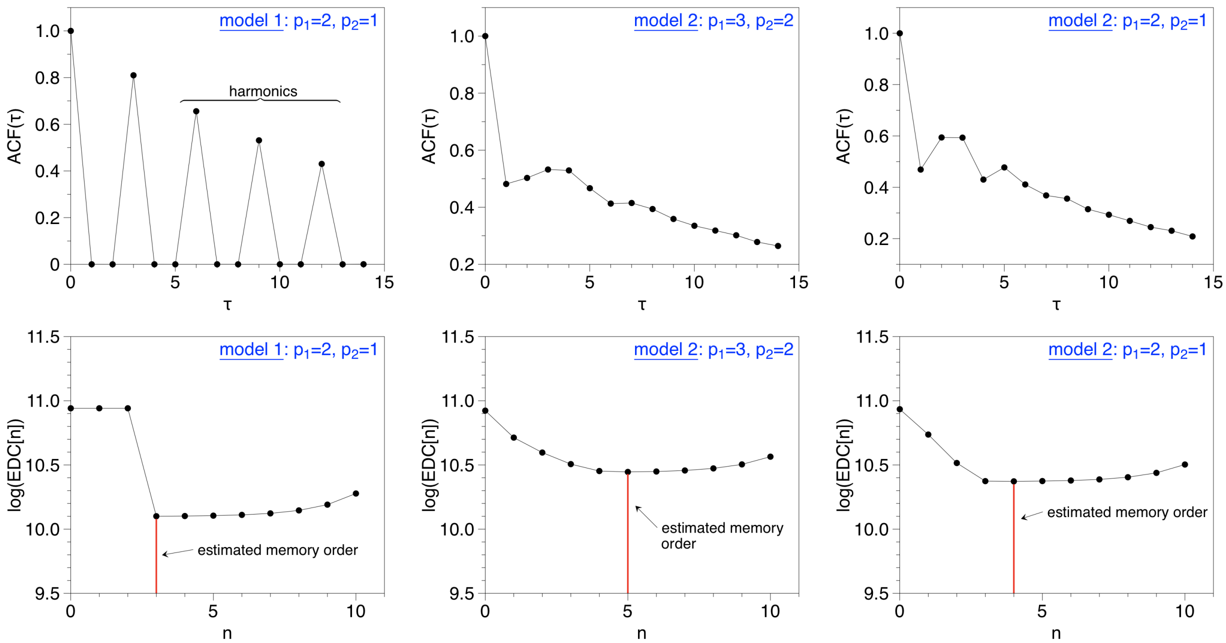

FIG. S4. **Autocorrelation function and Efficient Determination Criterion curves for models 1 and 2.** Each column depicts the autocorrelation function ACF(τ) (top) and the efficient determination criterion curve EDC(n) (bottom) of $\Omega(E_t^1 \| E_t^1)$, for one realisation of model 1 (left column) and two realisations of model 2 (middle and right columns). In every case the temporal networks have 10^5 time steps. EDC estimates that the memory corresponds to the minimum of $\log(\mathrm{EDC}(n))$. For model 1, the ACF clearly shows a peak at the correct memory $p_1 + p_2$ and a succession of additional harmonics with smaller amplitude, and the EDC curve clearly captures the correct memory order. For model 2 the situation is less obvious: the ACF cannot be so easily interpreted. The two examples depict one where the theoretical memory $p_1 + p_2 = 5$ is identified, and another where the estimation is different (note in this case that the EDC curve is relatively flat around $p_1 + p_2$).

$\Omega(\mathcal{G})$.

F. Analytical solutions of the SI dynamics on network models 1 and 2

We here present the analytical derivation of the expected spreading time for an SI infection in our toy models. We will explicitly consider *model 2*. However, the same approach, with small differences, which will be noted below, also works for model 1.
The stochastic processes $\mathcal{E}^1 = \{E_t^1\}_{t=1,2,...}$ and $\mathcal{E}^2 = \{E_t^2\}_{t=1,2,...}$ are higher-order Markov chains in the state-space $\{0,1\}$. They can then be transformed into first order Markov chains in an expanded state space. The temporal network $\mathcal{G} = \{\mathcal{E}^1, \mathcal{E}^2\} = \{E_t^1, E_t^2\}_{t=1,2,...}$ has realisation (e_t^1, e_t^2) at time t. Since each link contains memory of the other, let us build this into a pair of "state variables" α^1 and α^2, such that at a time t $\alpha^1 = \{e_t^1, e_{t-1}^1, ..., e_{t-(p_2-1)}^1\}$ and similarly for α^2. Any pair (α^1, α^2) then captures all of the useful past states of the network. Let the set of all such realisations be denoted by $\mathcal{S}$, and the sets of possible values for α^1 and α^2 be $\mathcal{S}^1$ and $\mathcal{S}^2$ respectively. Link 1 has memory of the last p_1 steps of link 2, and link 2 has memory of the last p_2 steps of link 1, hence, since the link has two possible states at any one time, $|\mathcal{S}^1| = 2^{p_2}$ and $|\mathcal{S}^2| = 2^{p_1}$. For this to be useful we must additionally introduce some concept of ordering to the values of α^1 and α^2 by means of a labelling function. The simplest form of this function, which we will use here, is

$$l(\alpha^1) = \sum_{k=0}^{p_2} 2^k \alpha_k^1, \tag{56}$$

and similarly for $l(\alpha^2)$. This is essentially taking the set of 0's and 1's that represent the link histories contained in α and converting them to a decimal number as if they were in binary. We will implicitly assume that wherever we use α, or any state in $\mathcal{S}^1$ or $\mathcal{S}^2$, we are referring to the label $l(\alpha)$.

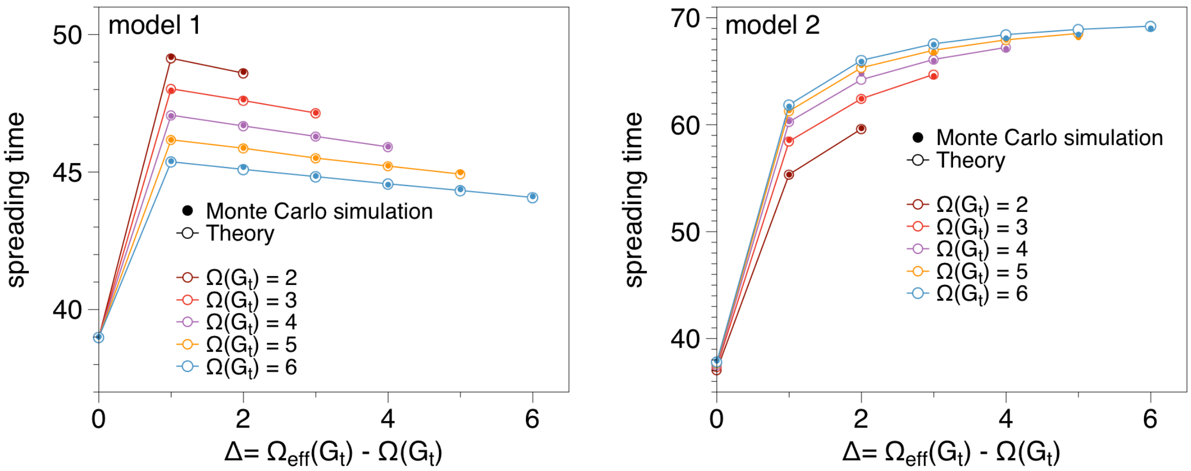

FIG. S5. Spreading times for SI dynamics on temporal networks generated by *model 1* (left) and *model 2* (right). Each curve represents networks with the same value of the scalar memory $\Omega(\mathcal{G})$. For each of these networks the effective memory $\Omega_{\text{eff}}(\mathcal{G})$ derived from the co-memory matrix is different due to the emergence of virtual loops. Spreading times are shown to vary and this variation is well captured by changes in $\Delta = \Omega_{\text{eff}}(\mathcal{G}) - \Omega(\mathcal{G})$, i.e. in the co-memory matrix. Solid symbols are the results of Monte Carlo simulations, whereas hollow symbols are the theoretical predictions obtained by solving Eq. 60.

We can use this to describe the probabilistic evolution of each link over time. For initial states $(\alpha^1, \alpha^2) \in \mathcal{S}$ and target state β^1, the probability $\mathbb{P}(\alpha^1 \to \beta^1|\alpha^1, \alpha^2)$ that link 1 goes from state α^1 to state β^1 given α^1 and β^1 defines a "transition tensor" (as opposed to the traditional transition matrix) in the following way:

$$T^{\beta^1}_{\alpha^1\alpha^2} = \left(q\frac{h(\alpha^2)}{p_1} + (1-q)y\right)\delta\left(\beta^1, 2^{p_1-1} + \lfloor\frac{\alpha^1}{2}\rfloor\right) + \left(1 - q\frac{h(\alpha^2)}{p_1} - (1-q)y\right)\delta\left(\beta^1, \lfloor\frac{\alpha^1}{2}\rfloor\right), \tag{57}$$

where h is the Hamming weight function, which counts the number of 1's in the binary representation of its argument. Note that in the case of *model 1* this would be instead

$$T^{\beta^1}_{\alpha^1\alpha^2} = \left(qs_{p_1}(\alpha^2) + (1-q)y\right)\delta\left(\beta^1, 2^{p_1-1} + \lfloor\frac{\alpha^1}{2}\rfloor\right) + \left(1 - qs_{p_2}(\alpha^2) - (1-q)y\right)\delta\left(\beta^1, \lfloor\frac{\alpha^1}{2}\rfloor\right), \tag{58}$$

where $s_p(\alpha)$ is the value of the p_{th} most significant entry in the binary representation of α, which is the state of the link at p time steps in the past. It is now a simple task to incorporate the spreading an of infection across a link: we simply associate to each link another state ι which governs the infection. $\iota^1 = 1$ if link 1 has passed an infection, and 0 if it has not. In this way the two pairs $(\alpha^1, \iota^1), (\alpha^2, \iota^2)$ completely describe the state of both the links and the infection passage over the system. What remains is to find the probabilities of an infection passing over each link given the states of the links. That is $P(\iota^1 \to \bar{\iota}^1|\beta^1, \iota^1) = {}^1\Lambda^{\beta^1,\bar{\iota}^1}_{\iota^1}$ and $\mathbb{P}(\iota^2 \to \bar{\iota}^2|\beta^2, \iota^1) = {}^2\Lambda^{\beta^2,\bar{\iota}^2}_{\iota^1}$. Denoting by H the set of link states where a link is present, i.e. $H \coloneqq \{\alpha^i : l(\alpha^i) \geq 2^{p_j-1}| j \neq i\}$, and $L_{\bar{\iota}^i} = \lambda\delta(\bar{\iota}^i, 1) + (1-\lambda)\delta(\bar{\iota}^i, 0)$, we can then write:

$$\begin{aligned} {}^1\Lambda^{\beta^1,\bar{\iota}^1}_{\iota^1} &= \delta(\iota^1, 1)\delta(\bar{\iota}^1, 1) + \delta(\iota^1, 0)\left(\chi_H(\beta^1)L_{\bar{\iota}^1} + (1-\chi_H(\beta^1))\delta(\bar{\iota}^1, 0)\right), \\ {}^2\Lambda^{\beta^2,\bar{\iota}^2}_{\iota^1} &= \chi_H(\beta^2)\left(\delta(\iota^1, 1)L_{\bar{\iota}^2} + \delta(\iota^1, 0)\delta(\bar{\iota}^2, 0)\right) + (1-\chi_H(\beta^2))\delta(\bar{\iota}^2, 0), \end{aligned} \tag{59}$$

where $\chi_H(\alpha)$ is the indicator function for α in set H. Using the transition tensor method outlined in [32] we can then find the expected spreading times $\tau_{\alpha^1,\alpha^2,\iota^1,\iota^2}$ for an infection given a starting state $\alpha^1, \alpha^2, \iota^1, \iota^2$ as the minimal solution to the following set of linear equations:

$$\tau_{\alpha^1,\alpha^2,\iota^1,\iota^2} = 1 + \sum_{\beta^1}\sum_{\beta^2}\sum_{\bar{\iota}^1}\sum_{\bar{\iota}^2} T^{\beta^1}_{\alpha^1\alpha^2} T^{\beta^2}_{\alpha^1\alpha^2} {}^1\Lambda^{\beta^1,\bar{\iota}^1}_{\iota^1} {}^2\Lambda^{\beta^2,\bar{\iota}^2}_{\iota^1} \tau_{\beta^1,\beta^2,\bar{\iota}^1,\bar{\iota}^2}. \tag{60}$$

This can then be averaged over some set of initial conditions to give the expected spreading time. In our case we will take the initial conditions to be the steady state of the network. In practice this can be viewed as the state of the network after a large number of steps. For numerical simulations we always allow the network to evolve to equilibrium before any spreading process is started, whereas for analytical calculations we take the steady state to be

the left eigenvalue of the transition matrix for the system corresponding to eigenvalue 1.

To summarise, we fix the scalar memory $\Omega(\mathcal{G})$ of the temporal network in our toy models to be p by fixing $p_1 = p$ (implicitly then $p_1 \geq p_2$). We then allow the shape of the memory in the network to vary by changing the value of p_2, and measure the expected time taken for an infection to spread over the three nodes in the network as a function of p_2. This has been done for a number of values of p, for both model 1 and model 2. The results are reported in Fig. S5 where we plot the spreading times of the SI epidemics as a function of $\Delta = \Omega_{\text{eff}}(\mathcal{G}) - \Omega(\mathcal{G})$. This value Δ is used so that curves are aligned on the x-axis for any value of p_1. Analytical results are in excellent agreement with Monte Carlo simulations and show that the quantity $\Omega_{\text{eff}}(\mathcal{G})$ is able to describe well differences in the relevant quantities that describe the dynamics of the SI process. Conversely $\Omega(\mathcal{G})$ is not able to account for the different values of spreading times obtained in networks with the same p_1 and different p_2. Indeed, in the event $\Omega(\mathcal{G})$ was well suited, then spreading rates should remain constant, as $\Omega(\mathcal{G})$ is actually constant for all temporal networks corresponding to each curve. Results indicate that the spreading time is actually not constant, and this variation correlates with the effective memory $\Omega_{\text{eff}}(\mathcal{G})$. This demonstrates that the shape of the memory, as defined by the co-order matrix $\mathbb{M}$ –and hence the effective memory– are far better at characterising the spreading rate than the scalar memory of the temporal network.

G. Inter-event time statistics

With our toy models we have demonstrated that the scalar memory of a temporal network $\Omega(\mathcal{G})$ is not necessarily the right quantity to characterise the way that memory influences the spread of an infection over the network. In order to investigate further this situation we can take a different, complementary approach. It is well known that a memoryless (Poisson) stochastic process has an exponential inter-event time distribution. Processes with memory must have inter-event times which deviate from an exponential distribution. A so-called burstiness parameter has been proposed in [33] to quantify such deviations. The burstiness parameter B of a the time series is defined as:

$$B = \frac{\sigma - \langle\tau\rangle}{\sigma + \langle\tau\rangle}, \tag{61}$$

where $\langle\tau\rangle$ and σ are respectively mean and standard deviation of the inter-event times. The expression above is equal to zero when the time series corresponds to a (memoryless) Poisson process, since for an exponential distribution the mean and standard deviation coincide. When the time series is regular, $\sigma = 0$ and thus $B = -1$, so values of B in the range $(0, -1)$ denote more regular behavior than Poisson. On the other end, for $B > 0$ the fluctuations in the inter-event times are larger than Poisson, denoting an increase of burstiness. Finally, in the limit of large σ, e.g. when the interevent time series is power law distributed, the value of B tends to 1.

Given our previous example of a spreading process, it is not clear that burstiness of the link evolution processes should a-priori be associated with the scalar memory $\Omega(\mathcal{G})$ of the temporal network. Indeed we show now that this is not the case. If the behaviour of a link in a network is bursty, then that link displays memory, and if it is not bursty then it does not have memory. Hence, we argue that for a measure of the memory of a network to accurately capture the behaviour of inter event time statistics it must reflect this: if links are not bursty then the network should not have memory, and if they are then the network should have memory. We explore this in the context of our two toy models.

We consider model 1 with two parameter settings, namely: (i) $p_1 = 1$ and $p_2 = 0$ and (ii) $p_1 = p_2 = 1$. Notice that for both choices of parameters, models 1 and 2 are equivalent.
In case (i) it is easy to see that the scalar memory and effective memory coincide, $\Omega(\mathcal{G}) = \Omega_{\text{eff}}(\mathcal{G}) = 1$, and so we expect to see little or no burstiness, whereas in case (ii) the temporal network is still first-order Markov, i.e. $\Omega(\mathcal{G}) = 1$, but the effective memory is larger, $\Omega_{\text{eff}}(\mathcal{G}) = 2$. In both cases, we have measured the burstiness parameter B as a function of the memory strength q of a link. The results reported in Fig. S6. show that case (i) is non-bursty as expected, while case (ii) displays varying degrees of burstiness, and therefore denoting the presence of a non-trivial memory shape, even if the scalar memory is still Markov. This is another indication that the memory $\Omega(\mathcal{G})$ of the network does not provide a good description of dynamical dependencies and memories at the level of links and link pairs. Which links are bursty, this being an indication of the presence of memory, is far better captured by the effective memory.

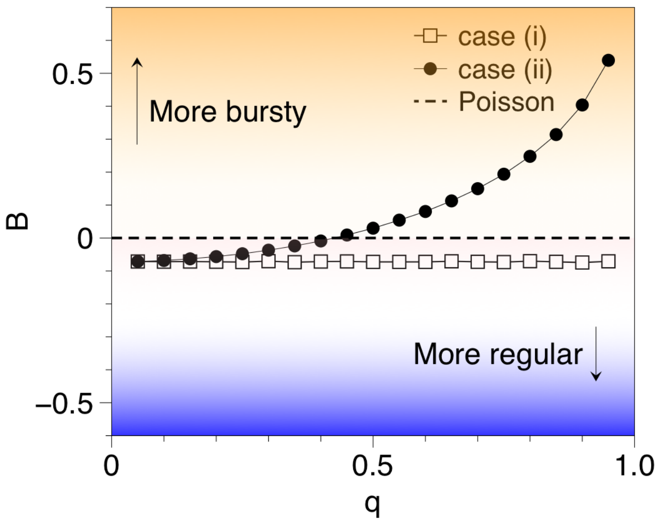

FIG. S6. Burstiness of link dynamics in model 1 as a function of the memory strength q. Two different cases have been considered (i) $p_1 = 1, p_2 = 0$, for which $\Omega(\mathcal{G}) = \Omega_{\text{eff}}(\mathcal{G}) = 1$), where $B \approx 0$ for the whole range, and (ii) $p_1 = p_2 = 1$), for which we still have $\Omega(\mathcal{G}) = 1$ but the effective memory is larger $\Omega_{\text{eff}}(\mathcal{G}) = 2$, where for a large range of values of q we find $B > 0$ which denotes burstiness in the link activity, hence highlighting the presence of memory in the internal activity of the links, a signature which is captured by $\Omega_{\text{eff}}(\mathcal{G})$ but not by $\Omega(\mathcal{G})$, as the effective memory places the networks in a regime where we can observe burstiness. Each case is simulated for 10^8 time steps.

IV. VALIDATING THE FRAMEWORK ON SYNTHETIC TEMPORAL NETWORKS

In this section we provide more details on the models for generating temporal networks with specific memory characteristics that we have discussed in the main text. In particular, we have considered four different types of generators of synthetic temporal networks: the so-called "Discrete Auto-Regressive Network of order p" (DARN(p)), extended DARN(p), "Correlated Discrete Auto-Regressive Network of order p" (CDARN(p)) and extended CDARN(p) models. We will first introduce the models, find their scalar memories, and then present a full account of their memory estimation. We will then discuss the presence of virtual loops, and show that in some cases these loops become completely decoherent for large network sizes.

A. Model definitions and ground truth proofs for $\Omega(\mathcal{G})$

All of the models here are derived from the so called "Discrete Auto-Regressive process of order p", or DAR(p) process, introduced by Jacobs and Lewis [10]. This time series incorporates a dependence on past states with random generation of new states in a simple way. In words, at each step of the process one first decides if the new state will be random or drawn from memory, if it is random then we decide what it will be, if not then we copy a single value chosen among the past p states. Formally

$$X_{t+1} = Q_t X_{t-Z_t} + (1 - Q_t) Y_t. \tag{62}$$

Here $Q_t \in \{0, 1\}$ and $Z_t \in \{1, ..., p\}$. For our purposes we only consider $X_t \in \{0, 1\}$ and so we must fix $Y_t \in \{0, 1\}$. This gives us a random process with memory p that can be extended to generate temporal networks.

- DARN(p): This model, introduced in [32], represents the simplest possible extension of the DAR(p) process to a temporal network: for a network with N nodes we assign to each of the $L = N(N-1)/2$ possible links an independent DAR(p) process. For each link $\alpha = 1, \ldots, L$ with probability q the state of the link is copied from its past, sampling uniformly at random from its own history up to p steps in the past. With probability $1 - q$ the link state will be drawn at random following a Bernoulli process with probability y. Summing up, the model depends on three parameters $\{p, q, y\}$. The first parameter y controls the density of the network. The second, q, tunes the strength of the memory term in the process with respect to the memoryless term. The final parameter, p, controls the length of the memory, which can be thought of as the number of time steps before the

autocorrelation function decays exponentially [32]. Since each link in this model is independent we can make use of corollary 1, and see that we must have $\Omega(\mathcal{G}) = p$.

- eDARN(p): The extension to the DARN(p) model, as invented for this work, is then the case where each link is allowed to have a different memory length, rather than there being a single fixed value of p. Hence we specify that each link is governed by an independent DAR(p) process but the value of p is allowed to vary for each link. This gives us a way of generating temporal networks with independent links, but with varying link memories. This model therefore depends on parameters $\{\rho(p), q, y\}$, where $\rho(p)$ is the distribution of memory lengths from which one samples the memory length of each link. If we define $\bar{p}$ as the maximum value for the memory which is drawn from the distribution $\rho(p)$, then, again, since links are independent in this model, we must have $\Omega(\mathcal{G}) = \bar{p}$ by corollary 1.

- CDARN(p): This model, as introduced in [34], represents a simple way of extending the DARN(p) model to include correlations between the dynamics of links. Similarly to the DARN(p) model, however when the link is to copy its state from memory (with probability q), it does not necessarily copy this from its own past history: with probability $1-c$ it will copy from its self, and with probability c it will choose one of the other $L-1$ links uniformly at random and copy that link state uniformly at random from the near past up to p steps in the past. Clearly the DARN(p) model is the special case of the CDARN(p) model in which $c = 0$. This model depends on parameters $\{p, q, y, c\}$.
Formally, we can define the CDARN(p) model in terms of random variables as follows: the time varying adjacency matrix $A_t = \{a_t^{ij}\}$ is given by

$$a_t^{ij} = Q_t^{ij} a_{(t-Z_t^{ij})}^{M_t^{ij}} + (1 - Q_t^{ij}) Y_t^{ij}, \tag{63}$$

where $Q_t^{ij} \sim Bernoulli(q)$, $Y_t^{ij} \sim Bernoulli(y)$, $Z_t^{ij} \sim Uniform(1,p)$ and M_t^{ij} randomly picks among the available links in the network, so that for a link process a_t^{ij}, $\mathbb{P}\left(M_t^{ij} = (i,j)\right) = 1-c$, and $\mathbb{P}\left(M_t^{ij} = (k,l)\right) = (1-c)/(L-1)$ for $(i,j) \neq (k,l)$ and $L = N(N-1)/2$. We now state and prove a theorem on the scalar memory of this temporal network process:

Theorem 4. *Let $\mathcal{G}$ be a temporal network generated by CDARN(p). Then $\Omega(\mathcal{G}) = p$.*

Proof. To prove that the scalar memory of a CDARN(p) network is indeed p, let us consider the conditional probability of observing a link directly. First, fix link (i,j), and let us associate with each link a linear label $(i,j) \to \ell \in 1, ..., L$, then the conditional probability of observing a link at time t given the past p steps is given by

$$\mathbb{P}(a_t^\ell = 1 | A_{t-1}, ..., A_{t-p}) = (1-q)y + q\left(\frac{c}{(L-1)p}\sum_{\ell' \neq \ell}\sum_{k=1}^{p} a_{t-k}^{\ell'} + \frac{(1-c)}{p}\sum_{k=1}^{p} a_{t-k}^{\ell}\right). \tag{64}$$

Since, by construction,

$$\mathbb{P}(a_t^{1,L} = 1 | A_{t-1}, ..., A_{t-p}) = \prod_\ell \mathbb{P}(a_t^\ell = 1 | A_{t-1}, ..., A_{t-p}), \tag{65}$$

and the dynamics of each link are symmetric under any relabelling of links in the temporal network, it is enough for us to consider a single link.
Now consider the same conditional, but with some number ϵ extra past steps: $\mathbb{P}(a_t^\ell = 1 | A_{t-1}, ..., A_{t-(p+\epsilon)})$. Since the memory term $t - Z_t^\ell$ will never take the values $t-(p+1)$ to $t-(p+\epsilon)$, the conditional in Eq. 64 will be unchanged, hence $\Omega(\mathcal{G}) \leq p$. Now we look at what happens when we remove some number $d < p$ of past state from the conditional, defining $\delta = p - d$:

$$\begin{aligned}\mathbb{P}(a_t^\ell = 1 | A_{t-1}, ..., A_{t-\delta}) &= \sum_{a_{t-\delta,t-p}^{1,L}} \mathbb{P}(a_t^\ell = 1 | A_{t-1}, ..., A_{t-p}) \mathbb{P}(a_{t-\delta,t-p}^{1,L}), \\ = (1-q)y \sum_{a_{t-\delta,t-p}^{1,L}} \mathbb{P}(a_{t-p}^{1,L}) &+ \sum_{a_{t-\delta,t-p}^{1,L}} \sum_{k=1}^{p} \left(\frac{q(1-c)}{p} a_{t-k}^\ell \mathbb{P}(a_{t-\delta,t-p}^{1,L}) + \frac{qc}{(L-1)p}\sum_{\ell' \neq \ell} a_{t-k}^{\ell'} \mathbb{P}(a_{t-\delta,t-p}^{1,L})\right).\end{aligned} \tag{66}$$

From this not only do we see that $\mathbb{P}(a_t^\ell = 1 | A_{t-1}, ..., A_{t-\delta}) \neq \mathbb{P}(a_t^\ell = 1 | A_{t-1}, ..., A_{t-p})$, for any such δ, assuming that $q, y \neq 1$ or 0, in which case there is no memory. Hence we must have $\Omega(\mathcal{G}) \geq p$. The only remaining option then is $\Omega(\mathcal{G}) = p$, concluding our proof. ☐

- eCDARN(p): The extension to the CDARN(p) model, as invented for this work, is one in which the memory kernel from which we sample when the link copies from its own history can be different to the memory kernel when the link copies from another link. That is, not only are links allowed to have different memory lengths, but also the memory lengths used when a link refers to its self are allowed to be different to the memory length used when referring to other links. In this way the random variable Z_t^{ij} now becomes dependent on the value of M_t^{ij}. If for a link (i,j) the value $M_t^{ij} = (i,j)$, then $Z_t^{ij} \sim Uniform(1, p_{\text{self}}^{ij})$, but if $M_t^{ij} \neq (i,j)$ then $Z_t^{ij} \sim Uniform(1, p_{\text{other}}^{ij})$, for two, possibly different, values of p_{self}^{ij} and p_{other}^{ij}. The following statement can now be proved:

Theorem 5. *Let $\mathcal{G}$ be generated by the eCDARN(p) above. Then* $\Omega(\mathcal{G}) = \max_{ij} \max(p_{self}^{ij}, p_{other}^{ij})$.

Proof. As before, we first write down the conditional probability:

$$\mathbb{P}(a_t^{\ell} = 1 | A_{t-1}, ..., A_{t-p}) = (1-q)y + q\left(\frac{c}{(L-1)p_{other}} \sum_{\ell' \neq \ell} \sum_{k=1}^{p_{other}} a_{t-k}^{\ell'} + \frac{(1-c)}{p_{self}} \sum_{k=1}^{p_{self}} a_{t-k}^{\ell}\right). \tag{67}$$

Again, by construction in this model we have

$$\mathbb{P}(a_t^{1,L} = 1 | A_{t-1}, ..., A_{t-p}) = \prod_{\ell} \mathbb{P}(a_t^{\ell} = 1 | A_{t-1}, ..., A_{t-p}), \tag{68}$$

hence $p = \min_n(n : \mathbb{P}(a_t^{1,L} | A_{t-1}, ..., A_{t-n}) = \mathbb{P}(a_t^{1,L} | A_{t-1}, ..., A_{t-\infty}))$ if and only if $p \geq \min_n(\mathbb{P}(a_t^{\ell} | A_{t-1}, ..., A_{t-n}) = \mathbb{P}(a_t^{\ell} | A_{t-1}, ..., A_{t-\infty}))$ for all ℓ and $p = \min_n(\mathbb{P}(a_t^{\ell} | A_{t-1}, ..., A_{t-n}) = \mathbb{P}(a_t^{\ell} | A_{t-1}, ..., A_{t-\infty}))$ for at least one value of ℓ. Hence, if for each ℓ we have $\min_n(\mathbb{P}(a_t^{\ell} | A_{t-1}, ..., A_{t-n}) = \mathbb{P}(a_t^{\ell} | A_{t-1}, ..., A_{t-\infty})) = \max(p_{\text{self}}, p_{\text{other}})$, then we must have $\Omega(\mathcal{G}) = \max_{ij} \max(p_{\text{self}}^{ij}, p_{\text{other}}^{ij})$. All that then remains to prove is that $\min_n(\mathbb{P}(a_t^{\ell} | A_{t-1}, ..., A_{t-n}) = \mathbb{P}(a_t^{\ell} | A_{t-1}, ..., A_{t-\infty})) = \max(p_{\text{self}}, p_{\text{other}})$. This is a trivial extension of the proof for the CDARN(p) model, but with the conditional probability now being of the form in Eq. 67. Hence we must have that $\Omega(\mathcal{G}) = \max(p_{\text{self}}, p_{\text{other}})$, as required. □

Finally, each model in practice also depends on an additional variable: the length of time series T for which it is sampled. While this does not influence the dynamics of the model it will influence any estimated value for the memory, and so we consider it here. Altogether, these models give us a wide range of test cases with a number of features that we might expect from real world networks.

Remark 1. *It's important to note that, in theory, virtual loops cannot emerge in the DARN(p) or the eDARN(p) models, but in principle should emerge in the CDARN(p) and eCDARN(p) models as in these latter cases we are probabilistically coupling links, and this coupling can induce casual loops (possibly of different orders) among sets of links. This means that we expect $\Omega_{eff}(\mathcal{G})$ to coincide with $\Omega(\mathcal{G})$ for the DARN(p) or the eDARN(p) models, but we should find a difference in the CDARN(p) and eCDARN(p) models. In the next subsection we will investigate the extent of that mismatch, and the role played by virtual loop decoherence.*

Remark 2. *In general, when a given link $\alpha \in [1, 2, \ldots, L]$ samples its future state from the past of a different link in an eCARN(p) model, one can specify which is the set of links from which α will sample from. As discussed in section IIIb, a natural way to encode this is by building up a Bayesian causal graph of L nodes, where each of the nodes corresponds to a link in the original temporal network. The $L \times L$ adjacency matrix $\mathbf{C} = \{c_{\alpha\beta}\}$ is such that $c_{\alpha\beta} = 1$ if β is in the set from which α can sample its future from, and 0 if β is not in this set.*
Different Bayesian causal graphs can thus be specified as to describe the set of links' past from which a given link copy its future state. In Fig1 of the main manuscript we choose two different examples to showcase how different link causal structures bring about different co-memory matrices. Example (a) in Fig 1 of the main manuscript has a Bayesian causal ring (i.e. $c_{\alpha\alpha\pm1} = 1$ and $c_{\alpha\beta} = 0$ for $\beta \neq \alpha \pm 1$, subject to periodic boundary conditions), where link $\alpha \in [1, 2, \ldots, L]$ updates its future state either from its past uniformly between 0 and p, or from the past of $\alpha \pm 1$. Example (b) in Fig1 of the main manuscript on the other hand has more elaborate Bayesian causal graph described in fig.S7 below, and when link α updates its future, it does so following an eCDARN(p) model by either looking at its own past and randomly sampling it between 0 and p_{self}^{α} states in the past, where $p_{self}^{\alpha} \sim Uniform\{3, 6\}$, or sampling the past of one of α's neighborhood in the link causal graph, by copying uniformly between 0 and p_{other}^{α} states in the past, where $p_{other}^{\alpha} \sim Uniform\{3, 6\}$.

Remark 3. *The suite of generative models presented above are only a selection of all the possible dynamics one could define, and we hope this work sparks the necessary interest for other researchers to investigate other classes of dynamics in a more systematic way. For instance, it would be interesting to investigate the memory shapes that*

emerge in temporal network models where (i) not all possible links can occur (i.e. the underlying "backbone" is sparse), (ii) where some interactions occur more frequently than others, (iii) or where interactions/updates occur at random points in time, to name a few options.

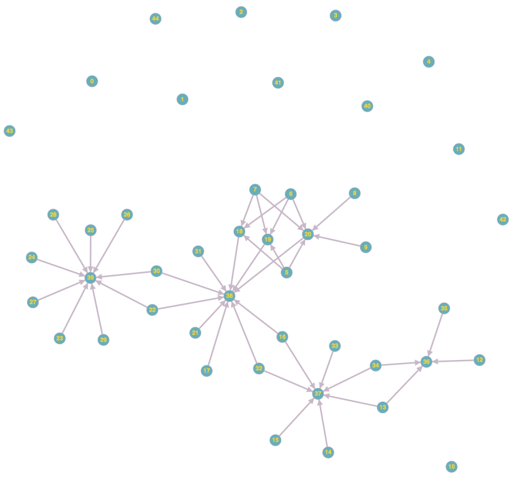

FIG. S7. Bayesian causal graph of the specific eCDARN(p) model used in the panel (b) of Fig.1 of the main manuscript. Nodes in this graph correspond to the links in the original temporal network, and two nodes are connected by a directed edge if the associated pair of links in the original network are causally connected. In this particular examples, only a subset of links in the original temporal network can actually update their state from the past of other links, where some others evolve independently.

B. Estimator accuracy on synthetic networks

For each of the four synthetic models we have described, we have studied two key quantities that help us to compare the estimated values of both Ω_{eff} and Ω_{pair} with the analytical value of $\Omega(\mathcal{G})$. These are the *hit rate* and the *average distance*, as earlier presented in Fig. S3. The hit rate gives the probability that a given estimate for the value Ω_{eff} (or Ω_{pair}) is *precisely* the scalar memory of the network, while the average distance gives the value of either $|\Omega_{\text{eff}}(\mathcal{G}) - \Omega(\mathcal{G})|$ or $|\Omega_{\text{pair}}(\mathcal{G}) - \Omega(\mathcal{G})|$, averaged over several realisations of the network model. In each case the networks generated have a fixed number of nodes $N = 10$. We then allow in turn one of the parameters q, y, T (and, where applicable c) to vary, while fixing the others to the following values: $q = 0.9, y = 0.1, c = 0.1, T = 10^6$. For each set of parameters 10^3 realisations of the model are generated, each with randomly chosen values for the memory lengths (p for DARN(p) and CDARN(p), p^{ij} for eDARN(p) and $(p^{ij}_{\text{self}}, p^{ij}_{\text{other}})$ for eCDARN(p)) from the range $1, \ldots, 10$. We then plot the hit rates and average distances for each model as a function of each free parameter in Fig. S8.

Let us discuss these results in detail. Considering the DARN(p) and eDARN(p) models:

- First, we observe that both $\Omega_{\text{eff}}(\mathcal{G})$ and $\Omega_{\text{pair}}(\mathcal{G})$ coincide with $\Omega(\mathcal{G})$ and provide a very good estimates for a wide range of parameters. Indeed they are both 100% accurate when $q = 0.9$ and y is between ~ 0.2 and ~ 0.8.

- Notice that the ranges where the performance is worse are actually expected: when y is either very small or very large, then each link series E^i_t will be dominated by either 0 or 1, in the limit of all 1's or 0's we would observe no memory as the system is deterministic, and indeed close to this we would expect it to be hard to observe any memory. This manifests as a sharp drop in both hit rate and average distance.

- When q is small we would also expect memory to be harder to detect, as it is used less often, and hence any correlations with the past are less significant. However, the increase in hit rate is a more smooth function of q, as is the average distance.

- The two memories ($\Omega_{\text{eff}}(\mathcal{G})$ and $\Omega_{\text{pair}}(\mathcal{G})$) also perform identically for both models. This is again expected: by construction there are no virtual loops of any kind in these network models, and so $\Omega_{\text{eff}}(\mathcal{G}) = \Omega_{\text{pair}}(\mathcal{G})$.

In the case of the CDARN(p) and eCDARN(p) models:

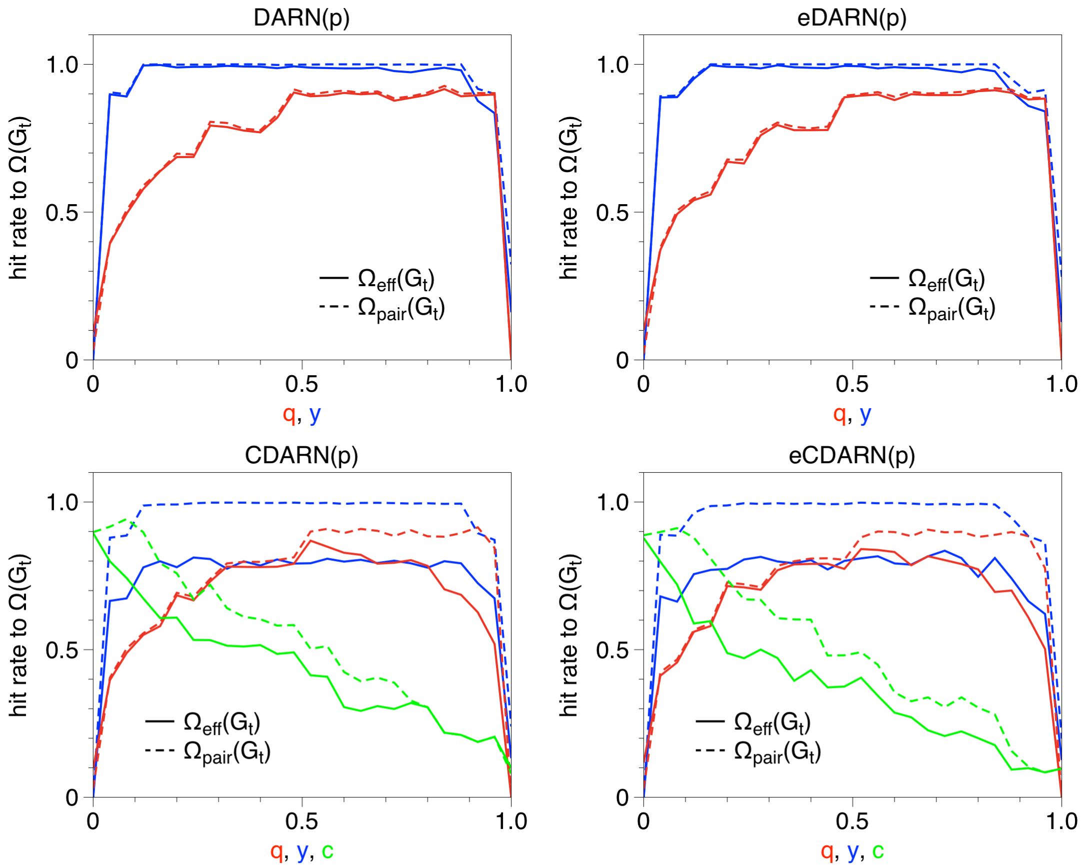

FIG. S8. **Hit rates to scalar memory $\Omega(\mathcal{G})$ for synthetic models.** In each case we compute the percentage of the times within an ensemble of 10^3 realisations that the estimated effective memory $\Omega_{\text{eff}}(\mathcal{G})$ and estimated pair memory $\Omega_{\text{pair}}(\mathcal{G})$ exactly match the scalar memory $\Omega(\mathcal{G})$ (the memory parameter p is randomly sampled from UNIFORM{1,...,10} for each realisation). Models depend on parameters q, y and (where applicable) c, so each curve scans hit rates for the whole range of a given parameter and fix the values of the other parameters to $q = 0.9, y = 0.1, c = 0.1$ (in every case, time series size is $T = 10^6$). In DARN(p) and eDARN(p) models where virtual loops are by construction absent, $\Omega_{\text{eff}}(\mathcal{G}) = \Omega_{\text{pair}}(\mathcal{G})$ and their estimation typically coincide with $\Omega(\mathcal{G})$ for a large range of model parameters, as expected. In CDARN(p) and eCDARN(p) models, (probabilistic) virtual loops are expected to kick in, inducing a mismatch between $\Omega_{\text{eff}}(\mathcal{G})$ and $\Omega(\mathcal{G})$ (the mismatch is notably smaller for $\Omega_{\text{pair}}(\mathcal{G})$ as this quantity disregards diagonal entries of the co-memory matrix).

- We observe that $\Omega_{\text{eff}}(\mathcal{G}) \neq \Omega_{\text{pair}}(\mathcal{G})$. This is again expected: note that by construction the CDARN(p) and eCDARN(p) models should have a considerable number of virtual loops, as induced by the cross-correlations that are present between each link, and indeed we would expect these virtual loops to be of a variety of orders. By definition, $\Omega_{\text{pair}}(\mathcal{G})$ cannot capture the effects of virtual loops of order greater than one, hence the mismatch between these two measures.

- Interestingly, we also observe that under a range of parameters, both Ω_{eff} and Ω_{pair} remain quite close to $\Omega(\mathcal{G})$, manifesting a strong virtual loop decoherence in these cases.

- Similarly to before, there are also ranges of both y and q where 'performance' is worse, and this is also expected.

- We also see that as $c \to 1$ the hit rate significantly drops. This can be explained by an increase in the significance of virtual loops. When $c = 0$ then links are independent, and so there are no virtual loops to influence $\Omega_{\text{eff}}(\mathcal{G})$. As we increase c we allow for virtual loops, however they will be decoherent, and so the hit rate will still initially be high. As c approaches 1, we have made the influence of virtual loops as strong as possible, and so the hit rate will be at its lowest.

C. Virtual loops in the synthetic networks

Let us further emphasise here the role played by virtual loops in the four synthetic models discussed above. The hit rate and average distance between $\Omega(\mathcal{G})$ and $\Omega_{\text{eff}}(\mathcal{G})$ demonstrate a number of features of the effects of virtual loops on memory in temporal networks. For both the DARN(p) and eDARN(p) models, where loops are not present, the values of both $\Omega_{\text{eff}}(\mathcal{G})$ and $\Omega_{\text{pair}}(\mathcal{G})$ are almost identical, and for most parameters give a very good approximation of $\Omega(\mathcal{G})$. For the CDARN(p) and eCDARN(p) models this is not the case. The two correlated models should clearly display virtual loops, as they are inherent in the structure of the models in the same way as the toy models described earlier. Indeed, we know that when calculating $\Omega_{\text{pair}}(\mathcal{G})$ then a number of the virtual loops will be accounted for, and so the significant differences between the hit rates and distances of $\Omega_{\text{eff}}(\mathcal{G})$ and $\Omega_{\text{pair}}(\mathcal{G})$ for both the CDARN(p) and eCDARN(p) models can confidently be ascribed to the presence of these loops. It is unexpected however that $\Omega_{pair}(\mathcal{G})$ accounts for the full extend of these loops; virtual loops of varying orders should be present in this system. Furthermore, in both cases we observe that $\Omega_{\text{eff}}(\mathcal{G})$ and $\Omega_{\text{pair}}(\mathcal{G})$ are reasonable estimates for the scalar memory $\Omega(\mathcal{G})$ for a range of parameter values. This can only be the case if, in the same way as for our toy models, these virtual loops display decoherence, and are hence the resulting increase in pair and effective memory is masked.

D. A theorem on full virtual loop decoherence effect for large network size

The CDARN(p) model, as we have presented here, maintains a high degree of symmetry: each link has the same memory strength q, link density y and memory length p. Each link also, with the same probability c, can look into the past p states of every other link, and will do so uniformly. In summary, the network would behave in exactly the same way if the links were swapped (in general if there were any isomorphism). One might be tempted to think that this "synchronicity" might bring out memory and virtual loops in an extreme way, since virtual loops of every order should be present in the system. Indeed we see that in our relatively small synthetic networks, when c is large, and hence there is a strong reliance on virtual loops, the scalar and effective memory of the network seldom coincide. When these networks get larger however, we observe something unexpected: all virtual loops become increasingly decoherent. As the number of links grows larger, so does the pool of links from which a past state can be drawn, because of this we find that the memory in virtual loops "averages out", a concept which we formally detail in the theorem below.

Theorem 6. *Consider a CDARN(p) network with L links, memory strength q, link density y and memory length p. The conditional probability of a link ℓ occurring at time t, given the past p states of the network is as follows:*

$$\mathbb{P}(a_t^\ell | a_{t,t-p}^{1,L}) = (1-q)y + q\left((1-c)\phi_{self} + c\phi_{other}\right), \tag{69}$$

where ϕ_{self} and ϕ_{other} represent the contributions to the conditional from the past p states of the link ℓ and every other link respectively. As $L \to \infty$, ϕ_{other} tends to a constant, and hence the link ℓ has no memory of the past states of any other link.

Proof. The conditional probability of observing a link ℓ at time t given the past p states of the network is as follows:

$$\mathbb{P}(a_t^\ell = 1 | a_{t,t-p}^{1,L}) = (1-q)y + q\left(\frac{(1-c)}{p}\sum_{k=1}^{p} a_{t-k}^{\ell} + \frac{c}{(L-1)p}\sum_{\ell'\neq\ell}\sum_{k=1}^{p} a_{t-k}^{\ell'}\right). \tag{70}$$

We can therefor see that our memory kernels ϕ_{self} and ϕ_{other} are given as:

$$\begin{aligned} \phi_{self} &= \frac{(1-c)}{p}\sum_{k=1}^{p} a_{t-k}^{\ell}, \\ \phi_{other} &= \frac{c}{(L-1)p}\sum_{\ell'\neq\ell}\sum_{k=1}^{p} a_{t-k}^{\ell'}. \end{aligned} \tag{71}$$

We need only focus on ϕ_{other}. First, let us consider the average value

$$\left\langle a_{t-k}^{\ell'} \right\rangle_{\ell'} = \mathbb{P}(a_{t-k}^{\ell'} = 1). \tag{72}$$

The CDARN(p) network is taken to be in a stationary state, and so the symmetry of the links under isomorphism guarantees us that $P(a^{\ell'}_{t-k})$ is the same for each link ℓ' and for each time $t-k$. Hence we can write $P(a^{\ell'}_{t-k}) = \bar{a}$ for some constant $\bar{a}$. Then we must have, for any of the $L-1$ possible values of ℓ',

$$\left\langle a^{\ell'}_{t-k} \right\rangle_{\ell'} = \bar{a}. \tag{73}$$

Now, ϕ_{other} can be re-written as follows:

$$\phi_{other} = \frac{1}{p}\sum_{k=1}^{p}\frac{1}{L-1}\sum_{\ell'\neq\ell} a^{\ell'}_{t-k}. \tag{74}$$

Then, by the law of large numbers we can express this in terms of the sample average:

$$\begin{aligned}\phi_{other} =& \frac{1}{p}\sum_{k=1}^{p}\left\langle a^{\ell'}_{t-k}\right\rangle_{\ell'},\\ =&\frac{1}{p}\sum_{k=1}^{p}\bar{a},\\ =&\bar{a}.\end{aligned} \tag{75}$$

Hence $\phi_{other} \to \bar{a}$ as $L \to \infty$. Since there are no terms containing links other than ℓ in ϕ_{self}, then we can conclude that the conditional probability is such that, in the same limit $L \to \infty$,

$$\mathbb{P}(a^{\ell}_{t} = 1 | a^{1,L}_{t,t-p}) \to \mathbb{P}(a^{\ell}_{t} = 1 | a^{\ell}_{t,t-p}), \tag{76}$$

and so any memory of other links is lost. □

To summarise, in this section we have validated that the co-memory matrix correctly displays the memory of synthetic temporal networks. The maximum of this co-memory matrix, defined as the effective network memory $\Omega_{\text{eff}}(\mathcal{G})$ coincides with the scalar memory $\Omega(\mathcal{G})$, and so is a good estimate of it, when virtual loops are not present. In the cases where these loops are present, the two quantities in principle differ, and we know that one should consider $\Omega_{\text{eff}}(\mathcal{G})$ rather than $\Omega(\mathcal{G})$ if in this case. Interestingly, virtual loop decoherence is also clearly present in these networks. Moreover, when the network size (number of links) gets larger, theorem 6 suggests that we asymptotically should expect full virtual loop decoherence. This is particularly important when considering real-world temporal networks. We can hence conclude that while virtual loops play a role in the memory of these networks, in practice virtual loop decoherence might limit that role, and as such we would expect that $\Omega_{\text{eff}}(\mathcal{G})$ does not substantially differ from $\Omega(\mathcal{G})$ in real, complex temporal networks.

V. APPLICATIONS TO REAL-WORLD TEMPORAL NETWORKS

A. Processing empirical network data

Here we present full results on 20 temporal networks from the real world, 7 of which were presented in the main text, and at different temporal resolutions. A varying amount of work has to be done to each of the used data sets before they can be fed into our memory estimator as a discrete time temporal network. As part of the software developed for this research, we have developed a method to convert a time stamped edge list of the form (v_1, v_2, t) into a set of time series X^{v_1,v_2}_t which represents the edge process connecting nodes v_1 and v_2. Once we have this time series then we can make use of our memory estimators. Hence the aim is now to take each data set and convert it into this time stamped edge list form. We will here give a brief overview of each dataset and the steps required to process it.

- The first two datasets correspond to a type of network which we can label as online social communication networks:
  - **CM**: Text message interactions between college students [35]. This data represents messages sent between (anonymised) student users of an online communication platform at the University of California, Irvine, over a period of 7 months. Processing this is a simple task as it comes in what is almost a suitable format; the raw data is given as triplets $(I.D_1, I.D_2, t)$ for each pair of individuals $I.D_1$ and $I.D_2$ interacting at time t measured in seconds from some starting value. Here we simply index the individuals from 0 to N and shift the t values so that the first link in the data occurs at time 0.

- **EM**: Email communications data set [36]. This data set covers internal e-mail communications between employees of a mid-sized manufacturing company over a period of nine months. The data is (ignoring other irrelevant data) in the form $(I.D_1, I.D_2, DT)$ where now DT is in a date-time format to a resolution of seconds. Hence in constructing our time stamped edge list we again index the $I.D$ values appropriately, and again convert the date-time to the number of seconds elapsed since the first link in the data set occurred.

- The third dataset is a social interaction or contact network (**RM**), that is to say, it is also a social network like the first two cases, but it is an 'offline' one and can also be seen as a mobility network. This data is taken from the "Reality Mining" data set [37], collected from the interaction of 94 students at MIT over 8 months. The data we have used here was taken from bluetooth interactions between the phones carried by the subjects of the study. Each interaction indicates that the two individuals were within at least 5-10 meters of each other. Each device scans for other devices in its proximity every 5 minutes, however because a pair of devices can recognise each other independently this can produce two interactions every 5 minutes with an average inter interaction duration of 2.5 minutes. The data is (ignoring other irrelevant data) in the form $(I.D_1, I.D_2, DT)$ where now DT is in a date-time format to a resolution of seconds. Hence in constructing our time stamped edge list we again index the $I.D$ values appropriately, and again convert the date-time to the number of seconds elapsed since the first link in the data set occurred.

- The next 9 datasets correspond to what we could label as **engineered transportation networks** designed for the public transport in Berlin (B), Paris (P) and Rome (R) [38] via bus (Bb, Pb and Rb), train (Bt, Pt and Rt) and underground (Bu, Pu, and Ru), i.e. these are engineered, offline infrastructure networks. These are comprised of a weeks worth of records for the movements of public transport systems from stop to stop. This data is structured in a different way; each set contains the times taken for each journey occurring between any two stops on the bus (–b), train (–t) and underground (–u) public transport systems in their respective cities. This data (again ignoring irrelevant data) is of the form $(I.D_1, I.D_2, t_{start}, t_{stop})$, where the values $I.D_1$ and $I.D_2$ represent the origin an destination stops respectively, and t_{start} and t_{stop} represent the date-times at which the origin was left and the destination was reached respectively, to a resolution of one second.
  We here index the origin and destinations as expected, and convert the date-time values to the number of seconds elapsed since the first date-time value in the data set. We then take the link between the origin and destination to be present in every second which elapses between t_{start} and t_{stop}. If two journeys overlap then the link is kept active until the latter of the two journeys is completed.

- The next 4 datasets represent **proximity networks from football matches**. They refer to football teams over two matches, and for both sides separately (home h, away a), extracted from `https://github.com/metrica-sports/sample-data`. This gives 4 networks labelled F1h, F1a, F2h, F2a. Links in this network are formed when two players on the same side are within some threshold distance r from each other. For our purposes this distance is set to 10 metres. The initial data is presented as a collection of triplets (w^i, h^i, t) for each player i representing the scaled distance along the length of the pitch w^i, across the pitch h^i, and the time. The scaling of w and h is taken such that $w, h \in [0,1]$, however, the pitches are always of dimensions: $105 times 68$ meters, as such the exact position x^i, y^i of each player i can be calculated for each time t. Locations are sampled every 0.04 seconds. Each individual player on each side is given a unique number, for simplicity these are then mapped to the interval $\{0, ..., 13\}$, and are considered the nodes in our temporal network. At each time t, each possible link e_t^α with $\alpha = (i,j)$, $i,j \in \{0, ..., 13\}$, $i \neq j$ takes state $e_t^\alpha = 1$ if $\sqrt{(x^i - x^j)^2 + (y^i - y^j)^2} \leq r$, and 0 otherwise.

- The final 4 datasets represent time-varying **functional cortical networks from the human brain**, as extracted from high-resolution electroencephalography EEG recordings. These recordings were taken from a group of five subjects performing a foot movement [39], with each network corresponding to a time period of 0.5 seconds: the final phase of execution of the foot movement. Our four networks correspond to the four frequency bands $\theta, \alpha, \beta, \gamma$, giving networks B$\theta$, B$\alpha$, B$\beta$ and Bγ. Each temporal network has 16 nodes, representing cortical regions of interest, and has 400 time-steps. Each link represents causal influences between cortical regions see Ref. 11 for details. The data is presented, for each frequency band, as a set of 400 adjacency matrices A_t, which are converted directly to the required time-stamped edge list format.

Coarse-graining at different resolution timescales – Once we have a time stamped edge list for a data set we filter out the 100 links which occur the most (though only where there are at least 100 links). For the networks CM, EM, RM and all transport modes/cities, we then produce two temporal networks by, for each link, integrating over two different timescales $\Delta t = 1$ minute (60 seconds) and $\Delta t = 10$ minutes (600 seconds). That is to say, given a temporal network $\mathcal{G}$ with edge processes E_t^i, and with a unit timescale, which extends from time $t = 0$ to $t = T$, we define a new temporal network $\tilde{\mathcal{G}}$ with edge processes $\tilde{E}_k^i$, and with timescale $\Delta t = 60, 600$ seconds, which extends

from time $k = 0$ to $k = T/\Delta t - 1$. The links in this second network are then drawn from the first in the following way: $\tilde{E}^i_k = 1$ if for any $t \in \{k\Delta t, ..., (k+1)\Delta t\}$, $E^i_t = 1$. In this way we effectively "integrate" the time series for each link over our time scale Δt. For the football networks this process is repeated for $\Delta t = 5, 10$ and 30 seconds.

B. Co-order histograms and memory shapes at different time scales

The memory shapes of all 20 networks are reported in Fig.S9 (for the social networks and the EEG ones only one resolution is depicted, for transportation and football we represent several ones). The co-order histograms are then plotted in Fig.S10.

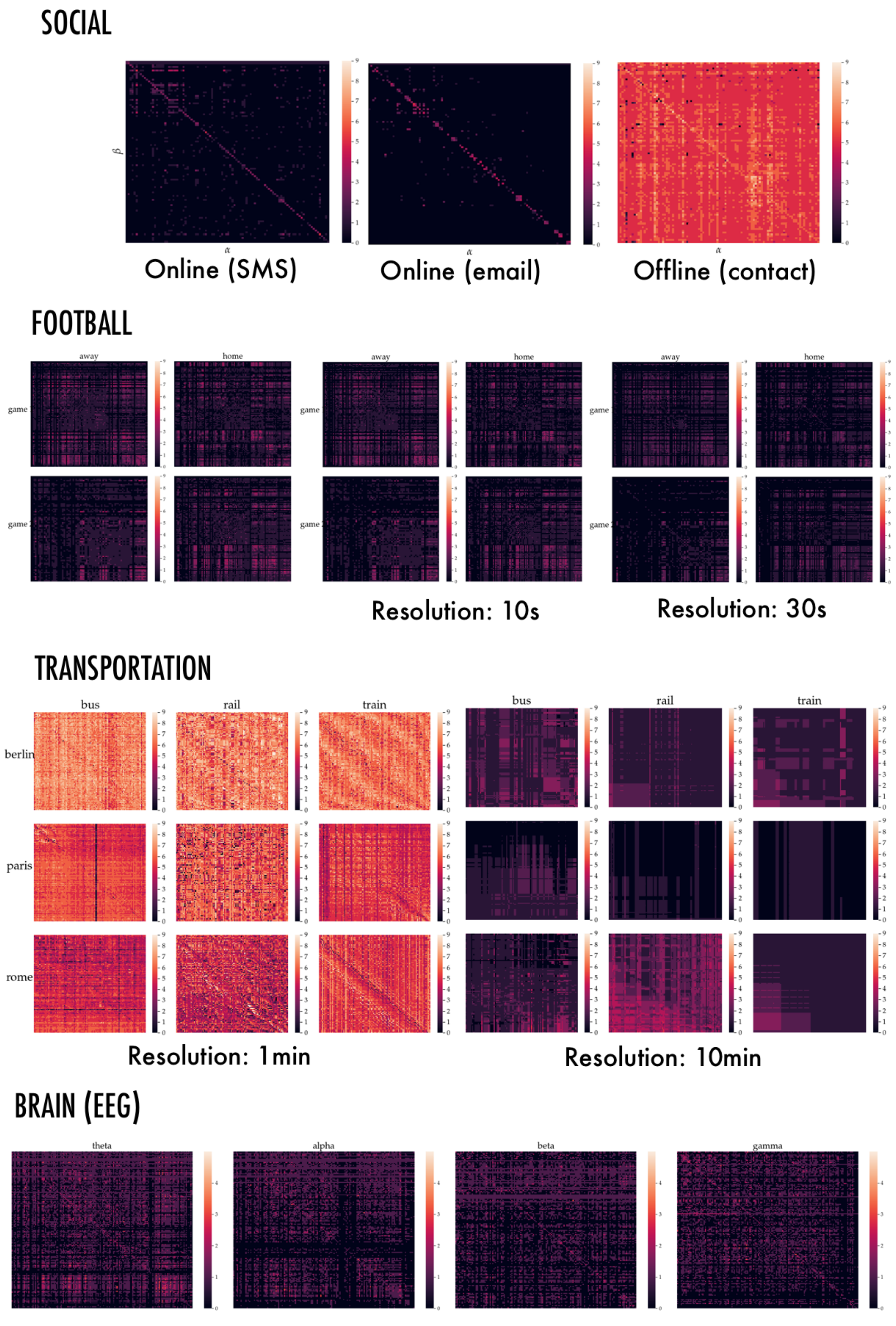

FIG. S9. **Memory shapes for all networks.** The full memory shape matrix observed for each temporal network and for each timescale. Networks in the "social" and "transportation" categories are shown for timescales $\Delta t = 1$ and 10 minutes, in the "football" category for $\Delta t = 5$, 10 and 30 seconds, and for the "brain" category we use the four frequency bands $\alpha, \beta, \gamma, \theta$. All cases display a range of co-orders across the matrix, including for entries that do not lie on or close to the diagonal, indicating the importance of the memory shape in the networks evolution and the presence of non-trivial causal structures in all cases.

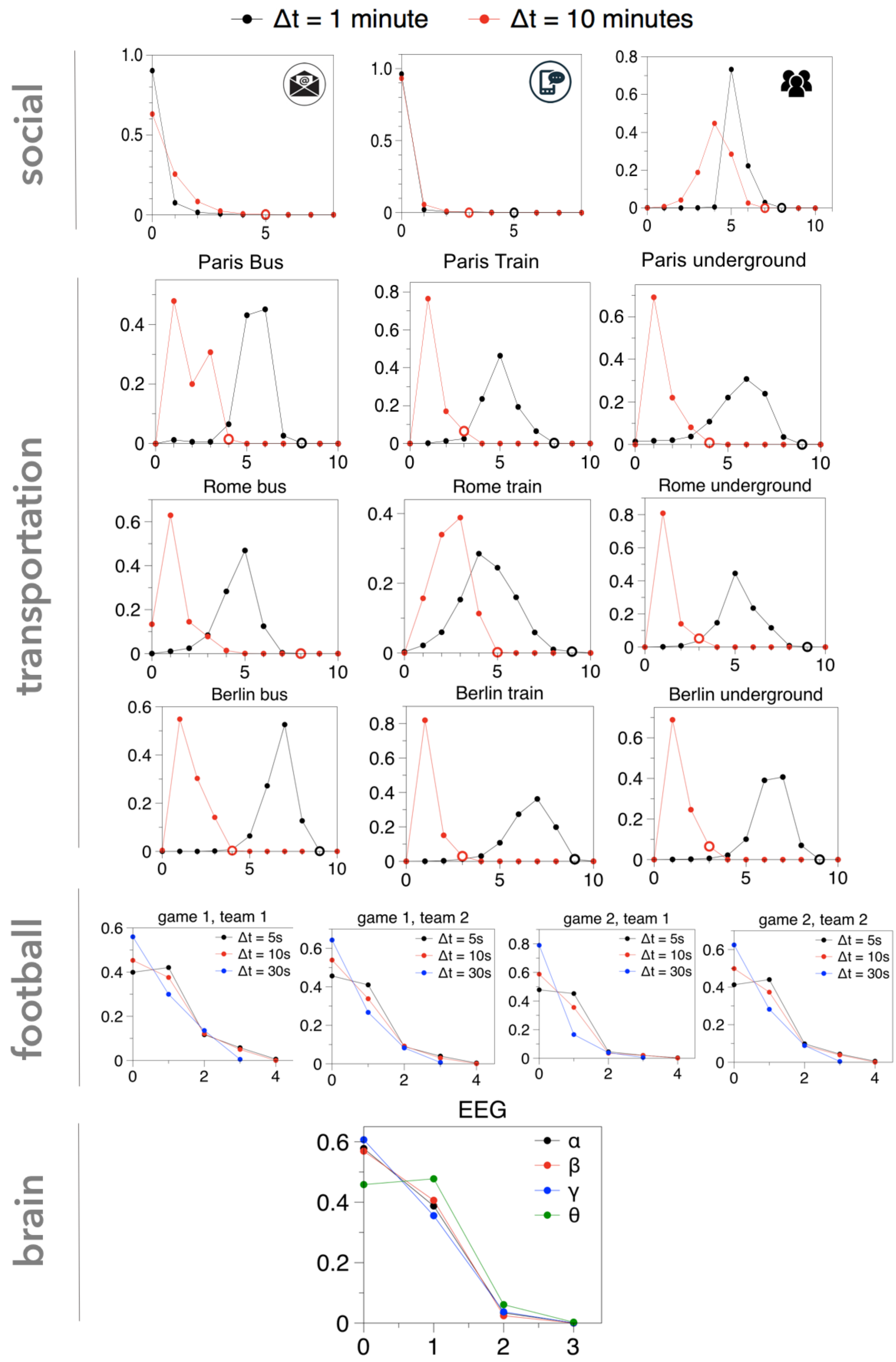

FIG. S10. **Histograms of co-orders for all networks.** The fraction of co-orders observed for each temporal network and for each timescale. Networks in the "social" and "transportation" categories are shown for timescales $\Delta t = 1$ and 10 minutes, in the "football" category for $\Delta t = 5$, 10 and 30 seconds, and for the "brain" category we use the four frequency bands $\alpha, \beta, \gamma, \theta$. All cases display a distribution of co-orders above 1, indicating the importance of the memory shape in the networks evolution.

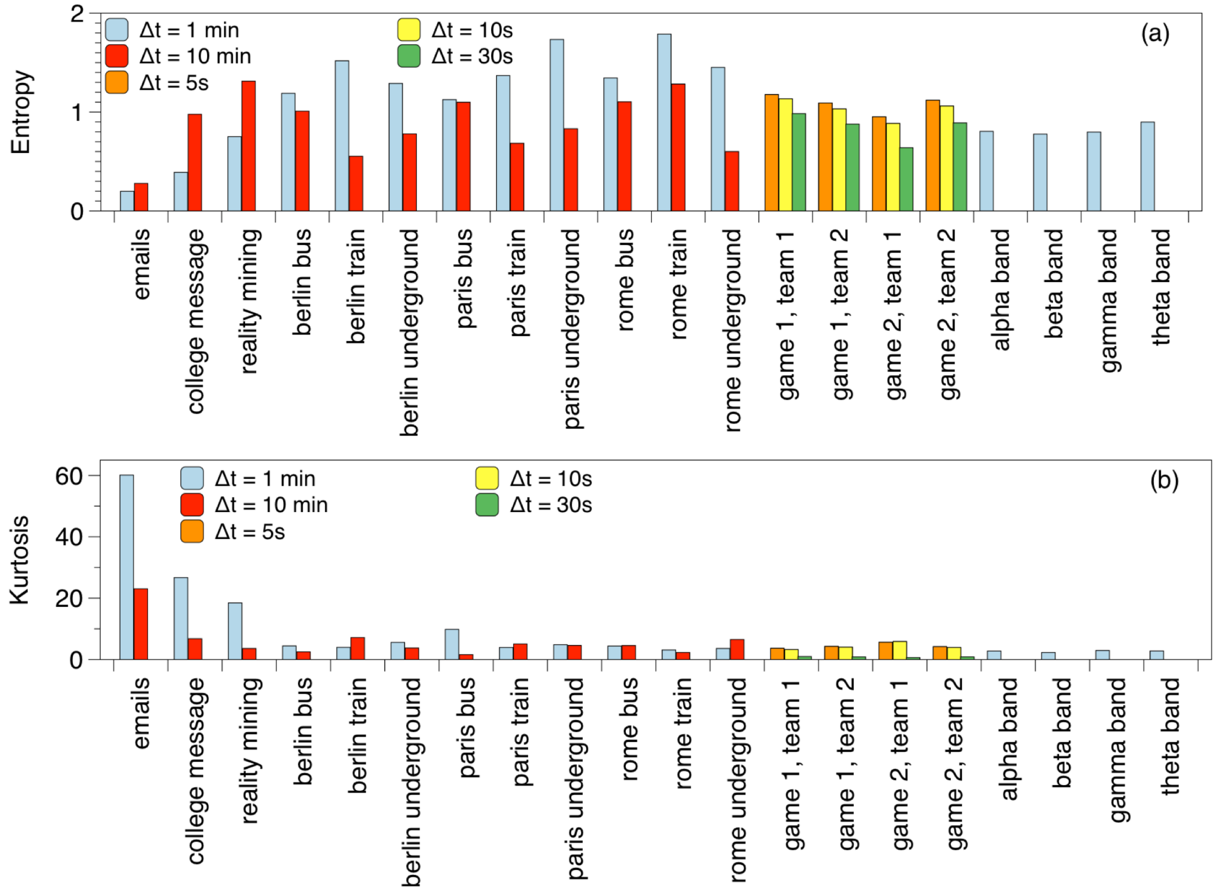

FIG. S11. **Assessing co-memory histogram heterogeneity** Entropy (panel a) and kurtosis (panel b) of the co-memory histogram for each of the 20 real-world temporal networks, at $\Delta t = 1$ min (blue) and $\Delta t = 10$ min (red) for the networks in the "social" and "transportation" categories, $\Delta t = 5$ (orange) , 10 (yellow) and 30 seconds (green) for the "football" category, and the four frequency bands $\alpha, \beta, \gamma, \theta$ for the "brain" category.

C. Heterogeneity of co-memory histograms: entropy and kurtosis

In Figure 3 of the main manuscript we have plotted the co-memory matrix histograms for the seven temporal networks at the two resolution timescales, and in Fig.S10 we report all of them. Here we give a further exploration about the shape of these histograms by computing their *entropy* and *kurtosis*. The entropy of a probability distribution function $p(x)$ is defined as $-\sum p(x)\log p(x)$ and characterises how 'uneven' the distribution is, reaching a maximum when the distribution is uniform and reaching the minimum (zero) when the probability is fully concentrated. Accordingly, entropy encapsulates in a scalar metric how heterogeneous the microscopic memory kernel of a given temporal network is. The kurtosis is the fourth standardised moment of $p(x)$, and is a measure of its "tailedness".

Results for all networks at all resolution scales are shown in Fig.S11. The fact that transportation networks tend to have large entropy suggest that many different memory co-orders are detected, i.e. these networks display a highly heterogeneous memory kernel. On the other hand, we find that the online social networks have a strong kurtosis, meaning that even if most of the links have a weak memory kernel, there are a few whose co-order is large. Since $\Omega_{\text{eff}}(\mathcal{G})$ is defined as the maximum over all co-orders, from the kurtosis analysis we can conclude that online social communication networks analysed in this work display large memory but this only comes from a handful of links.

D. Constructing memory communities and comparing networks in the $(\langle\Omega\rangle^{\alpha}_{\text{in}}, \langle\Omega\rangle^{\alpha}_{\text{out}})$ plane

Given the memory heterogeneity displayed in real temporal networks, we wish to further understand how much the past history of the whole network influences the activity of a given link, and, in turn, how much the past history of a given link has an influence on the evolution of the network. To quantify this we define two quantities: given the time series $\mathcal{E}^{\alpha}_t$ representing the evolution of each link $\alpha \in 1, ..., L$, the *average incoming co-order* of link α is defined as

$$\begin{aligned}\langle\Omega\rangle^{\alpha}_{\text{in}} &= \frac{1}{L}\sum_{\beta}\Omega(\mathcal{E}^{\alpha}_t \| \mathcal{E}^{\beta}_t),\\ &= \frac{1}{L}\mathbb{M}\underline{1}.\end{aligned} \tag{77}$$

This quantity characterises the average memory length that link α has of the network as a whole. On the other hand, we define the *average outgoing co-order*

$$\begin{aligned}\langle\Omega\rangle^{\alpha}_{\text{out}} &= \frac{1}{L}\sum_{\beta}\Omega(\mathcal{E}^{\beta}_t \| \mathcal{E}^{\alpha}_t),\\ &= \frac{1}{L}\mathbb{M}^{T}\underline{1},\end{aligned} \tag{78}$$

characterising the average memory length that the network retains of the past activity of link α. The ordered pair (2-tuple) $(\langle\Omega\rangle^{\alpha}_{\text{in}}, \langle\Omega\rangle^{\alpha}_{\text{out}})$ is therefore a compact representation of the role played by each link α, in this section we explore scatterplots of $\langle\Omega\rangle^{\alpha}_{\text{out}}$ vs $\langle\Omega\rangle^{\alpha}_{\text{in}}$. More concretely, for each of the 20 empirical temporal networks we have considered in this work, we focus on the top 100 most active links and make scatter plots of $\langle\Omega\rangle^{\alpha}_{out}$ vs $\langle\Omega\rangle^{\alpha}_{in}$ for the two different resolution timescales $\Delta t = 1$ and 10 minutes.

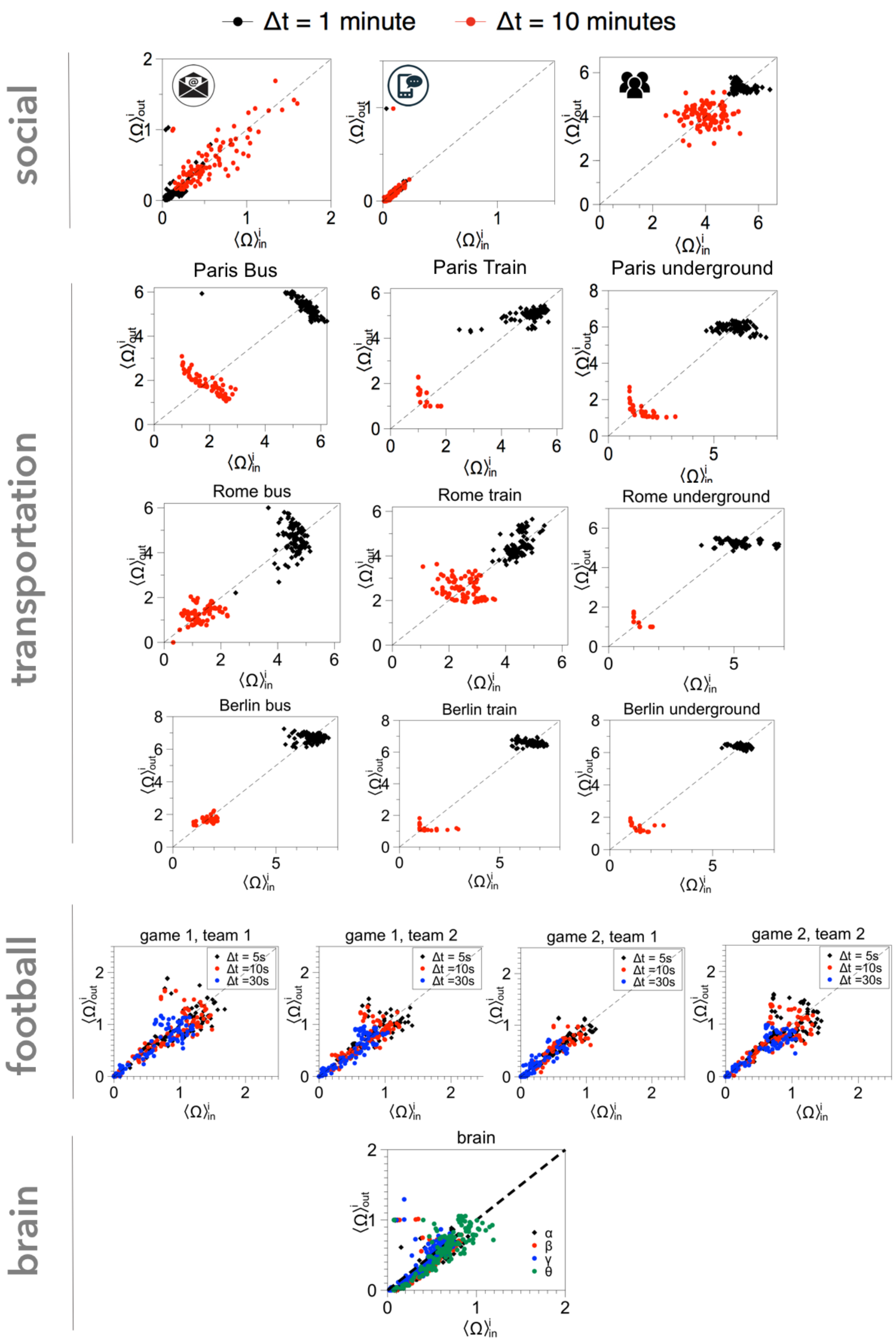

FIG. S12. **Comparison between the resolution timescales for all networks.** For the 20 real-world temporal networks we scatter plot the average outgoing co-order $\langle\Omega\rangle^{\alpha}_{out}$ vs the average incoming co-order $\langle\Omega\rangle^{\alpha}_{in}$ of the 100 most active links (or all links in the case of the "football" and "brain" networks), and we systematically compare these for the sampled resolution timescales: $\Delta t = 1$ min (black diamonds) and $\Delta t = 10$ min (red dots) for the "social" and "transportation" networks, $\Delta t = 5$ (black diamond) ,10 (red dot) and 30 seconds (blue dot) for the "football" category, and the four frequency bands α (black), β (red), γ (blue), θ (green) for the "brain" category.. The online social interaction networks (EM, CM) have weak memory kernels which are maintained at the two resolution timescales. The offline social contact network (RM) have strong memory at both resolutions, but memory is overall larger at the lower resolution, thereby making timescale separation easy. All engineered transportation networks display strong memory kernels only at the lower resolution timescale with the exception of the Rome train network. Football and brain networks generally have weaker kernels at all time scales, and are clustered along the diagonal in a similar manner to the online social networks.

Let us start by comparing, for each temporal network, these scatter plots for the different resolution times. Results are plotted in the panels of Fig.S12. The first observation is that transportation networks (bus (PB), train (PT) and underground (PU)) display very different memory properties at the $\Delta t = 1$ and $\Delta t = 10$ min resolution timescales, and hence their links systematically cluster apart. More particularly, for the $\Delta t = 10$ the average co-orders are notably lower than for the $\Delta t = 1$ scale, suggesting indeed that all the memory structure is captured at the $\Delta t = 1$ scale, i.e. only one memory scale manifests, as expected due to strong planning and scheduling restrictions.
At the other extreme, many links in the two *online social communication* networks overlap in the scatterplots for the two resolution timescales, and memory is systematically weak. This effect is more acute in the college text message (CM) network than in the email (EM) network. Incidentally, for the CM network we find that there is a single link which, for both $\Delta t = 1$ and 10 min timescales, has a significantly larger $\langle\Omega\rangle^{\alpha}_{\mathrm{out}}$ than the rest, meaning that there is one specific link whose activity is driven by the global activity of the network. The *offline social contact network* (RM) somehow interpolates the behaviour of the previous two groups: we can see that while links for the two timescales are closer together, they still cluster apart and the two different timescales are clearly visible. The memory of this network is strong at the two different timescales, concluding that we are indeed detecting two different memory timescales. Finally, the projection of both football and EEG networks display a similar structure in this plane that the one found for online social networks, with overall weak memory.

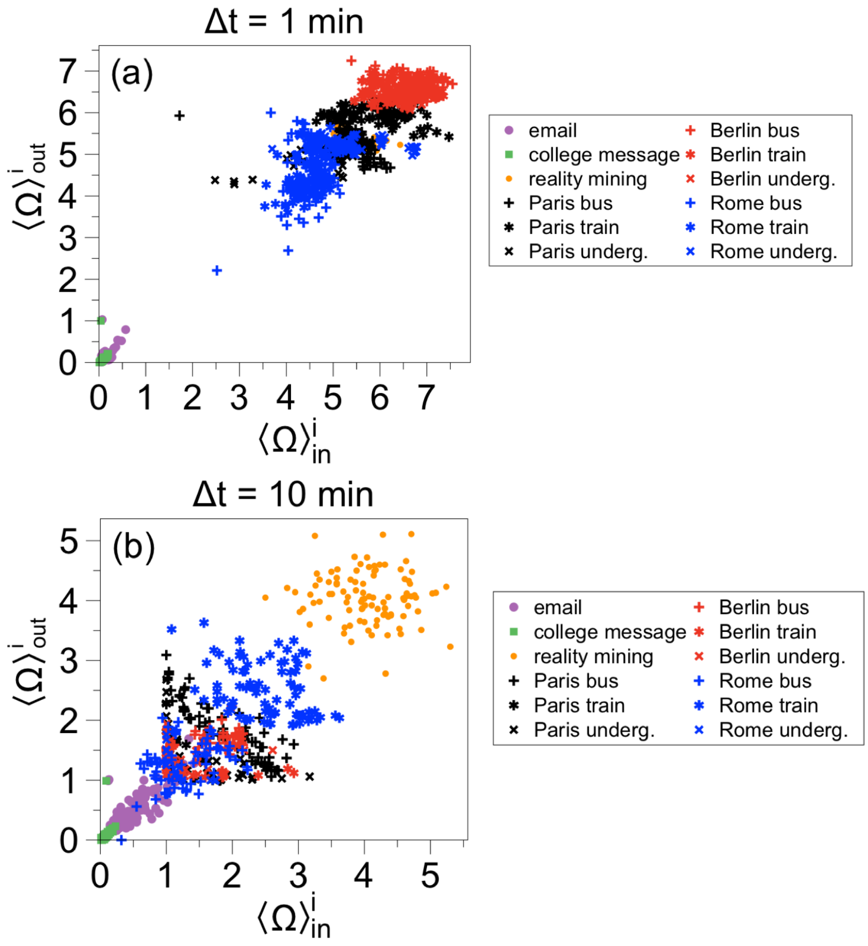

FIG. S13. **Scatter plot of the average outgoing co-order $\langle\Omega\rangle^{\alpha}_{out}$ vs the average incoming co-order $\langle\Omega\rangle^{\alpha}_{in}$ of the most active links** for the 12 real-world temporal networks in the "social" and "transport" categories at the (a) $\Delta t = 1$ min and (b) $\Delta t = 10$ min resolution timescales. At the lower resolution timescale online and offline temporal networks cluster together, and offline networks evidence stronger microscopic memory kernels. At the larger resolution timescale, only the (offline) social contact network (RM) displays consistently strong memory. We also observe a clustering of the transportation networks at both timescales.

Secondly, we explore how these temporal networks compare to each other when projected in the $(\langle\Omega\rangle^{\alpha}_{\mathrm{in}}, \langle\Omega\rangle^{\alpha}_{\mathrm{out}})$ plane. In Fig. S13 we scatterplot the top 100 most active links for all 12 temporal networks which have a $\Delta t = 1$ min

(panel (a)) and $\Delta t = 10$ min (panel (d)) resolution. The first observation is that at the $\Delta t = 1$ min, the two social communication networks cluster together, and the same is true for the three engineered transportation networks. Furthermore, there is a very clear separation between these two groups, indicating that the memory structure is very different: the online networks clearly display weaker memory than the offline ones. Perhaps unexpectedly, the social contact network (RM) clusters together with the engineered transportation networks (in particular, there is a large overlap with the bus network). Note however that, even if RM is a social network, it is (i) offline, like the second group, and also (ii) it is a mobility network, and therefore it is not unreasonable to find that its memory structure is more akin to the one displayed by a transportation one. When we switch the resolution scale to $\Delta t = 10$ min, we observe that the engineered transportation networks are still grouped together, however they lose memory and therefore come closer to the social communication networks. As discussed before, the social contact network retains its high memory at the larger timescale, this being a manifestation of the second memory timescale present in this network.

We turn now to explore and quantify the extent to which links are clustered in the $(\langle\Omega\rangle^{\alpha}_{\mathrm{in}}, \langle\Omega\rangle^{\alpha}_{\mathrm{out}})$ plane, by finding a 2-dimensional equivalent to their standard deviation σ of the set of L points representing each data set:

$$\sigma = \sqrt{\frac{1}{L-1}\sum_{\alpha=1}^{L}\left(\langle\Omega\rangle^{\alpha}_{in} - \bar{\Omega}_{in}\right)^2 + \left(\langle\Omega\rangle^{\alpha}_{out} - \bar{\Omega}_{out}\right)^2}, \tag{79}$$

where $\bar{\Omega}_{in}$ and $\bar{\Omega}_{out}$ are the sample averages of $\langle\Omega\rangle^{\alpha}_{in}$ and $\langle\Omega\rangle^{\alpha}_{out}$ respectively. If this value is large then there is a high average distance between points, and so we can think of them as being less clustered. If this value is small then the points are close together and we can think of them as being highly clustered. Results for this metric for each network are shown in table II. This shows us that for $\Delta t = 1$ min the online social interaction networks (EM, CM) are far more clustered than the engineered networks, with the highest value of σ for the social networks being 0.132, while the lowest value for engineered networks being 0.461, a fact which remains true for $\Delta t = 10$ min. The offline social contact network (RM) displays systematic spread, highlighting large memory link heterogeneity at different resolution times.

Next, we consider the *input-output memory balance*, which measures how balanced the influence of the network is on each link compared to each links influence on the network. For instance, if for all links we have $\langle\Omega\rangle^{\alpha}_{in} \approx \langle\Omega\rangle^{\alpha}_{out}$ then we know that no link has a strong influence on the memory of the network, and equally no link has long memory of the rest of the network. To assess this we take the average distance from the line $\langle\Omega\rangle^{\alpha}_{out} = \langle\Omega\rangle^{\alpha}_{in}$ for each network. More concretely, for each empirical network we find the average distance D between the points in the scatter plot to the line $\langle\Omega\rangle^{\alpha}_{in} = \langle\Omega\rangle^{\alpha}_{out}$, given L sampled links, as follows:

$$D = \frac{1}{L\sqrt{2}}\sum_{\alpha=1}^{L}\left|\langle\Omega\rangle^{\alpha}_{in} - \langle\Omega\rangle^{\alpha}_{out}\right|. \tag{80}$$

The values of these metrics for each network are shown again in table II. Here we find that, as before, online social networks are far more balanced than transportation networks, while the offline social network sits between the other two.

**Network**	σ		D	
Δt	1	10	1	10
CM	0.113	0.115	0.017	0.019
EM	0.198	0.456	0.044	0.093
RM	0.335	0.716	0.194	0.417
BB	0.495	0.492	0.271	0.234
BT	0.470	0.382	0.285	0.213
BU	0.343	0.428	0.213	0.317
PB	0.712	0.745	0.421	0.623
PT	0.636	0.465	0.329	0.342
PU	0.581	0.562	0.229	0.396
RB	0.724	0.548	0.378	0.242
RT	0.580	0.668	0.240	0.423
RU	0.636	0.417	0.327	0.333

TABLE II. Clustering metrics for each empirical data set in the "social" and "transport" categories, and for time resolutions $\Delta t = 1$ and 10 minutes.

Network	σ			D		
Δt	5	10	30	5	10	30
F1a	0.671	0.652	0.578	0.130	0.132	0.101
F1h	0.533	0.508	0.466	0.101	0.091	0.071
F2a	0.438	0.406	0.325	0.056	0.062	0.052
F2h	0.575	0.558	0.495	0.121	0.111	0.083

TABLE III. Clustering metrics for each empirical data set in the "football" category and for time resolutions $\Delta t = 5, 10$ and 30 seconds.

Frequency	σ	D
α	0.357	0.119
β	0.326	0.112
γ	0.315	0.060
θ	0.398	0.128

TABLE IV. Clustering metrics for each empirical data set in the "brain" category and for α, β, γ and θ frequency bands.

1. Transformed coordinates

An important feature of the projection onto the $(\langle\Omega\rangle^{\alpha}_{in}, \langle\Omega\rangle^{\alpha}_{out})$ plane is that such a projection is, by construction, balanced. By this we mean that the "centre of mass" of the points in the scatter plot, as defined by the expectation of each coordinate $(\mathbb{E}\left[\langle\Omega\rangle^{\alpha}_{in}\right], \mathbb{E}\left[\langle\Omega\rangle^{\alpha}_{out}\right])$, is always such that $\mathbb{E}\left[\langle\Omega\rangle^{\alpha}_{in}\right] = \mathbb{E}\left[\langle\Omega\rangle^{\alpha}_{out}\right]$. This can be seen as follows:

$$\begin{aligned} \mathbb{E}\left[\langle\Omega\rangle^{\alpha}_{in}\right] &= \frac{1}{L}\sum_{\alpha=1}^{L}\langle\Omega\rangle^{\alpha}_{in} \\ &= \frac{1}{L^2}\underline{1}^T\mathbb{M}\underline{1} \\ &= \frac{1}{L^2}\underline{1}^T\mathbb{M}^T\underline{1} \\ &= \frac{1}{L}\sum_{\alpha=1}^{L}\langle\Omega\rangle^{\alpha}_{out} \\ &= \mathbb{E}\left[\langle\Omega\rangle^{\alpha}_{out}\right]. \end{aligned} \tag{81}$$

In other words, the centre of mass of the points in the scatter plot lies on the diagonal for any network . This gives us a natural way to classify the links of a network and partition them into two classes: "influencers", i.e. those links whose past activity has more influence on the activity of the rest of the network than the network past activity has on them, and "followers", that are instead those links more influenced by the past activity of the network. The two types of links correspond to points respectively located above or below the diagonal in the scatter plot. In order to study the organization of the links of a network into followers and influencers, and to investigate whether a network is more heavily biased towards links in one class or the other, we first perform a change of coordinates $(\langle\Omega\rangle^{\alpha}_{in}, \langle\Omega\rangle^{\alpha}_{out}) \rightarrow (h, d)$, where h is the distance along the diagonal, and d is the (signed) distance away from the diagonal. The new coordinates can be expressed in terms of the old ones as:

$$\begin{aligned} h^{\alpha} &= \sqrt{(\langle\Omega\rangle^{\alpha}_{in})^2 + (\langle\Omega\rangle^{\alpha}_{out})^2}\cos\left(\frac{\pi}{4} - \arctan\frac{\langle\Omega\rangle^{\alpha}_{in}}{\langle\Omega\rangle^{\alpha}_{out}}\right), \\ d^{\alpha} &= \sqrt{(\langle\Omega\rangle^{\alpha}_{in})^2 + (\langle\Omega\rangle^{\alpha}_{out})^2}\sin\left(\frac{\pi}{4} - \arctan\frac{\langle\Omega\rangle^{\alpha}_{in}}{\langle\Omega\rangle^{\alpha}_{out}}\right). \end{aligned} \tag{82}$$

Using the new coordinates we look at the skewness and kurtosis of the distribution of the points representing the links of a given network. These two quantities respectively measure how asymmetric and how "fat tailed" the distributions of the links of a network are both along and perpendicular to the diagonal. The results are shown in Fig.S14. We see that the majority of networks have a negative perpendicular skew, indicating that links tend to be "followers" (whereas a positive skew would indicate that links tend to be "influencers"). This phenomenon is particularly pronounced for online social networks and for human brain networks. These networks also have perpendicular kurtosis above 3.0, indicating a tendency for links to sit further away from the diagonal, rather than clustering around it, showing that the memory of these temporal networks are disproportionately driven by a small number of links. A similar behavior

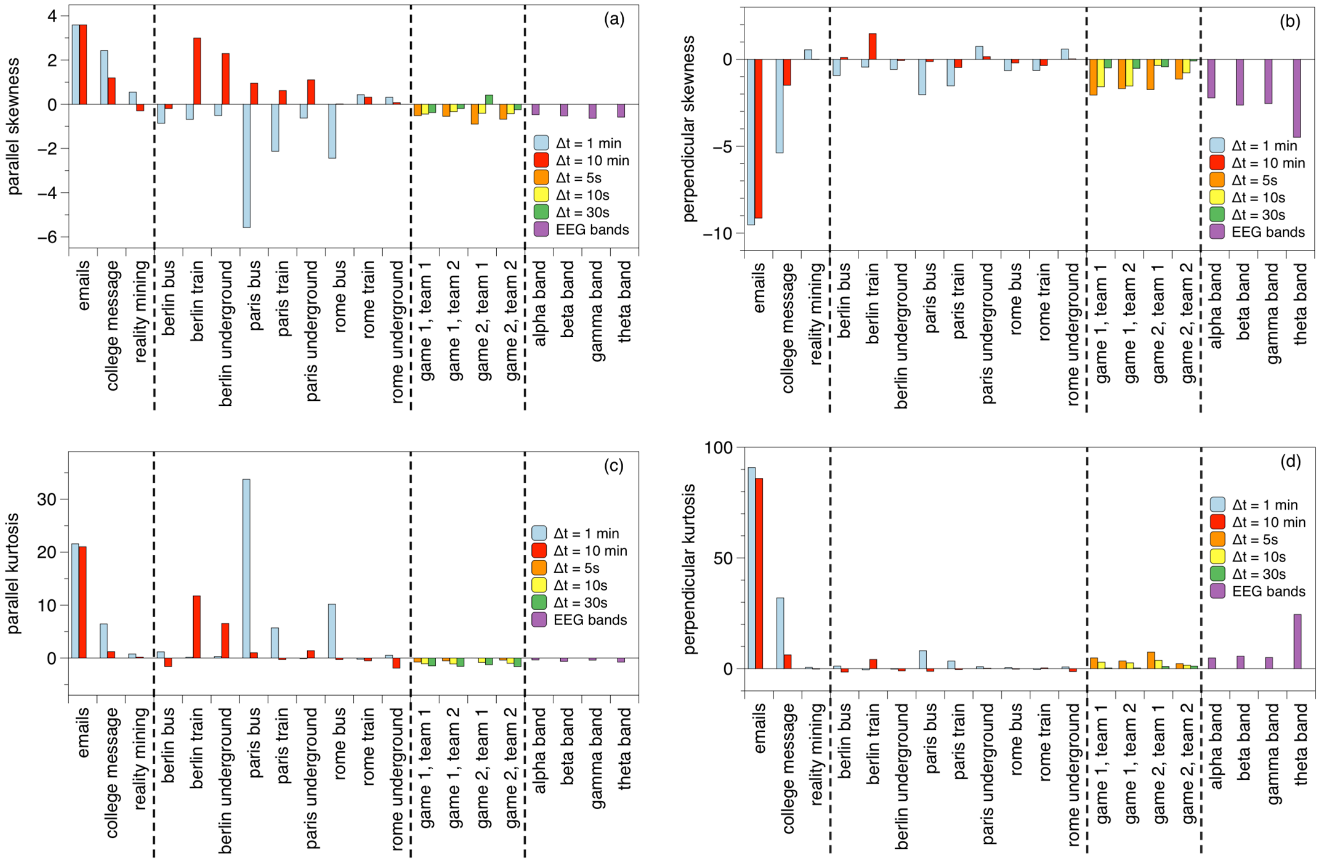

FIG. S14. **Skewness and kurtosis of the parallel and perpendicular distributions.** We plot the skewness and kurtosis of the coordinates h^α (parallel to the diagonal) and d^α (perpendicular to the diagonal) for all the real-world temporal networks, and various timescales. The timescales used are $\Delta t = 1$ min (blue) and $\Delta t = 10$ min (red) for the networks in the "social" and "transportation" categories, $\Delta t = 5$ (orange) , 10 (yellow) and 30 seconds (green) for the "football" category, and the four frequency bands $\alpha, \beta, \gamma, \theta$ of the "brain" category.

has also been observed in proximity networks from football matches, and in most of the transportation systems we have studied.

Altogether, analysis of the co-memory matrix (and its projection in the co-memory histogram and the $(\langle\Omega\rangle^\alpha_{\text{in}}, \langle\Omega\rangle^\alpha_{\text{out}})$ plane) at different resolutions provide insights on the microscopic memory heterogeneity displayed by real temporal networks, providing a simple method for discrimination between self-organised, online and offline engineered networks, and highlighting different and interpretable memory timescales. In particular, our results certify that while all networks considered display memory as estimated via $\Omega_{\text{eff}}(\mathcal{G})$, online social networks have a weaker and more homogeneous microscopic memory kernel than infrastructure transportation networks, and in between we find the case of the social contact network (RM) –a mix case which is 'social', like the first group, but 'offline' like the second–, and this latter unveils the presence of two different memory timescales, probably related to two distinct mechanisms of social interaction between the university students: casual social interaction vs sharing a class together.

E. Assessing the impact of finite-size effects and non-stationarities in the memory estimation

To round-off the analysis on real-world empirical networks, we now examine two possible effects that may be present and affect the interpretation of memory estimates, namely finite size effects and possible sources of non-stationarities in the temporal network dynamics.
The former relates to the fact that when dealing with long memory, one needs to have access to long time series to accurately estimate such memory. A too short sequence can destroy long term memory and make any estimation protocol unable to estimate such memory length.
The latter relates to the fact that if the intrinsic dynamics of the temporal network –and more concretely, its memory–

changes over time or over multiple timescales, then estimation of such memory over different time windows might yield different results. Similarly, if the intrinsic dynamics of the temporal network is reasonably stationary, then the memory estimation should be consistent when measured at different time windows.

The former effect is, by construction, an unavoidable limitation to any estimation method, so in rigor when estimating memory we should regard such estimation as a lower bound. From a practical point of view this is not really a problem, insofar the effect of 'feeling" a certain amount of memory is also limited by such finite size effects. In other words, if a certain dynamics running on top of a temporal network will be affected by the intrinsic memory of that temporal network, then the amount of such memory the running dynamics will feel is to some extent related to the timescales of both dynamics.

The latter effect (non-stationarity of intrinsic temporal network dynamics) could be in principle more problematic when analysing real-world temporal networks, as we don't have a priori a guarantee that such dynamics is sufficiently stationary. It is however important to highlight that we are referring here not to non-stationary dynamics, but to a non-stationary memory. Intuitively speaking, the latter is arguably more stable than the former.

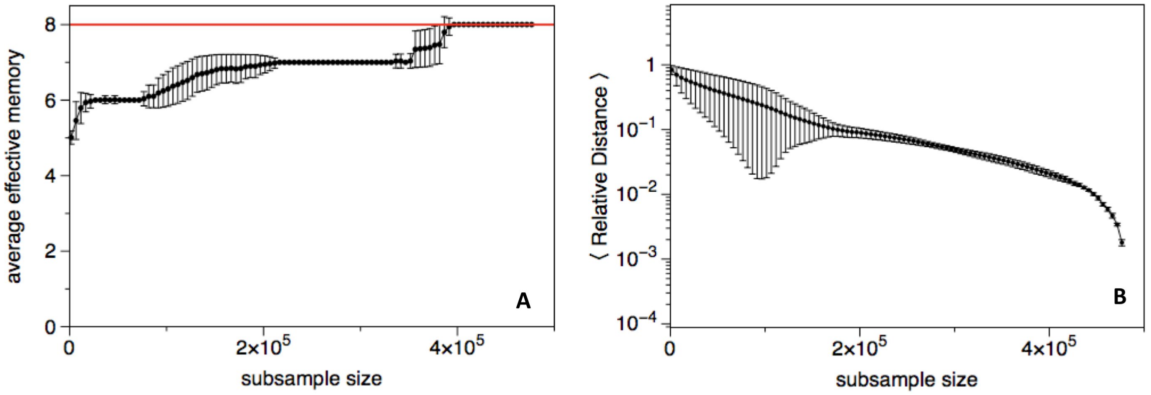

FIG. S15. **Finite-size effects.** Estimation of the effective memory (panel A) and average relative distance (panel B) of subsamples of the RM temporal network with a number of time snapshots N smaller than the full dataset (which has $N_{tot} = 481568$ time snapshots). Different subsamples with the same size are obtained by sliding a window of size N over the full dataset. Results are then averaged over all subsamples of a given size. From (A), we observe that estimation of the effective memory is mildly affected by finite-size effects, as already with very short sizes ($N = 10^3$, i.e. circa 1% of the size of the full dataset) we already estimate large effective memory $\Omega_{\text{eff}} = 5$. From (B), we find that the relative distance (see text for definition) of the whole co-memory matrix to the one estimated for the full dataset decays fast with the subsample size (notice the plot is in log-linear scale).

To explore these issues in a practical setting, we consider the case of the offline social temporal network provided within the Reality Mining experiment (RM). We have chosen this real-world temporal network since it provides a test case where intrinsic dynamics is already rich (the memory shape is heterogeneous and has many entries with large memory, and the effective memory is indeed very large), so results on this example can be considered as a worst-case scenario. We have made two analysis:

1. **Finite-size effects**: we subsample the original temporal network (which has $N_{tot} = 481568$ time snapshots) in such a way that we extract a shorter temporal network with $N < N_{tot}$ time snapshots, and we then estimate the memory shape and effective memory on this shorter signal. At the same time, for a fixed subsample size N, we extract different realisations by varying the starting point of the subsample network (that is akin to creating a sliding window). We can then average results for a given subsample size. First, we report the average effective memory of a given subsample size (averaging over different initial points to assess non-stationarity), and we also provide standard deviations with respect to the average value in each case. Results are shown in Panel (A) of Figure S15. As we can see, the estimation asymptotically reaches the estimated effective memory of the full dataset ($\Omega_{\text{eff}} = 8$). It is important to observe that already with very short subsample sizes (10^3 time snapshots) the estimated effective memory is already large, so we can conclude that our method is capable of estimating long memory even when a severe subsampling is performed.
   To further assess how the estimation of the microscopic memory shape changes with subsample size and start-

ing point, we have also computed the L_1 (Manhattan) distance between the full memory shapes (co-memory matrices). If properly rescaled by the L_1 norm to convert this into a relative distance RD

$$RD = \frac{\sum_{ij} |\mathbb{M}_{ij}^{\text{subsample}} - \mathbb{M}_{ij}^{\text{full}}|}{\sum_{ij} \mathbb{M}_{ij}^{\text{full}}} \tag{83}$$

RD gives us an indication of how, on average, each entry of the co-memory matrix differs from its full dataset counterpart, relative to the average entry of the co-memory matrix of the full dataset, and can be understood as an scalar estimate of the relative distance between both memory shapes. One can then average RD over all subsamples of a given size (i.e., averaging over different starting points). Results are plotted in panel (B) of Fig S15, showing that the distance fastly decays with subsample size (note the plot is in log-linear scale).

2. **Non-stationary effects:** in Fig. S16 we have also reported the estimated memory of individual subsamples of a given size, for different starting windows. Panel (A) of tis figure reports the estimated effective memory, whereas panel (B) reports the relative distance of each individual subsample co-memory matrix to the one estimated from the full dataset. Several observations can be depicted:
- the estimation of the effective memory is stable and does not vary across different starting windows. This suggests that the subset of links that carry the largest memory of the temporal network have a reasonably stationary dynamics, and that such memory estimation is therefore not affected by sources of non-stationarity in the data.
- the estimation of the relative distance between the memory shape of the subsample and the full dataset, for large subsample sizes, is itself constant over different starting points. However, for smaller subsample sizes (short time windows), we can appreciate a change (a drop in relative distance) within the first 10^5 datapoints. This suggests that the intrinsic dynamics of the temporal network shows some difference in the first 10^5 time points than after that. To understand this source of non-stationarity, we come back to the temporal network and compute, for a sliding subsample size of 10^5, the observed total number of links as a function of the starting point. This is reported in panel (C) of Fig. S16. We can clearly see that such link density varies in the same way as the relative distance and thereby we conclude that this source of non-stationarity in the relative distance is indeed reminiscent of the nonstationarity showing up in the network during the initial stages of the recording.

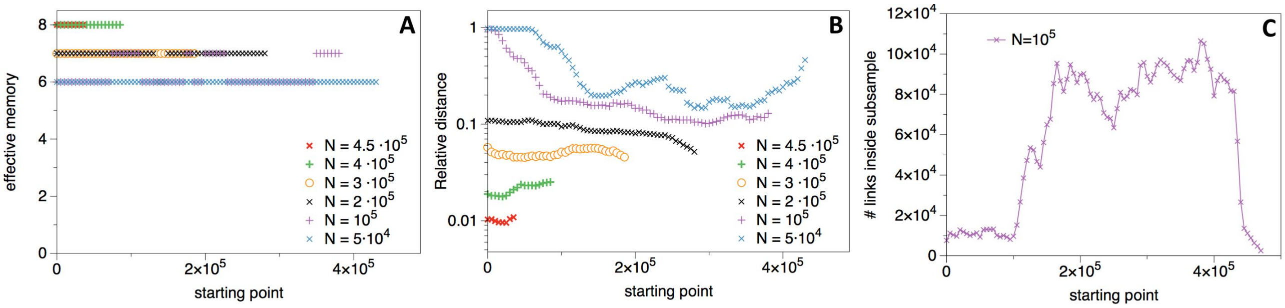

FIG. S16. **Non-stationarity effects** Effective memory (panel A) and relative distance (panel B) of subsamples of size N as a function of the starting point of the subsample within the full dataset. Estimation of the effective memory is almost independent of the starting point, which suggests that the subset of links that carry the largest memory have a reasonably stationary dynamics. The relative distance of the subsample to the full dataset (panel B) is also somewhat independent of the starting point for large N (up to subsamples of about 40% of the full dataset). For shorter subsamples, we can see new regimes emerging: for $N = 10^5$ the memory shape of the initial windows of that size show a substantial distance to the one in the full dataset. This can be explained if looking at the total number of active links in the whole subsample (panel C), which for $N = 10^5$ shows a marked change after the first 10^5 time points.

As a general conclusion, the analysis presented above suggests that finite-size effects only have a small effect on the estimation of effective memory, and a small to moderate effect in the estimation of the microscopic shape. Again, finite size effects are unavoidable and thus this is an expected result. At the same time, non-stationarities present in the data can be detected when computing the full memory shape over short time windows, but the effective memory is somewhat independent, which means that the subset of active links that carry largest memory have a more stationary dynamics.

VI. METHOD IMPLEMENTATIONS

As part of this work we have provided a number of implementations of our approach to finding the memory of a temporal network, or the distribution of its co-orders, as based on the above mentioned efficient determination criterion (EDC) given either a file containing a time stamped edge list or the input from a suitable function. The intention here is to remove the complexity associated with implementing our method in an efficient way, and thus allow any future studies in this area to forge ahed without such an overhead. To this end we have provided versions in the following languages, each with their own advantages and disadvantages:

- C++
- Java
- Python 3.6
- Python 2.7
- Rust

The versions written in Python, and to some extent Java are intended to be used for testing of smaller data sets and prototyping of other experiments. This is because though the languages are common and their implementations are hopefully easy to understand, they (again with the possible exception of Java) lack the raw speed and low memory overhead of the other languages.
The C++ versions (provided with both parallel and serial approaches) are intended to be as fast as possible while maintaining the lowest possible memory overhead, and so is well suited to handling larger networks or the running of multiple experiments at once.
The Rust version (provided with both parallel and serial approaches) is also intended to be as fast as possible, but does have a higher memory overhead when compared to the C++ version (though this is still the second lowest memory usage), as such it is suited to larger networks, but may not be as good as C++ for running multiple experiments at once. We have however provided a parallel implementation in Rust, which, provided there is no problem with memory requirements, is the fastest available after the C++ parallel implementation.
The Java version is intended to be usably fast in any situation and with a moderate memory overhead, while being easy to work with.

An important consideration with our approach is the scaling of runtime with the size of the network. While one would expect networks with higher numbers of links to take longer to study, it is important to understand the practical implications of this. Hence we analyse this behaviour here. The scaling of our method for estimating the memory of a temporal network relies on two things: (a) the size of the backbone of the temporal network being analysed and (b) the behaviour of the co-order estimator with respect to the number of time steps in the temporal network data being studied. The first of these factors is well understood: the runtime scales as the square of the number of links in the backbone. If the backbone is the whole network, this would be L^2. But in practice not all links of the network will have an active dynamics –and as such, only the dynamically active links will contribute to nontrivial microscopic memory–, so heuristically we can severely cap this contribution, however that needs to be done in a case-by-case basis, therefore worst case scenario is still L^2 when all links of the network are active.
The second factor is instead related to the estimator used. Since our (EDC based) approach requires us to run through each time step in the data for each possible value of the memory p, we expect a (worstcase) runtime that scales as $O(L^2pT)$, and when the number of active links $L_a \leq L$, we have a runtime that scales as $O(L_a^2pT)$.

To have an idea of how this scaling translates into actual runtime, we have initially tested the runtime of each implementation by finding the effective memory of the edge list associated with the (EM) data set at a resolution of $\Delta t = 60$ seconds. This comprises of 100 links over 390507 time steps. These tests were run on a desktop PC (Ubuntu 18.04.3 LTS (64-bit)), with a intel Core i7-6700k (4.00GHz, 4 core) processor, and 32GB of memory. Each test was repeated 10 times and the minimum result taken.
Then, we have also tested the performance of the fastest of our implementations (C++ parallel) for simulated DARN(p) networks. We first fix memory length $p = 5$, memory strength $q = 0.9$, and link frequency $y = 0.2$, and some number of nodes N which consequently fixes the number of links L, then time the generation of the network for 10000 time steps, and memory estimation of the network. This is then repeated ten times for each L and the minimum time taken, allowing us to plot the minimum run time as a function of the number of links in the network. This provides us with a rough estimate of run times for any network with a maximum co-order of 5 and 10000 time steps. The results of this are shown in Fig.S17. We see that the runtime scales with the number of active links L as L^2. However,

Version	Minimum time (seconds)
C++ (parallel implementation, gcc9 -o3)	12.782
Rust (parallel implementation, rustc 1.39, llvm 9.0, opt-level 2)	14.82
C++ (gcc9 -o2)	59.42
Rust (serial implementation, rustc 1.39, llvm 9.0, opt-level 3)	61.5
Java (OpenJDK 8)	62.1
Python 3.7 (Parallel implementation)	4203
Python 2.7	7854
Python 3.7 (Serial implementation)	12690

TABLE V. Runtimes for estimating the effective memory of the EM data set with $\Delta t = 60 seconds$ (see text for details).

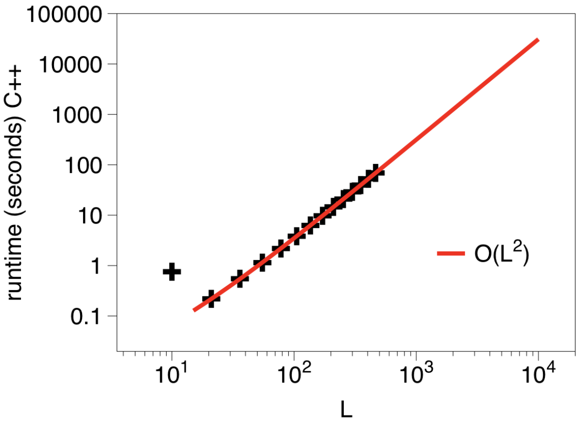

FIG. S17. **Runtime scaling with number of links L.** If we discard the first point, the best fit to an order-2 polynomial is $f(x) = -0.0008 + 0.0039L + 0.00031L^2$, with $R^2 = 1$ (red solid line). The best power law fit gives a scaling $\sim L^{1.875}$.

it is worth noting that even on a relatively outdated computer (at the time of writing) we can achieve manageable runtimes even for large L, with $L = 4950$ taking just over two hours. Given that this implementation scales linearly with the available threads (e.g. going from the current 4 core, 8 thread processor to an equivalent 8 core 16 thread processor will half the run time) we believe that the method scales well for available computing resources. For access to the code and a more in depth description of its implementation see `github.com/oewilliams/temp-net-memory`.

Supplementary References

[1] Gagniuc, P. A. *Markov chains: from theory to implementation and experimentation* (John Wiley & Sons, 2017).
[2] Tong, H. Determination of the order of a markov chain by akaike's information criterion. *Journal of applied probability* **12**, 488–497 (1975).
[3] Schwarz, G. *et al.* Estimating the dimension of a model. *The annals of statistics* **6**, 461–464 (1978).
[4] Dorea, C. C., Goncalves, C. R. & Resende, P. Simulation results for markov model seletion: Aic, bic and edc. In *Proc. World Congress on Engineering and Computer Science*, vol. 2, 899–901 (2014).
[5] Zhao, L., Dorea, C. & Gonçalves, C. On determination of the order of a markov chain. *Statistical inference for stochastic processes* **4**, 273–282 (2001).
[6] Van der Heyden, M. J., Diks, C. G., Hoekstra, B. P. & DeGoede, J. Testing the order of discrete markov chains using surrogate data. *Physica D: Nonlinear Phenomena* **117**, 299–313 (1998).
[7] Katz, R. W. On some criteria for estimating the order of a markov chain. *Technometrics* **23**, 243–249 (1981).
[8] Papapetrou, M. & Kugiumtzis, D. Markov chain order estimation with parametric significance tests of conditional mutual information. *Simulation Modelling Practice and Theory* **61**, 1–13 (2016).
[9] Granger, C. W. Investigating causal relations by econometric models and cross-spectral methods. *Econometrica: Journal of the Econometric Society* 424–438 (1969).
[10] Jacobs, P. A. & Lewis, P. A. Discrete time series generated by mixtures. iii. autoregressive processes (dar (p)). Tech. Rep., NAVAL POSTGRADUATE SCHOOL MONTEREY CALIF (1978).
[11] Pearl, J. *Reverend Bayes on inference engines: A distributed hierarchical approach* (Cognitive Systems Laboratory, School

of Engineering and Applied Science, University of California, Los Angeles, 1982).
[12] Yedidia, J. S., Freeman, W. T. & Weiss, Y. Understanding belief propagation and its generalizations. *Exploring artificial intelligence in the new millennium* **8**, 236–239 (2003).
[13] Weiss, Y. & Freeman, W. T. Correctness of belief propagation in gaussian graphical models of arbitrary topology. In *Advances in neural information processing systems*, 673–679 (2000).
[14] Baron, D., Sarvotham, S. & Baraniuk, R. G. Bayesian compressive sensing via belief propagation. *IEEE Transactions on Signal Processing* **58**, 269–280 (2009).
[15] Ihler, A. & McAllester, D. Particle belief propagation. In *Artificial Intelligence and Statistics*, 256–263 (2009).
[16] Felzenszwalb, P. F. & Huttenlocher, D. P. Efficient belief propagation for early vision. *International journal of computer vision* **70**, 41–54 (2006).
[17] Lokhov, A. Y., Mézard, M., Ohta, H. & Zdeborová, L. Inferring the origin of an epidemic with a dynamic message-passing algorithm. *Physical Review E* **90**, 012801 (2014).
[18] Yedidia, J. S., Freeman, W. T. & Weiss, Y. Constructing free-energy approximations and generalized belief propagation algorithms. *IEEE Transactions on information theory* **51**, 2282–2312 (2005).
[19] Opper, M., Winther, O. *et al.* From naive mean field theory to the tap equations. *Advanced mean field methods: theory and practice* 7–20 (2001).
[20] Kabashima, Y. A cdma multiuser detection algorithm on the basis of belief propagation. *Journal of Physics A: Mathematical and General* **36**, 11111 (2003).
[21] Neirotti, J. P. & Saad, D. Improved message passing for inference in densely connected systems. *EPL (Europhysics Letters)* **71**, 866 (2005).
[22] Murphy, K. P., Weiss, Y. & Jordan, M. I. Loopy belief propagation for approximate inference: An empirical study. In *Proceedings of the Fifteenth conference on Uncertainty in artificial intelligence*, 467–475 (Morgan Kaufmann Publishers Inc., 1999).
[23] Yedidia, J. S., Freeman, W. T. & Weiss, Y. Generalized belief propagation. In *Advances in neural information processing systems*, 689–695 (2001).
[24] Ihler, A. T., John III, W. F. & Willsky, A. S. Loopy belief propagation: Convergence and effects of message errors. *Journal of Machine Learning Research* **6**, 905–936 (2005).
[25] Cantwell, G. T. & Newman, M. E. J. Message passing on networks with loops (2019). 1907.08252.
[26] Passarino, G. & Veltman, M. One-loop corrections for e+ e- annihilation into μ+ μ- in the weinberg model. *Nuclear Physics B* **160**, 151–207 (1979).
[27] t Hooft, G. & Veltman, M. One-loop divergencies in the theory of gravitation. In *Annales de l'IHP Physique théorique*, vol. 20, 69–94 (1974).
[28] Mostepanenko, V. M. & Trunov, N. *The Casimir effect and its applications* (Oxford University Press, 1997).
[29] Jaffe, R. Casimir effect and the quantum vacuum. *Physical Review D* **72**, 021301 (2005).
[30] Russo, J. G., Susskind, L. & Thorlacius, L. End point of hawking radiation. *Physical Review D* **46**, 3444 (1992).
[31] Czarnecki, A., Jentschura, U. D. & Pachucki, K. Calculation of the one-and two-loop lamb shift for arbitrary excited hydrogenic states. *Physical review letters* **95**, 180404 (2005).
[32] Williams, O. E., Lillo, F. & Latora, V. Effects of memory on spreading processes in non-markovian temporal networks. *New Journal of Physics* **21**, 043028 (2019).
[33] Goh, K.-I. & Barabási, A.-L. Burstiness and memory in complex systems. *EPL (Europhysics Letters)* **81**, 48002 (2008).
[34] Williams, O. E., Lillo, F. & Latora, V. How auto- and cross-correlations in link dynamics influence diffusion in non-markovian temporal networks (2019). 1909.08134.
[35] Panzarasa, P., Opsahl, T. & Carley, K. M. Patterns and dynamics of users' behavior and interaction: Network analysis of an online community. *Journal of the American Society for Information Science and Technology* **60**, 911–932 (2009).
[36] Michalski, R., Palus, S. & Kazienko, P. Matching organizational structure and social network extracted from email communication. In *Lecture Notes in Business Information Processing*, vol. 87, 197–206 (Springer Berlin Heidelberg, 2011).
[37] Eagle, N. & Pentland, A. S. Reality mining: sensing complex social systems. *Personal and ubiquitous computing* **10**, 255–268 (2006).
[38] Kujala, R., Weckström, C., Darst, R. K., Mladenović, M. N. & Saramäki, J. A collection of public transport network data sets for 25 cities. *Scientific data* **5**, 180089 (2018).
[39] Fallani, F. D. V. *et al.* Persistent patterns of interconnection in time-varying cortical networks estimated from high-resolution eeg recordings in humans during a simple motor act. *Journal of Physics A: Mathematical and Theoretical* **41**, 224014 (2008).
